# Tunable BIC metamaterials with Dirac semimetals

**DOI:** 10.1515/nanoph-2025-0358

**Published:** 2025-10-29

**Authors:** Xiaoyong He, Wenhan Cao, Fangting Lin

**Affiliations:** Department of Physics, Mathematics & Science College, 12544 Shanghai Normal University, No. 100 Guilin Road, Shanghai, 200234, China; School of Information Science and Technology, ShanghaiTech University, 393 Middle Huaxia Road, Shanghai, 201210, China; Shanghai Key Lab for Astrophysics, No. 100 Guilin Road, Shanghai, 200234, China

**Keywords:** Dirac semimetal, metamaterials, BIC, terahertz, tunable

## Abstract

Proposed by von Neuman and Wigner in 1929, bound states in the continuum (BIC) exhibit the merits of ultrahigh *Q–factor* and strongly confined modes, solving the inherent high dissipation of metamaterials (MMs) and plasmonic devices. Dirac semimetal (DSM) possesses the advantages of high carrier mobility and outstanding tunable properties, which provides avenues for the design of performance functional devices. This review focuses on recent progresses of the DSM (graphene and 3D Dirac semimetals, *e.g.* Cd_3_As_2_) and other novel materials (*e.g.* MoS_2_, borophene, GaSe) based BIC MMs, including the effects of Fermi levels, resonators types, and operation frequency ranges. Some related interesting phenomena, such as tunable Fano resonance, strong epsilon-nearly-zero and nonlinear harmonic effects, together with a brief prospect on the future development trends of DSM MMs, have been given and discussed. This work also provides a useful guideline to understand the tunable mechanism of the DSM devices and develop high performance functional devices applied in the fields of wireless communications, security detection, and sub-millimeter astronomical observations, *e.g.* filters, modulators and polarizers.

## 1 Introduction

With properly designed compositions and arrangements of subwavelength building blocks (also known as unit cells, meta-molecules, meta-atoms), metamaterials (MMs) are artificially engineered structures manifesting exotic electromagnetic phenomena, and exhibiting extraordinary properties in response to electromagnetic, acoustic, and thermal waves [1], [2], [3], [4], [5], [6]. Specifically, electromagnetic MMs are capable of significantly enhancing light–matter interactions and manifesting unique electronic responses determined by the micro-structures rather than the material composition, which provides a promising platform for the exploration of many phenomena, such as negative refractive index, super–focusing, and extraordinary transmission [7], [8], [9], [10], [11], [12], [13], [14]. As a typical two–dimensional (2D) form of metamaterials, metasurface offers an effective means to manipulating incident waves through modification of the amplitude, polarization, phase, as well as beam shape of light [15], [16], [17], [18], [19], [20], [21], [22], [23], [24]. Although conventional metal plasmonic MMs have been intensively investigated, their performances are seriously hindered by the inevitable Ohmic and radiation losses. Additionally, dielectrics with high refractive index also provide an efficient means to the design of MMs devices with high performances, inhibiting the merits of low thermal conductivity, excellent high temperature performances, and relatively easily fabrication processes [25], [26], [27], [28], [29], [30], [31], [32], [33], [34].

Nowadays, bound state in the continuum (BIC) has received a great deal of attention owing to their fascinating properties, such as perfect confinement of field energy above the light cone. BIC lies inside the continuum and coexist with extended states, but they remain perfectly localized without any radiation. As an ideal non-radiative discrete state coexisted within a continuum spectrum, BIC was originally proposed by von Neuman and Wigner in 1929, and envisioned a theoretically infinite *Q–factor* with a zero line-width [35]. Notably, in 1975 F. H. Stillinger and D. R. Herrick proposed a superlattice structure to construct the bound states, exciting a revival research attention of BIC resonance [36]. Later, based on the AlInAs/GaInAs semiconductor hetero-structures grown by molecular beam epitaxy, in 1992, Capasso’s group experimentally demonstrated direct evidence of BIC via the infrared absorption measurement, which confirmed that the bound state was spatially localized by Bragg reflections and revealed an isolated transition within a quantum well to a bound state at an energy greater than the barrier height [37]. First proposed in quantum mechanics, BIC resonance is a general wave phenomenon and identified in electromagnetic waves, acoustic waves in air, water waves and elastic waves in solids [38], [39], [40], [41]. The explored material systems include piezoelectric materials, dielectric photonic crystals, optical waveguides and fibers, quantum dots, graphene and topological insulators [42], [43], [44], [45], [46]. From a topological physical view, BIC corresponds to the polarization singularities carrying integer winding numbers around the vortex center in a radiative polarization vector field. The topological nature of BIC opens up a unique avenue for nonlocal light manipulation [47], [48], [49], [50], [51], [52].

**Figure 1:**
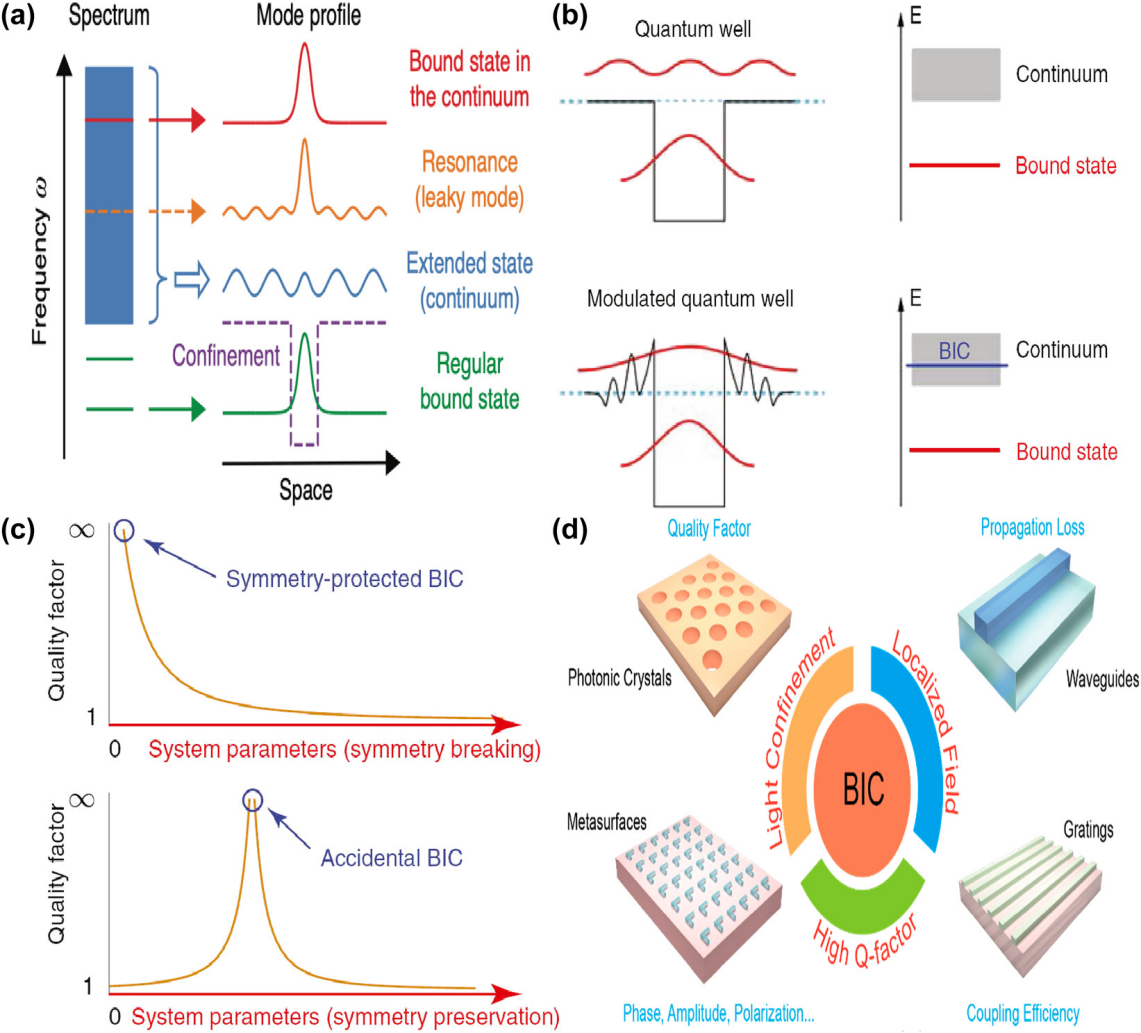
The physical mechanisms and applications of BIC resonance. (a) Several kinds of resonant states in an open system, spatially extended states (blue), discrete levels of bound states (green), a confining structure or potential (dashed line), states inside the continuous spectrum couple to the extended waves and radiate (orange), and bound states in the continuum (BIC, red) [38]. Figure adopted with permission from Ref. [38]. Copyright 2016, Springer Nature. (b) Schematic illustration of origin of BICs in quantum mechanical systems [59]. (c) Different mechanisms of BIC formation, *i.e.* symmetry protection (upper panel) and parameter tuning (lower panel) [59]. Figures adopted with permission from Ref. [59] under License CC BY 4.0. (d) A graphical description of the core characteristics of BIC (annotated in red) and the associated applications in various photonic structures (annotated in blue) [80]. Figure adopted with permission from Ref. [80] under License CC BY 4.0.

The mechanisms of BIC resonances are shown in Figure 1(a), the extended state (blue line) exists across a continuous range of frequencies, the green line is the discrete level of conventional bound states that have no access to radiation channel. Inside the continuum, the orange line locally resembles a bound state and couples to the extended waves and leak out, which can be expressed with a complex frequency, *ω* = *ω*_0_–*i*γ, in which the real part *ω*_0_ is the resonant frequency and the imaginary part *γ* represents the leakage rate. Despite the fact that an ideal BIC does not exist, a quasi-BIC resonance with sharp peak and finite *Q–factor* can be excited by altering structural parameters, incident light angle or introducing small perturbations [43], [53], [54], [55], [56], [57], [58], [59]. On the base of formation mechanism, BIC is generally divided into the symmetry-protected (S–P) BIC with the wave vector at the Γ point in momentum space and the accidental BIC resonance with that at the non-Γ point [60], [61], [62], as given in Figure 1(b) and (c). In general, the S–P BIC is safeguarded by mode symmetry against energy leakage and achieved through oblique incidence or symmetry breaking of nanostructures. In contrast, the accidental BIC is achieved by tuning the geometric parameters. Accidental quasi-BICs can also be achieved with comparatively simple architectures [63], [64], [65], [66]. Regarded as a destructive interference in two radiating channels from two coupled resonators, some accidental BICs are known as Friedrich–Wintgen (FW) BIC. However, if the two resonators are separated by some distance, the accidental BIC becomes Fabry–Perot (FP) BIC [65], [67], [68], [69], [70], [71], [72], [73], [74]. Quasi-BIC in MM structure usually excites many resonant multipoles, such as the electric dipole (ED), magnetic dipole (MD), magnetic quadrupole (MQ), and toroidal dipole (TD), which can be realized through breaking the structural symmetry or altering the incident angle [75], [76], [77], [78], [79], [80].

Nowadays, with the advantages of ultrahigh *Q–factor*, strongly confined modes, and novel topological characteristics, BIC resonances are universally explored in various functional devices [81], [82], [83], [84], [85], [86], [87], [88], [89], which can be found in Figure 1(d). From the perspective of practical applications, especially in the fields of highspeed wireless communication, sub-millimeter astronomical observation, and security detection, it is crucial to develop dynamical manipulation of highly efficient functional devices, such as lasers, sensors, filters and low-loss fibers [90], [91], [92], [93], [94]. However, when it comes to conventional metal MMs, the large carrier concentration usually accompanies a high dissipation and poor tunable property. Nowadays, novel topological matters [95], [96], [97], [98], [99], [100], *i.e.* topological insulators, topological superconductors, and topological semimetals (TSM), have attracted extensive attentions. With linear dispersion and topologically protected mode degeneracy, in the momentum space topological semimetals possess robust nodal areas between conduction and valence bands, which mainly include Dirac semimetals (DSM), Weyl semimetals (WSM) [101], [102], [103], [104], [105], [106], and nodal-line semimetals [107], [108], [109], [110], [111]. Arranged in a hexagonal lattice, as a typical example of 2D materials, graphene exhibits novel physical and chemical properties, such as higher carrier mobility, strong mode confinement and high order harmonic effects, especially the good tunable properties by an applied voltage, magnetic field or chemical doping [112], [113], [114], [115], [116], [117], [118], [119], [120], [121], [122], [123]. Of course, there also exist many other kinds of 2D materials, such as borophene, black phosphorus, and transition metal dichalcogenide (TMD) [124], [125], [126], [127], [128], [129], [130], [131], which also showcase outstanding tunable properties and strong nonlinear optical properties.

**Figure 2:**
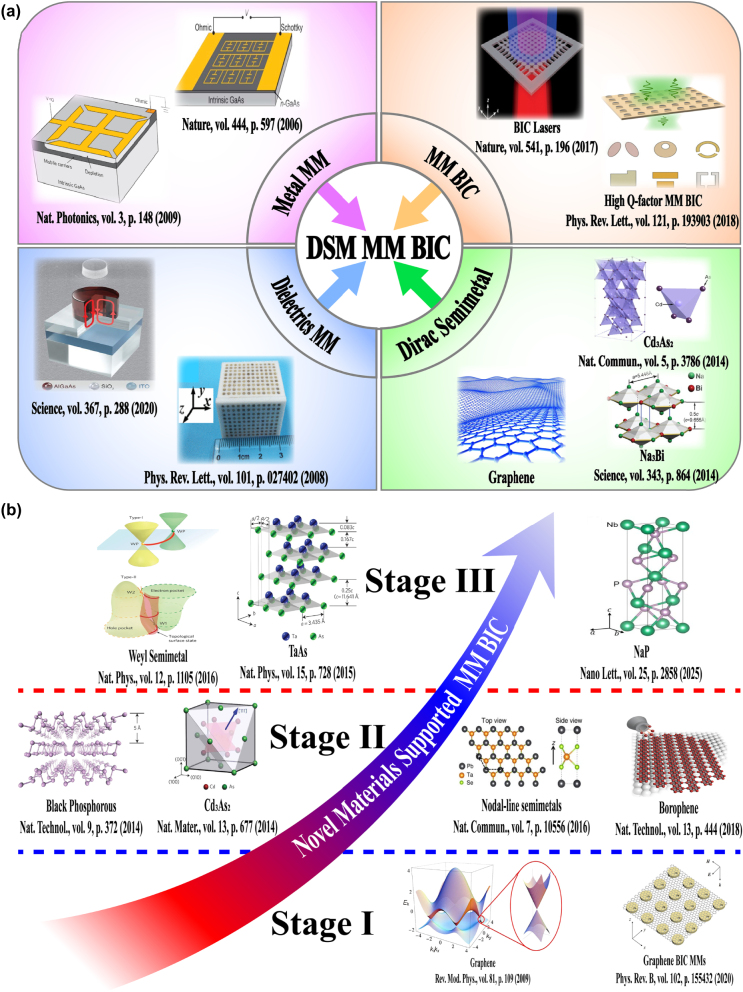
The main application fields and possible future development trend of DSM supported tunable metasurface BIC resonance. (a) The sketch diagram of DSM supported tunable MMs BIC resonances [1], [2], [4], [7], [40], [41], [140], [141]. Figures adopted with permission from Ref. [1], Copyright 2006, Springer Nature. Figures adopted with permission from Ref. [2], Copyright 2009, Springer Nature. Figure adopted with permission from Ref. [4], Copyright 2020, American Association for the Advancement of Science. Figure adopted with permission from Ref. [7], Copyright 2008, APS. Figures adopted with permission from Ref. [40], Copyright 2017, Springer Nature. Figure adopted with permission from Ref. [41], Copyright 2018, APS. Figure adopted with permission from Ref. [140], Copyright 2014, American Association for the Advancement of Science. Figures adopted with permission from Ref. [140], Copyright 2014, Springer Nature. Figure adopted with permission from Ref. [141], Copyright 2014, Springer Nature. (b) The development future trend for the novel materials supported MMs BIC resonances [97], [98], [99], [101], [103], [105], [120], [132], [147]. Figure adopted with permission from Ref. [97], Copyright 2014, Springer Nature. Figure adopted with permission from Ref. [98], Copyright 2018, Springer Nature. Figure adopted with permission from Ref. [99], Copyright 2015, Springer Nature. Figure adopted with permission from Ref. [101], Copyright 2016, Springer Nature. Figure adopted with permission from Ref. [103] under License CC BY 4.0. Figure adopted with permission from Ref. [105], Copyright 2016, Springer Nature. Figure adopted with permission from Ref. [120] under License CC BY 4.0. Figure adopted with permission from Ref. [132], Copyright 2009, APS. Figure adopted with permission from Ref. [147], Copyright 2014, Springer Nature.

Unfortunately, the tunable properties of the 2D materials are inherently limited by the thin thickness [132], [133], [134], [135], [136], [137], [138], [139]. Interestingly, as a novel class of functional materials, three-dimensional (3D) Dirac semimetal, *e.g.* cadmium arsenide (Cd_3_As_2_) and Na_3_Bi, can be regarded as an analogy of “3D graphene”. The band structure of 3D DSM contains the so-called Dirac points, in which conduction and valence bands overlap with each other, resulting into the linear energy-momentum dispersions, as well as nearly zero effective mass [140], [141], [142], [143], [144], [145], [146]. In contrast to graphene membrane, 3D DSM also has several advantages [147], [148], [149], [150], [151], [152]. The Fermi velocities and mobilities of 3D DSM are larger than those of graphene, resulting into better plasmonic and tunable properties simultaneously. Additionally, 3D DSM surmounts the restriction of thickness and enjoys an additional structural degree-of-freedom (DoF) in the construction of functional devices. 3D DSM is also more stable and less susceptible to the influences of surrounding environmental defects and substrate materials. As mentioned above, DSM is identified as a good platform to develop novel tunable functional device in a wide frequency range from visible light to terahertz waves [153], [154], [155], [156], [157], [158], [159]. For instance, by introducing hyperbolic metamaterials into one-dimensional photonic crystals, the “hyper-crystal” provides a novel avenue for manipulating the light–matter interactions and explore the novel DSM devices [160], [161], [162]. Additionally, different from the conventional structures, BIC resonance confines light in the continuum and results into an ultra-small mode volume in the range of nanoscale, enhancing the interaction between light and matter by orders of magnitude. Thus, by integrating DSM with BIC metasurface, as given in Figure 2(a), many interesting phenomena are demonstrated, such as sharp resonant curves with ultrahigh *Q–factor*, obvious epsilon-nearly-zero (ENZ) phenomenon, and strong high order harmonic effects. Nowadays, there is a rapid development trend in the aspect of DSM supported BIC metasurface. Here, we provide a brief introduction in the following sections, mainly including the effects of the Fermi level, different kinds of tunable media, and the configuration of differently shaped resonators.

## 2 Graphene supported BIC metamaterials

Graphene can be generally considered as a 2D material and described by a surface conductivity *σ*_*g*_, which is related to the radiation frequency *ω*, chemical potential *μ*_*c*_ (Fermi level, *E*_*f*_), environmental temperature *T*, and relaxation time *τ*. The conductivity of monolayer graphene can be obtained from the Kubo formula [163], [164], [165]:

(1) $$\begin{array}{ll} \sigma \left(\omega ,\mu_{c},\tau ,T\right) & =\sigma_{\text{intra}}+\sigma_{\text{inter}}=\frac{je^{2}\left(\omega -j\tau^{-1}\right)}{\pi \hbar^{2}}\\ & \;\times \left[\frac{1}{{\left(\omega -j\tau^{-1}\right)}^{2}}\overset{\infty }{\underset{0}{\int }}\frac{\partial f_{d}\left(\varepsilon \right)}{\partial \varepsilon }-\frac{\partial f_{d}\left(-\varepsilon \right)}{\partial \varepsilon }d\varepsilon \right.\\ & \;-\left.\overset{\infty }{\underset{0}{\int }}\frac{f_{d}\left(-\varepsilon \right)-f_{d}\left(\varepsilon \right)}{{\left(\omega -j\tau^{-1}\right)}^{2}-4{\left(\varepsilon /\hbar \right)}^{2}}d\varepsilon \right] \end{array}$$

where *f*_*d*_(*ε*) is the Fermi–Dirac distribution, *j* is the imaginary unit, *ε* is the energy of the incident wave, and ℏ is the reduced Planck’s constant, *τ* is the scattering time. The first part of the equation above is the intra-band contribution, and the second part contributes to the inter-band contribution.

Correspondingly, the dielectric constant of graphene layer can be expressed as:

(2) $$\varepsilon_{g}=1+j\frac{\sigma_{g}}{\omega \varepsilon_{0}\Delta }$$

where Δ is the graphene layer thickness, and *ε*_0_ is the permittivity of free space.

### 2.1 Graphene supported THz BIC metamaterials

Due to the relatively low energy of non-radiative THz wave, the intra-band transition plays an important role, and the permittivity of graphene layer indicates a negative real part, which demonstrates sensitive, broadband, and ultrafast Drude response. Furthermore, thanks to the high carrier concentrations of graphene laying in the range of 10^9^–10^12^ cm^2^, the plasmonic resonances are very strong in the THz and Mid-IR spectral regions, inducing intensive interactions between graphene and incident waves. If a uniform graphene layer is utilized, the tunable range of Fermi level is often small, typically less than 0.1 eV. However, if structural graphene ribbons are utilized to modulate the THz waves, the Fermi level can reach a large value about 1.0 eV to enhance the tunable properties. For previously reported MMs devices, suitable and optimized geometric parameters are often required to precisely manipulate the resonant frequency, amplitude, phase and polarization. The common resonators mainly include cut wire (CW) stripes, pairs of bars, split rings, crosses, and H-shaped resonators. Next, we provide a summary of the various tunable graphene stripe and split ring resonators supported tunable quasi-BIC resonances.

#### 2.1.1 Graphene stripe supported THz BIC metamaterials

As a typical subwavelength resonator, CW stripe enjoys the merits of simple structure and various design DoF to optimize the resonances for high *Q–factor*, which is widely investigated in the development of BIC MMs.

**Figure 3:**
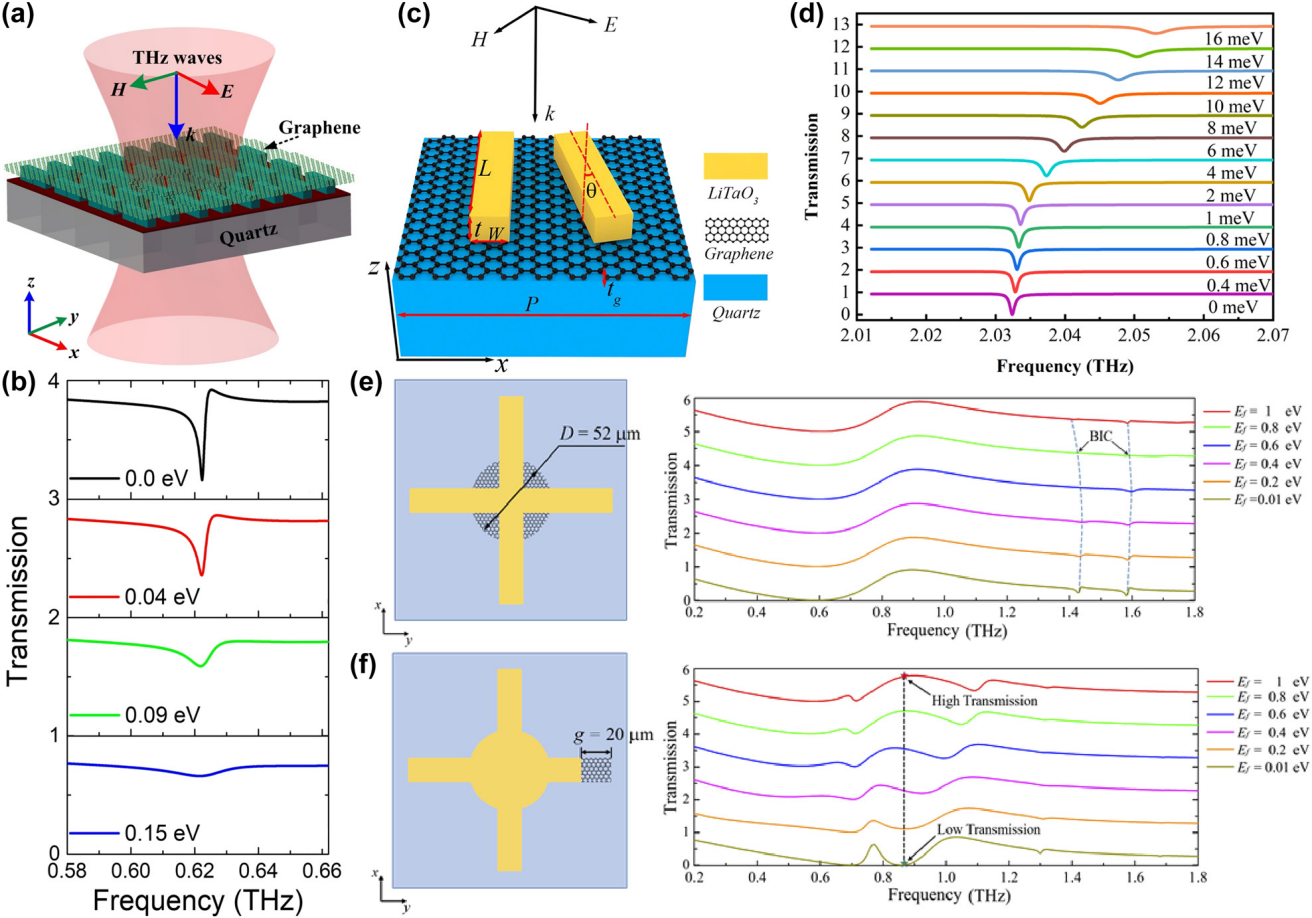
The graphene supported tunable BIC resonance with stripe resonators in the THz region. (a) and (b) Sketch and the transmission spectra of the proposed graphene-SrTiO_3_ metasurfaces [166]. Figures adopted with permission from Ref. [166] under License CC BY 4.0. (c) and (d) Schematic of the graphene-LiTaO_3_ hybrid MMs, and the influences of graphene Fermi levels on the transmission spectra [167]. Figures adopted with permission from Ref. [167]. Copyright 2023, Elsevier. (e) The graphene parameter-tuned BIC and the transmission spectra at different Fermi levels [168]. (f) The graphene symmetry-protected BIC and the transmission spectra at different Fermi levels [168]. Figures adopted with permission from Ref. [168] under License CC BY 4.0.

By integrating a uniform graphene layer with two pairs of SrTiO_3_ (STO) bars at different lengths, X. Chen and W. H. Fan proposed a graphene supported tunable MMs to excite BIC resonance [166], as given in Figure 3(a). As the length difference increases, the electric quadrupolar moment is excited, and the electric fields mainly constrain at the gap and ends of the stripes, the transmission curves indicate distinct Fano resonant dips, and the line width also become widen. The BIC resonance can be modulated individually by the graphene Fermi levels and STO temperatures. When the Fermi level is zero, it behaves just like a dielectric layer. If the Fermi level is 0.04 eV, the amplitude of transmission dip becomes shallow, while if the Fermi level increases to 0.09 eV, the transmission dip is very weak, which almost disappears when the Fermi level reaches more than 0.15 eV. In this case, as given in Figure 3(b), the transmission dip amplitude is modulated in the range of 0.1627–0.6608, and the amplitude of modulation depth (MoD) is about 50 %. When the temperature increases from 270 K to 390 K, the STO permittivity decreases, the resonant frequency is adjusted from 0.6108 THz to 0.6458 THz. The multiple BIC modes are also urgently needed to achieve flexible control of EM waves.

By inserting a uniform graphene layer between a pair of dielectric LiTaO_3_ bars and quartz substrate [167], as given in Figure 3(c), S. L. Ma et al. demonstrated a tunable THz modulator. An obvious transmission dip is observed owing to a quasi-BIC resonance with the variance of tilted angle of the right bar. As the rotation angle of the right bar decreases, the resonant dip becomes sharper and displays a red shift, on the condition that the rotation angle is less than 2°, the *Q–factor* is larger than 35000. For instance, if the rotation angles are 1°, 2°, 5°, and 20°, the according *Q–factor*s are 35455, 8870, 1426, and 85.2, respectively. The analysis of electromagnetic multipolar decomposition shows that the transmission dip stems from the EQ and MD moments. As Fermi level increases, the graphene permittivity increases intensively, the transmission dip indicates a blue shift, and becomes shallow and broad, which can be found in Figure 3(d). When the Fermi levels are 0, 4, 10, and 16 meV, the *Q–factor*s are 2438, 1258, 712.6 and 484, respectively. The according modulation depth is more than 97 %. As a THz gas sensor, on the condition that the Fermi level is 2 meV, and the rotation angle is 4°, the MoD is about 90 %, the sensitivity is 309.5 GHz/RIU. Based on a cross and disk compound metal resonators shown in Figure 3(e), in 2022 Y. F. Chen et al. proposed a hybrid THz BIC resonant structure [168] closely associated with the diameter of metal disk. When the diameter of metal disk is small, *e.g.* 32 μm, the dual quasi-BIC states are excited at the frequencies of 1.52 THz and 1.66 THz, and the interaction mainly constrains near the horizontal metal stripe. When the diameter increases to 52 μm, the resonant dip disappears owing to the fact that Friedrich–Wintgen condition is matched. However, when the metal disk diameter is 72 μm, most of the electric fields are confined around the disk, and the quasi-BIC resonance appears again. By replacing the metal disk with a uniform graphene layer given in Figure 3(e), many interesting phenomena are observed. If the Fermi level of graphene layer is small, *e.g.* 0.01 eV, its effect can be ignored. When the Fermi level increases to 0.8 eV, the graphene ribbon manifests much stronger electric fields interact with metal stripes strongly. Additionally, by replacing the right arm of metal cross stripe with graphene ribbons, as given in Figure 3(f), broad tunable BIC resonance is observed. As Fermi level increases, the low frequency quasi-BIC dip remains unchanged, while the second BIC resonance indicates an obvious blue shift, which forms a broadband high transmission peak. Since the complex conductivity of graphene increases significantly with Fermi level, the quasi-BIC transmission dip indicates an obvious blue-shift, *e.g.* at the operation frequency of 0.85 THz, the transmission varies from 1 % to 78 % when the Fermi level varies in the range of 0.01–1.0 eV. In addition, based on a high-resistivity Si-based stripe grating, M. Kim et al. proposed a tunable THz modulator, a double-layered graphene with a 5 nm gap was utilized to apply the bias voltage [169]. On the condition that the incident angle is 12.5° and the graphene Fermi levels are 0, 10, 20, and 50 meV, the *Q–factor*s are 20900, 2560, 935, and 0, respectively. The Fano resonant dip amplitudes are also very sensitive to Fermi level, *e.g.* the Fano resonant dip amplitude increases from 0.095 to 0.802 when Fermi level increases from 0 to 10 meV. If Fermi level increases to 90 meV, an amplitude MoD of nearly 100 % is achieved, and the insertion loss is 36 %.

**Figure 4:**
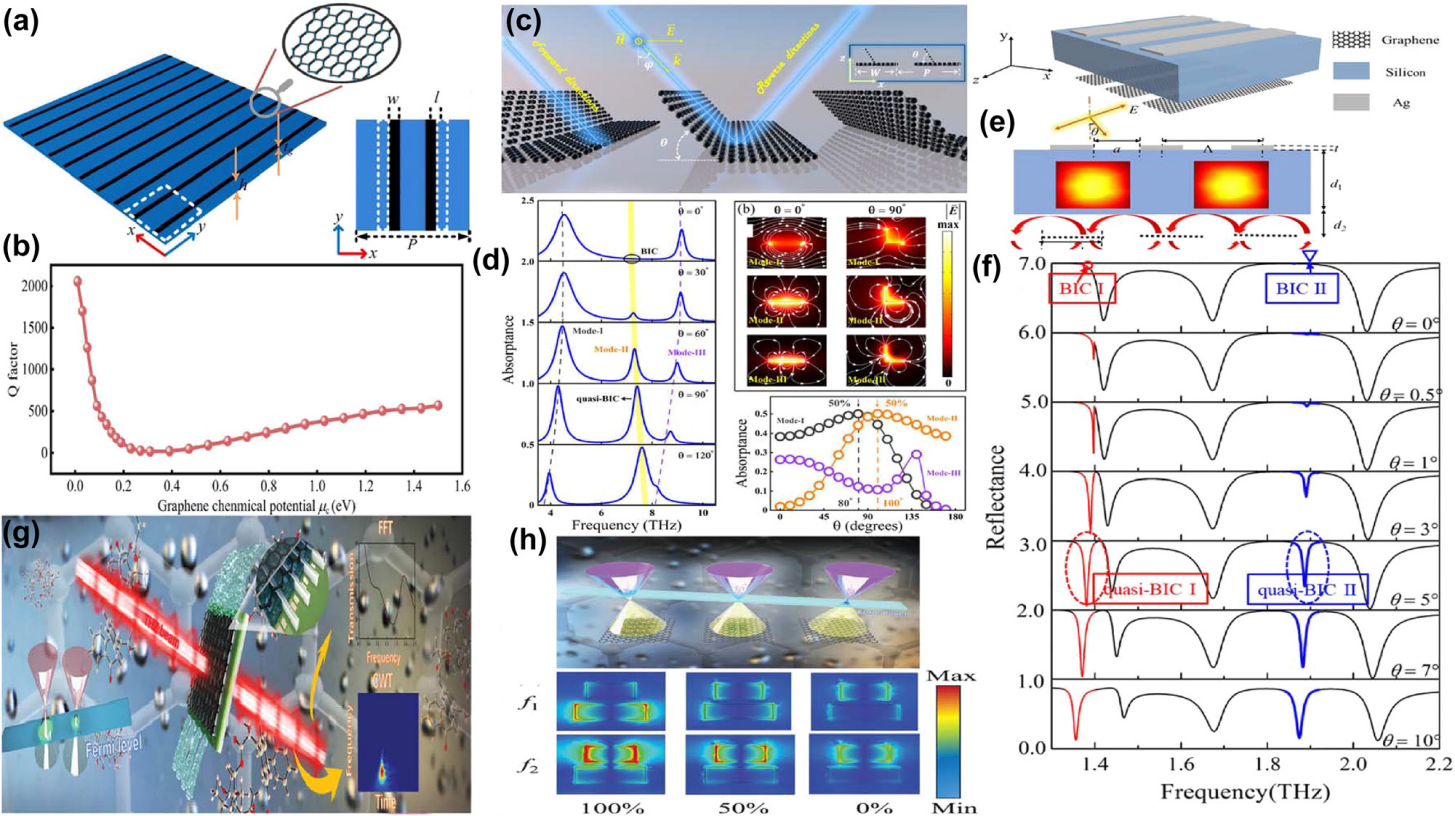
The graphene stripe patters supported tunable THz BIC resonance. (a) Scheme of a graphene meta-grating support quasi-BIC by introducing periodic perturbations [170]. (b) The *Q–factor* of the graphene meta-grating versus Fermi level. Figures adopted with permission from Ref. [170]. Copyright 2023, Elsevier. (c)-(d) The schematic diagram of an array of folded graphene ribbons, and the according optical absorption curves [171]. Figures adopted with permission from Ref. [171] under License CC BY 4.0. (e) The diagram of the graphene plasmonic waveguide, the excitation of guided-mode resonance and graphene localized plasmons mode [172]. (f) The reflection spectra for different incident angles to show the trends of the symmetry-protected BIC evolving to a quasi-BIC [172]. Figures adopted with permission from Ref. [172]. Copyright 2023, Royal Society of Chemistry. (g) Schematic diagrams of sensing based on the graphene supported quasi-BIC resonance biosensor [173]. (h) Schematic illustration and the simulated distributions of electric field on the *x-y* plane of the graphene quasi-BIC biosensor at ethanol concentrations of 100 %, 50 %, and 0 % [173]. Figures adopted with permission from Ref. [173]. Copyright 2024 Wiley‐VCH GmbH.

By depositing graphene micro-ribbon arrays on polyimide layer and introducing a displacement along the *x* direction, Y. Wang *et al. *proposed a tunable THz BIC resonator device [170], as given in Figure 4(a), which mainly results from the contributions of ED and EQ moments. When the Fermi level increases from 0 to 1.5 eV, the amplitude varies in the range of 0.076–0.821, the according MoD is about 75 %, and the *Q–factor* shifts in the range of 2000–500, which can be found in Figure 4(b). By using a free-standing folded graphene ribbon given in Figure 4(c), several perfect optical absorption modes are excited in the THz region [171], especially a dipole state and BIC mode causes efficient absorption, the *Q–factor* of the absorption curve is larger than 25. For the normal incidence, if the folding angles are 80° and 100°, for the dipolar state and BIC mode, 50 % absorptance is achieved, which can be found in Figure 4(d). If the folding angle further increases, the absorption of the dipolar mode increases gradually to a maximum value, and then decreases rapidly to near zero. However, for the BIC mode, the absorption enhances rapidly to a maximum value, and remains stable in wide angle range. As the Fermi level increases, the perfect optical absorption of dipolar mode indicates a gradual blue shift, while the quasi-BIC mode shows a rapid blue shift and then divided into three branches, resulting from the Fano coupling to two guided modes. As given in Figure 4(e), M. Li et al. proposed a S–P BIC resonance based on a dielectric Si layer with the thickness of 46 μm sandwiched between the upper Ag periodic gratings and the bottom graphene ribbons [172]. By altering the incident angle by introducing a small perturbation, two quasi-BIC states are observed. As the incident angle increases, the resonant dip of reflection curve becomes stronger. When the incident angle is 5°, two obvious Fano resonances are excited, and the *Q–factor*s reach more than 1335 and 280 if the incident angle is 0.1°, which can be found in Figure 4(f). By integrating a uniform graphene layer with arrays of asymmetric dimers [173], as given in Figure 4(g), C. C. Huang et al. proposed a convenient and highly sensitive THz liquid biosensor based on quasi-BIC resonance, resulting from the interference coupling between the electric and magnetic dipoles. When the length of the second rod varies from 84 μm to 44 μm, *e.g.* the asymmetric parameter increased from 0.05 to 0.31, the *Q–factor* decreases from 40 to 5, the transmission curve becomes stronger and broader. At the quasi-BIC resonance, the binding photon interacts with the analyte strongly and enters the excited state, which interacts easily with the electrons of graphene membrane. Furthermore, the graphene electron is also strongly affected by the non-covalent surface transfer doping interactions between metasurface and the analyte. When the Fermi level changes in the range of 0.02–0.18 eV, the transmission dip becomes weaker and broader, the resonant frequency indicates a red shift, and the according *Q–factor* reduces from 5.2 to 1.2. As given in Figure 4(h), this proposed THz graphene-metasurface sensor can discern the ethanol and N-methylpyrrolidone from 0 % to 100 % by detecting the variance of the quasi-BIC resonant intensity, the lowest detection concentration is about 0.21 pg mL^−1^.

#### 2.1.2 Graphene split ring supported THz BIC metamaterials

Split ring is another type of common resonators and provides many more choices for designing novel functional MM devices. As an artificial “magnetic atom,” the split-ring-resonator (SRR) is one of the most attractive optical resonators [174], [175], [176], which provides an excellent and flexible platform for constructing various MMs structures with complex coupling distributions. SRR was first used to design high–*Q* magnetic resonators in the 200–2,000 MHz range to replace the bulky conventional cavities and impractical solenoid coils [177]. According to Faraday’s electromagnetic induction law, the surface of the metal ring stimulates the induced current, whereas at the opening of the SRR, many charges accumulate to form an equivalent capacitance. Thus, an SRR can be regarded as an inductance and capacitance resonator (LCR) circuit with a resonant angular frequency [178], [179], [180]. Additionally, compared to conventional resonant elements, the coupling coefficients between SRRs are not only dependent on the separation distance, but are also related to the rotation angle of two neighboring resonators, which can be regarded as a new degree-of-freedom for the SRR. So far, many SRR-variant structures based on single-ring configurations have been studied theoretically and experimentally, including double-ring, side coupling, complementary, and multilayer configurations, especially the special spiral SRR. Considering the effective inductance of the spiral SRR is formed by a metal strip, when the magnetic field is perpendicular to the spiral SRR, the metal surface also induces a surface current [181], [182]. Because the charge distribution is uneven throughout the structure, a potential difference between the metal strips of different coils is formed, generating an equivalent capacitance. With those above-mentioned properties, split ring resonators are widely employed in the development of tunable quasi-BIC resonance nowadays.

**Figure 5:**
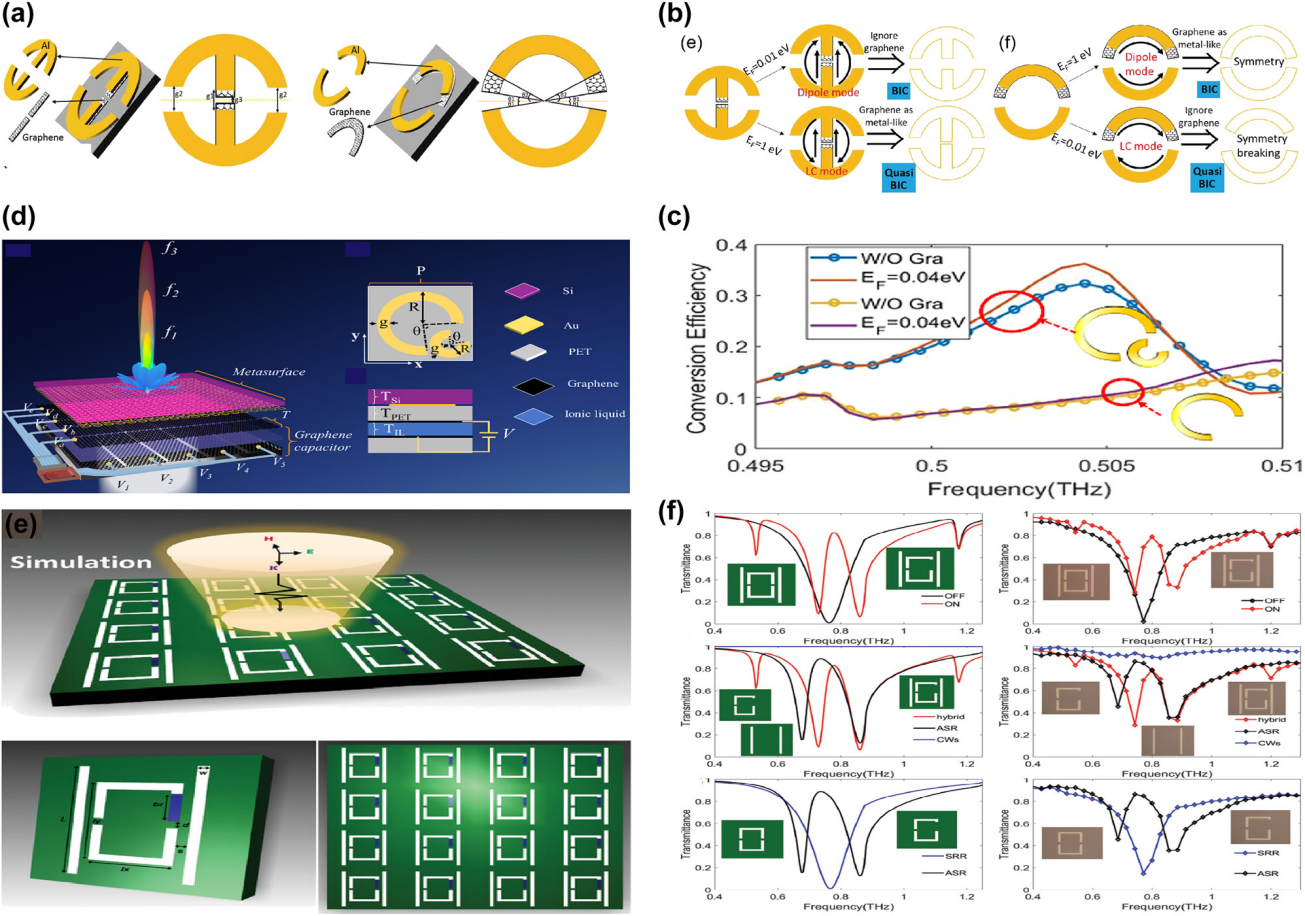
The graphene supported tunable BIC resonance with split ring resonators in the THz region. (a) Schematic for the parameter-tuned and symmetry-protected graphene-metal metasurface, *g*_1_ = 12 μm, *g*_2_ = 20 μm, the graphene split gap *g*_3_ = 1 μm; *θ*_1_ = 5°, *θ*_2_ = 35° [183]. (b) The schematic for parameter-tuned and symmetry-protected graphene-metal metasurfaces [183]. Figures adopted with permission from Ref. [183]. Copyright 2021, Elsevier. (c) Schematic of the reconfigurable THz wave modulator [184]. (d) Simulated transmission spectra, and conversion efficiency of the two individual C-shaped antennas, and transmission spectrum of the double C-shaped antenna without and with graphene at the frequency of 0.504 THz [184]. Figures adopted with permission from Ref. [184] under License CC BY 4.0. (e) The sketch of the graphene-Al hybrid asymmetric resonators [185]. (f) The simulation and experimental results for the graphene-metal ASR resonators at different bias voltage [185]. Figures adopted with permission from Ref. [185]. Copyright 2024 Wiley‐VCH GmbH.

As given in Figure 5(a), by integrating the metal split ring with graphene patterns, in 2021 J. T. Li *et al. *at Tianjin University proposed a tunable THz BIC resonance [183] and realized a facile switch between BIC and quasi-BIC via the variance of Fermi level. By adding a graphene rectangle stripe between the metal finger-like circular split ring and Si substrate, a tunable BIC resonance is observed in the frequency range of 0.20–0.48 THz. At small Fermi level, *e.g.* 0.01 eV, the graphene permittivity is low and its effect is negligible, so that the BIC resonance disappears. As Fermi level increases, a broad weak peak appears at the Fermi level of 0.1 eV. When the Fermi level increases to 0.4 eV, an obvious resonant peak is observed, and the peak value increases as well, which reaches about 0.70 if the Fermi level is 1.0 eV. Additionally, by inserting a monolayer graphene arc-shaped stripe below the metal circular split ring, similar dynamic control of BIC resonance is achieved, as given in Figure 5(b). When the bias voltage is large, 1.0 eV, the BIC status dominates, while for a low voltage, the electrons in graphene layer is small, the quasi-BIC resonance is excited. In 2023, as shown in Figure 5(c), based on a 5 × 5 antenna array independently modulated by switching the voltage applied on graphene ribbons-metallic antenna, B. Hu and Y. Zhang’s group proposed an electrically controlled active THz modulator. The flexible control of the phase profile and enhancement of conversion efficiency are achieved [184], with a *Q–factor* about 1500. For the single C-shaped antenna, the convention efficiency increases slightly with frequency, while for the double C-shaped resonators, due to the enhancement local states and the reinforcement of the interaction between THz waves and resonators, the conversion efficiency is improved significantly. As given in Figure 5(d), at the resonant frequency of 0.504 THz, the conversion efficiency for the single and double C-shaped are 9.5 % and 32.3 %, respectively. It should be noted the effects of graphene layer on the single C-shaped resonator can be neglected, and the graphene supported double C-shaped antenna increases significantly when the Fermi level is 0.04 eV. By introducing quasi-BIC resonance via a double C-shaped antenna, the efficiency is enhanced by 2.7–3.6 times when Fermi level changes in the range of 0.1–0.75 eV. Experimentally prepared by the laser cutting, the focal length of the proposed modulator varies from 14.3 mm to 22.6 mm. In 2024, as given in Figure 5(e), X. Y. Wang *et al*. proposed a programmable metasurface with robust high-order BIC resonance to achieve the secure Robotic brain [185], which consists of two Al cut wires and split-ring resonators, a graphene membrane is filled in the gap of one arm of metal SRR to break the asymmetry. At a low voltage, the graphene acts as a dielectric thin membrane, its effect can be negligible. Thus, the asymmetric Al resonators excite the obvious quasi-BIC resonant peaks at the frequencies of 0.53 THz, 0.73 THz, 0.86 THz when the polarization of incident wave is along the *x* direction, and a resonant valley is also shown at the frequency of 1.18 THz, as the red line given in Figure 5(f). However, if the Fermi level is large, the graphene layer inhibits strong metal properties, and the hybrid resonators act as symmetric metallic metasurface, two resonant valleys located at 0.76 and 1.18 THz appear. Thus, the free switching for controlling multiple frequency bands are achieved with the variance of bias voltage on the graphene patch. The proposed tunable high-order BIC robust resonances can be utilized to design security hardware, which offers broad prospects in the sensing and communication applications.

**Figure 6:**
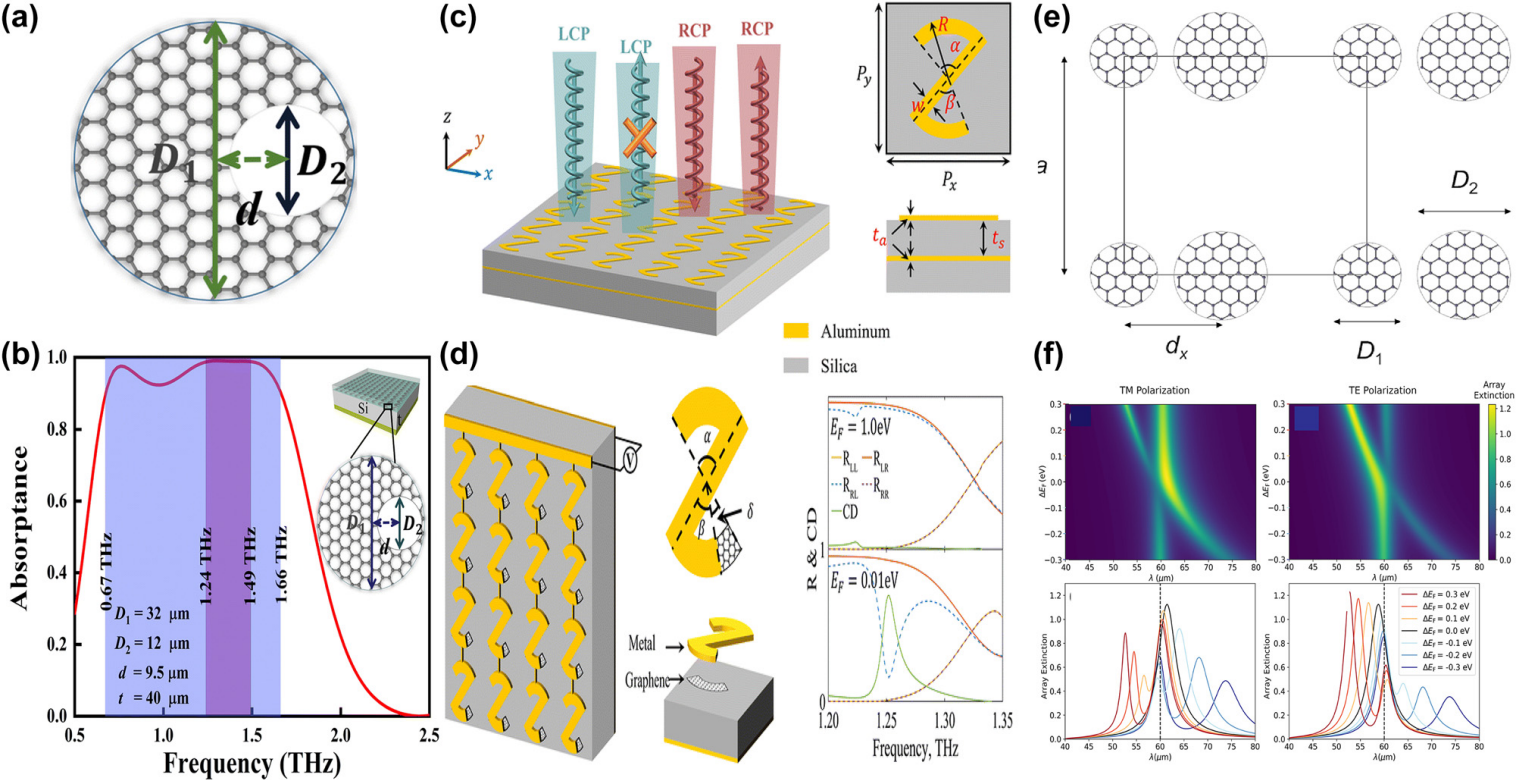
The graphene patters supported tunable THz BIC resonance with the split ring resonators. (a) The schematic diagram of the graphene nonconcentric ring with diameter D_1_, D_2_ and eccentricity *d* [186]. (b) Absorption spectrum obtained as using the optimized structural parameters of graphene non-concentric rings [186]. Figures adopted with permission from Ref. [186]. Copyright 2023, Royal Society of Chemistry. (c) Schematic of the proposed MIM chiral metamaterial consisting of an *S*-sharped meta-atom array [187]. (d) Schematic illumination of the chiral graphene–metal hybridized metamaterial, where *α* = 90°, *β* = 75°, and *δ* = 15°, and the according reflection and CD spectra of the graphene–metal hybridized metamaterial with E_F_ = 1.0 eV and 0.01 eV [187]. Figures adopted with permission from Ref. [187]. Copyright 2023, Royal Society of Chemistry. (e) Schematics of the square array of graphene microdisk dimers, the lattice parameter *a*, the separation between the disks *d*_x_, and their diameters D_1_ and D_2_ [188]. (f) Tunability of the array extinction for TM and TE polarizations at different Fermi levels [188]. Figures adopted with permission from Ref. [188] under License CC BY 4.0.

Based on a square lattice of graphene non-concentric rings, in 2023 K. Xu et al. proposed a tunable THz MMs absorber [186], which can be found in Figure 6(a). If the value of eccentricity is 4 μm, the lateral inversion symmetry is broken and a new transmission dip is observed at high frequency. As eccentricity increases, the high frequency transmission dip indicates a redshift, and the spectral line width becomes broadening. By introducing a metallic reflection layer, as given in Figure 6(b), a typical graphene MM absorber is proposed. Besides the dipolar resonance, the quadruplar mode at the high frequency is also excited, which extends the perfect absorption frequency to 1.47 THz. The resonant curve is also closely associated with Fermi level. At Fermi level of 0.1 eV, the absorption is about 50 %. As Fermi level increases, the absorption is enhanced, and achieves net perfect absorption on the condition that the Fermi level is 0.3 eV. The hollow graphene ribbon MM absorber manifests a broadband perfect absorption (>90 %) in the frequency range of 0.67–1.66 THz, and a fractional bandwidth is 85 %.

Based on an array of *S*-shaped metal resonators, as given in Figure 6(c), T. Ma et al. proposed a circular dichroism MM modulator [187], and the asymmetric parameter *δ* is defined as the difference of the angle between the upper arc (*α*) and bottom arc (*β*). The proposed metal *S*-shaped MMs modulators show only strong reflection resonance for the left-handed circularly polarized, reaching maximum circular dichroism (CD) on the condition that the asymmetric parameter is 15°. Additionally, by depositing the asymmetric *S*-shaped metal resonators with graphene arc ribbons, the tunable CD modulator is achieved, as given in Figure 6(d). When the Fermi level is low at 0.01 eV, its effect is neglected, the asymmetric hybrid structure indicates an obvious CD phenomenon. While if the Fermi level is large, *e.g.* 1.0 eV, the graphene ribbon indicates good plasmonic properties. The hybrid metal-graphene structure acts as a symmetric structure, and the CD phenomenon is very weak. If the asymmetric parameter is 15° (*α* = 90°, *β* = 75°), when the Fermi level changes in the range of 0.01 eV and 1.0 eV, the peak value of CD is shifted from 0.693 to 0.008. As given in Figure 6(e) [188], J. L. Pura et al. proposed a tunable THz quasi-BIC resonance by utilizing periodic arrays of asymmetric graphene dimers. For the transverse electric (TE) mode, if the diameter of graphene disk 2 is large, there exists an almost linear variation of the resonant wavelength, while the TM mode indicates the opposite behavior, as given in Figure 6(f). For the identical graphene dimers, if the Fermi levels are different, quasi-BIC mode is also exited. For example, if the Fermi level of graphene disk 2 is smaller (larger) than that of disk 1, with the difference of Fermi level between two graphene disks increases (decreases), the resonant curve indicates an obvious blue shift (red shift) and becomes sharper (broader). As the lattice parameter increases, for the TM mode, the value of full width at half maxima (FWHM) reduces, resulting into the enhancement of *Q–factor*.

### 2.2 Graphene supported Mid-IR BIC metamaterials

**Figure 7:**
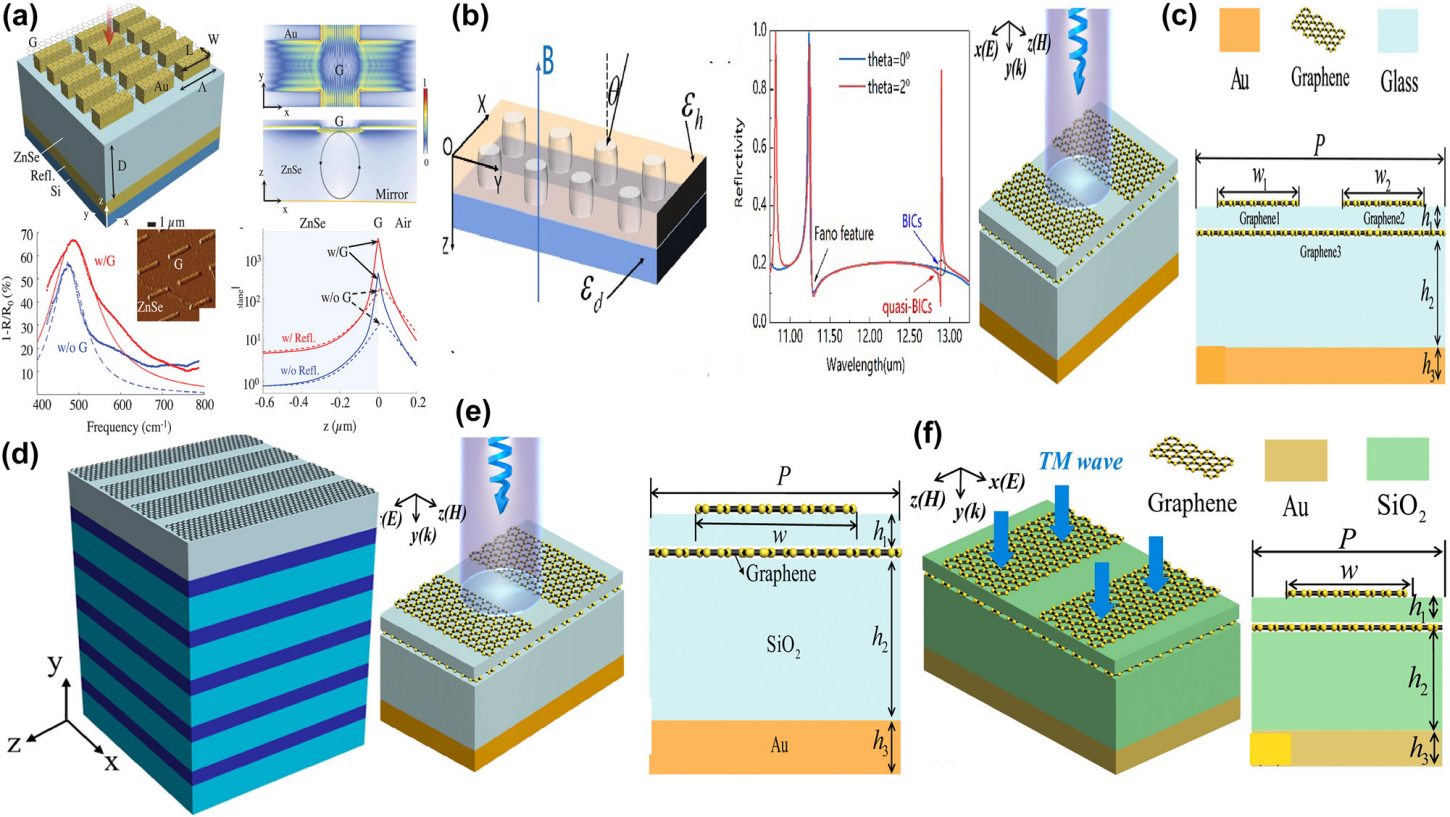
The graphene stripe patters supported tunable BIC resonance in the Mid-IR spectral region. (a) Graphene modified hybrid plasmon–photonic system [189]. Figure adopted with permission from Ref. [189]. Copyright 2024 Wiley‐VCH GmbH. (b) Diagram of the photonic crystal structure and the reflection coefficient at 0° and 2° incidence angles for the symmetry-protected BIC and sharp Fano resonance [190]. Figures adopted with permission from Ref. [190] under License CC BY 4.0. (c) The proposed diagram composed of asymmetric Fabry–Perot cavities [191]. Figure adopted with permission from Ref. [191]. Copyright 2022, Royal Society of Chemistry. (d) The proposed graphene-based all-dielectric absorber [192]. Figures adopted with permission from Ref. [192]. Copyright 2022, Elsevier. (e) 3D schematic diagram and 2D side view of the proposed graphene gratings and graphene sheets [193]. Figures adopted with permission from Ref. [193] under License CC BY 4.0. (f) Schematic diagram and side view of the proposed graphene F–W BIC-based metasurface [194]. Figure adopted with permission from Ref. [194]. Copyright 2023, Elsevier.

By integrating a uniform graphene layer with a typical metal–dielectric–metal MM absorber, *i.e.* micrometer-sized Au stripe-dielectric ZnSe spacer layer-Au reflection layer, T. Kananen et al. proposed a tunable graphene absorber enhanced by quasi-BIC resonances [189], as given in Figure 7(a). With the assistance of graphene membrane, the in-plane optical field intensity shows over an order of magnitude enhancement. The dielectric ZnSe spacer layer determines the coupling between in-plane plasmonic mode and the out-of-plane cavity mode. With the addition of a graphene layer, the absorption peak shifts toward the shorter wavelength and enhances the absorption. With the practical lossy rates and suitable Fermi level, when the quasi-BIC resonance is emerged and meets the associated critical coupling condition, the absorption reaches more than 90 % from the simulation expectation, and experimentally achieves more than 68 %, much larger than the results without graphene layer, only about 50 %.

In 2019, by utilizing the photonic crystal slab-graphene-slab structure given in Figure 7(b), G. Y. Chen et al. proposed strong THz magneto-optical phenomena with the quasi-BIC and Fano resonances [190]. For the normal incident, only Fano resonance at the wavelength of 11.25 μm is excited, while for the oblique incident wave, *e.g.* incident angle is 2°, the quasi-BIC with a high *Q–factor* is excited at the wavelength of 12.95°. Owing to the relatively smaller energy of Mid-IR spectral region, the effect of the intrinsic graphene layer on the resonant curves is neglected in the Mid-IR spectral region. However, the magnetic CD is affected by the graphene Fermi level significantly. Compared with the Fano resonances, the magneto-optical modulation thanks to the BIC resonance is more sensitive to Fermi level and indicates much narrower curves. Composed of upper asymmetric graphene stripes and bottom graphene nano-ribbon, as given in Figure 7(c), in 2022 E. D. Gao et al. proposed a dual-band ultra-high absorber to generate a symmetry-protected quasi-BIC and FP resonant mode [191], which reaches an ultra-high absorption of 99.5 % and 92 %, respectively. The proposed double layers graphene absorber is sustained a relatively wide range of incident and polarization angles, *e.g.* even if the incident angle increases to 60°, the absorption of quasi-BIC is still larger than 88.7 %. By depositing periodic graphene nano-ribbons on CaF_2_-1D photonic crystal (with at least 5 pairs of alternative Si–SiO_2_ multilayer, acting as a nearly-perfect reflection mirror) structure, M. Li et al. proposed a quasi-BIC MMs absorber [192] given in Figure 7(d). With suitable thickness of the insertion CaF_2_ layer, the quasi-BIC resonance is excited. As the thickness of spacer layer increases, the interaction between the graphene ribbons and the incident wave becomes weakened, and the intrinsic absorption is reduced, the *Q–factors* of the dual reflection peaks reach more than 450 and 600 in the Mid-IR spectral region, respectively. Additionally, as Fermi level increases, the graphene ribbons generate strong local fields and excited higher-order modes, the reflection resonant frequency manifests a blue shift. Additionally, the *Q–factor* increases with carrier mobility, multi-band absorption resonant curves are observed, and the optimized absorption peak value reaches more than 97 %. The absorption resonant wavelength of the proposed graphene-photonic crystal structure is insensitive to the incident angle. While the absorption intensity decreases as incident angle increases, the first-order and second-order reflection are larger than 70 % if the incident angle is smaller than 30°. As given in Figure 7(e), based on graphene gratings and uniform graphene sheet separated with a thin SiO_2_ layer with a thickness of 12 nm [193], they also demonstrate a penta-band ultrahigh absorption by utilizing the excitation of F–W BIC resonance, which stems from the destructive interference between Fabry–Perot resonance and the guided mode on the coherent phase-matching condition. Furthermore, the BIC resonance can be dynamically adjusted by changing the Fermi level of graphene gratings in the range of 1.0–0.5 eV, the tunable frequency range lies between 34.88 and 38.8 THz while maintaining the perfect absorption. In 2023, as given in Figure 7(f), E. D. Gao et al. proposed a dynamical near-field display by utilizing the F–W BIC resonance [194], resulting from the destructive interference between guided mode and F–P resonance modes in dual-layer graphene metasurface. When the graphene Fermi level is 1.0 eV, the guided mode is generated by the phase-matching between the incident light and propagation mode in the SiO_2_ plate, while the F–P mode results from the impedance-mismatch between the bottom uniform graphene layer 2 and the section without covering by the upper graphene ribbons 1. BIC resonance is excited at the frequency of 44 THz when the guided and F–P modes are strongly coupled.

**Figure 8:**
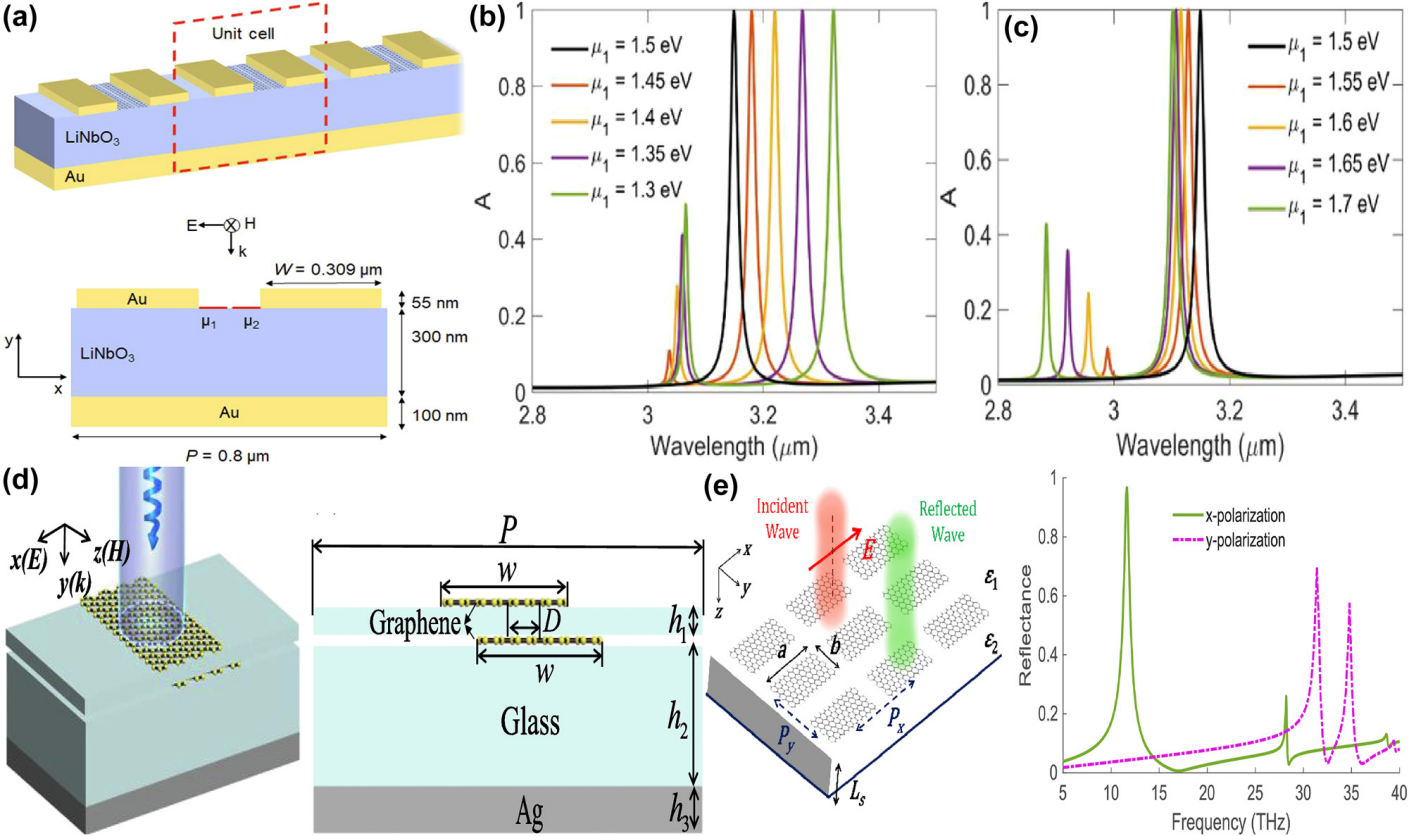
The graphene stripe patters supported tunable Mid-IR BIC resonance. (a) 3D schematic and unit cell of the graphene nanoribbons periodic device [195]. (b) Absorption spectrum obtained when μ_1_≠μ_2_ and μ_2_ = 1.5 eV [195]. (c) Absorption spectrums obtained when μ_1_ > μ_2_ and μ_2_ = 1.5 eV [195]. Figures adopted with permission from Ref. [195] under License CC BY 4.0. (d) The sketch for the upper and lower asymmetric graphene ribbons [196]. Figures adopted with permission from Ref. [196]. Copyright 2024, Elsevier. (e) Schematic view of the reflector consisting of rectangular graphene patches and the reflection curve for different polarization directions [197]. Figures adopted with permission from Ref. [197] under License CC BY 4.0.

Based on the periodic subwavelength resonators-dielectric LiNbO_3_ spacer layer-Au substrate structure, S. Guadagnini et al. proposed a tunable Mid-IR MMs absorber by utilizing the reversible symmetry breaking BIC resonance [195]. The hybrid resonators consist of two gold grating teeth and double graphene ribbons, the gap between the graphene nanoribbons is 5 nm, which can be found in Figure 8(a). For the structural graphene-Au hybrid resonator, if the Fermi levels for the left and right graphene ribbons are both 1.5 eV, a single perfect absorption peak is excited at the wavelength of 3.1 μm. On the condition that the Fermi level of right graphene ribbon is fixed at 1.5 eV, as the Fermi level of left ribbon increased from 1.3 eV to 1.5 eV, as given in Figure 8(b), the original longer wavelength peak indicates a blue shift, and the lower wavelength peak near 3.06 μm appears and shifts toward shorter wavelength, the amplitude decreases as well. Furthermore, as given in Figure 8(c), if the Fermi level of left graphene ribbon increases from 1.5 eV to 1.7 eV, the resonant peaks indicate blue shifts, the absorption peak at short wavelength enhances significantly. Additionally, as the length of GNR increases or Fermi level decreases, the on/off switching wavelength shifts longer wavelength. In 2024, a tunable MMs absorber based on double layers of graphene ribbons separated by a thin dielectric layer is also proposed [196], which is found in Figure 8(d).

By shifting the distance between the upper and bottom graphene ribbons to introduce an asymmetric parameter, a quasi-BIC resonance is excited. With a tiny displacement of 5 nm, two tunable plasmon-induced absorption peaks with the values of 99 % and 99.4 % are excited, resulting from the coupling between the bright modes. As the Fermi level of graphene ribbon reduces, the absorption peak manifests a red-shift, the modulation range reaches more than 4.95 THz. Additionally, when the polarization angle changes from zero to 90°, the absorption decreases from perfect absorption to zero, the MoD being about 100 %, and the insertion loss is only about 0.040 dB. As the carrier mobility increases, the resonant absorption peak indicates a red shift, and the group delay reaches more than 10.3 ps. Based on rectangular graphene patterns in the THz and Mid-IR spectral regions, M. Danaeifar proposed a tunable BIC resonance by changing structural parameters, Fermi level, or rotating the graphene ribbon [197]. As given in Figure 8(e), by reducing the length of upper graphene ribbon, a sharp reflection dip is observed with the *Q–factor* of more than 490, which results from the low dissipation of graphene layer. Similar reflection BIC dip is be excited by rotating the upper graphene ribbon. As the rotation angle increases, the resonant strength becomes stronger, the *Q–factor* decreases. For example, on the condition that the rotation angles are 5° and 20°, the amplitudes of reflection dips are 0.01 and 0.85, and the according *Q–factor*s are 645 and 45, respectively. Similarly, the variance of the Fermi level of the upper graphene ribbon is also an important means to achieve BIC resonance. If the difference of Fermi level between graphene ribbons is 2 meV, the small reflection dip appears with a high *Q–factor* of 756. As Fermi level increases, the BIC resonance strength become stronger with a lower *Q–factor* owing to the larger dissipation.

### 2.3 Graphene supported Near-IR BIC metamaterials

In the near-IR spectral region, the energy of incident wave is relatively large, the contribution of inter-band transition should be taken into account. When the Fermi level is low, *e.g. E*_f_ = 0.1 eV, ℏω>2μ_c_, the energy of incident wave is large enough to excite the electron from valence band to the conduction band, and the inter-band transition plays an important role. In this case, the graphene layer manifests a dielectric layer with a positive real part of permittivity, which can be modulated in a wide range by applying a bias voltage. As Fermi level increases, the number of final states allowed for an inter-band transition contribution reduction drastically. On condition that ℏω≈2μ_c_, the inter-band transition is almost blocked, resulting into the reduction of graphene permittivity as well. In this case, the value of the graphene permittivity is very small and indicates the ENZ phenomenon. As Fermi level increases further, the intra-band transition dominates, the graphene layer manifests plasmonic properties with a negative real part of permittivity. The graphene based quasi-BIC resonances in the near-IR spectral region are mainly associated with above mentioned tunable properties. Based on the drastic variance of graphene permittivity in the near-IR spectral region, much research is carried out to investigate the graphene supported tunable BIC MMs.

#### 2.3.1 Graphene stripe supported Near-IR BIC metamaterials

**Figure 9:**
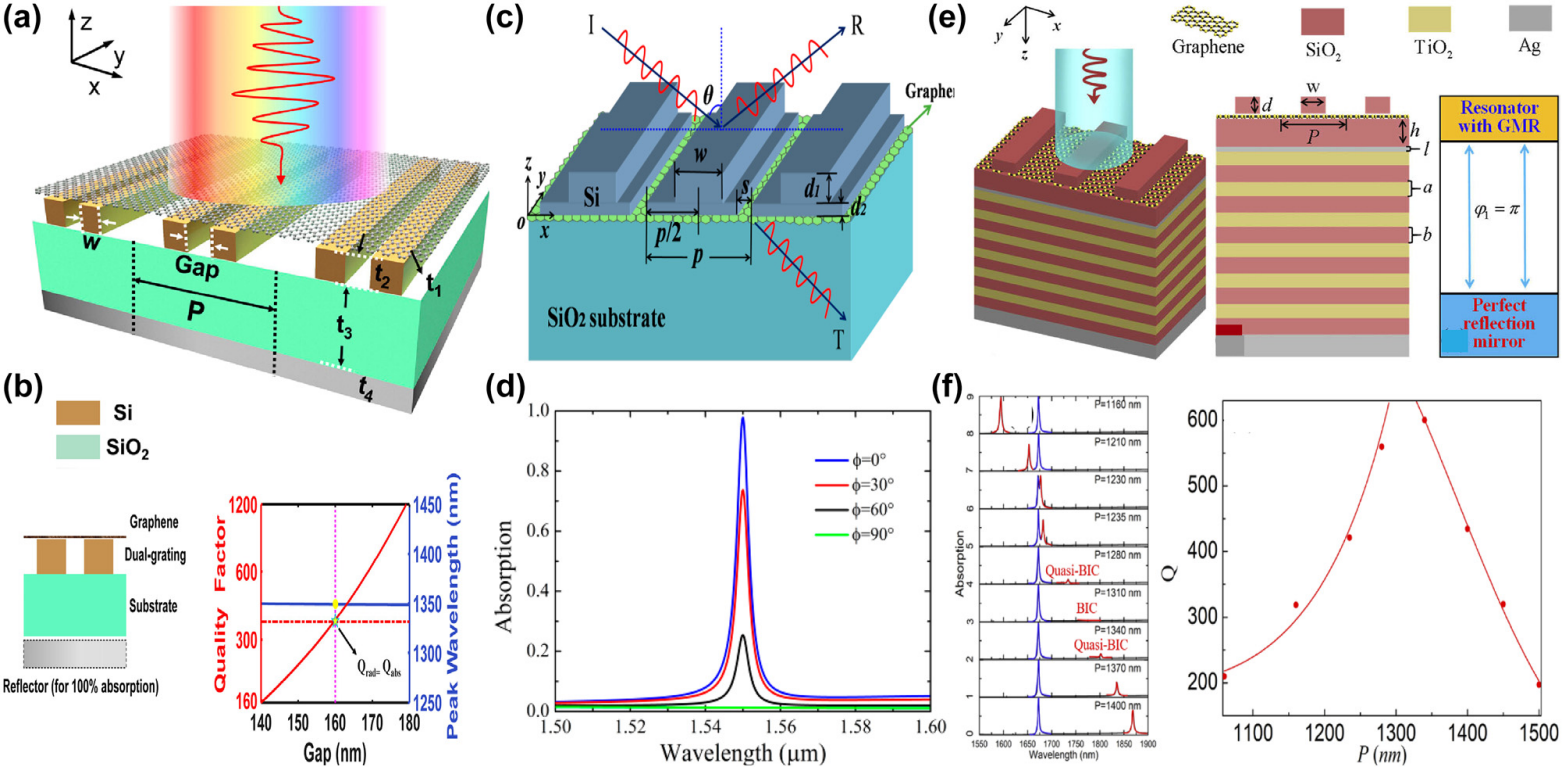
The uniform graphene membrane supported tunable BIC resonance with dielectric stripes resonators in the near-IR spectral region. (a) Schematic of the designed graphene metasurface with an Ag layer as a reflector [198]. (b) Cross section of the compound grating structure, and the according *Q*_abs_ and *Q*_rad_ versus gap distance of the dual-grating period [198]. Figures adopted with permission from Ref. [198] Copyright 2023 American Chemical Society. (c) Schematic diagrams of a Si-based slab with a monolayer graphene at the incident angle of *θ* [200]. (d) Absorption spectra as a function of the polarization angle [200]. Figures adopted with permission from Ref. [200] under License CC BY 4.0. (e) 3D schematic diagram of a grating-graphene-Bragg mirror system [201]. (f) The F–P BIC resonance and *Q–factor*s at different grating pitches [201]. Figures adopted with permission from Ref. [201] under License CC BY 4.0.

As a highly tunable dielectric medium, a uniform graphene membrane is often utilized in the near-IR spectral region. For instance, by depositing a monolayer of graphene on the top of Si grating structures consisting of a pair of identical nanowires, in 2023 R. Jin et al. experimentally proposed a tunable graphene MMs absorbers structures [198], as given in Figure 9(a). The *Q–factor* of the resonant curve can be manipulated by changing the gap of the grating. The BIC mode is regarded as a toroidal dipole confirmed by the multipolar decomposition method. The absorption peak wavelength remains stable upon the variation of the gap distance owning to the total volume of the dielectric materials keep unchanged. Since the radiation losses of the quasi-BIC resonance and non-radiative losses of the graphene membrane can be modulated conveniently by changing the gap of dimer nanowires or graphene Fermi level, respectively, the critical coupling condition is easily realized for this graphene-Si dimer hybrid structure. The experiments also confirm that the perfect graphene absorber achieve a maximum absorption about 96 %, and the *Q–factor* reaches more than 350, as given in Figure 9(b).

In 2021, based on a simple Si-based photonic crystal slab given in Figure 9(c), T. Sang et al. demonstrated an efficient graphene absorber, a BIC resonance was excited to achieve high confinement and near-field enhancement by introducing a small slit width about tens of nanometers, which results into near-perfect absorption and very sharp transmission dips [199], [200]. With the proper structural parameters, the absorption reaches about 98 % at the wavelength of 1.55 μm. The proposed graphene-based Si photonic crystal structure is also sensitive to the incident angle. The absorption bandwidth becomes broaden at larger incident angle, and the resonant peak indicates a blue shift. Additionally, the polarization angle also affects the peak values of the transmission resonant curves significantly, which can be found in Figure 9(d), on the condition that the polarization angles are 0°, 30°, 60°, and 90°, the peak values are 97.8 %, 76 %, 25 %, and 1.2 %, respectively. By utilizing a SiO_2_ grating-monolayer graphene-spacer layer-Bragg mirror system given in Figure 9(e), E. D. Gao et al. proposed an optical BIC mode in the near-IR spectral region [201], which results from the contribution of guide-mode resonance and Tamm plasmon polariton modes. The Bragg mirrors are composed of an Ag substrate and 1D photonic crystal with six pairs of alternately stacked SiO_2_ and TiO_2_ layers. This proposed graphene-Bragg structure acts as an optical switcher by the altering of the TE and TM waves. On the condition that the phase difference between the guide mode resonance and Bragg mirror meet the transverse resonance principle, the F–P BIC resonance is observed, which can be generated by the static (the grating pitch period) or the dynamic parameter (incident angle). As the incident angle increases, the guided mode resonance manifests a red-shift and gradually disappears. For instance, a BIC mode is formed if the indecent angle is 9.5°, the *Q–factor* is more than 600, which can be found in Figure 9(f).

**Figure 10:**
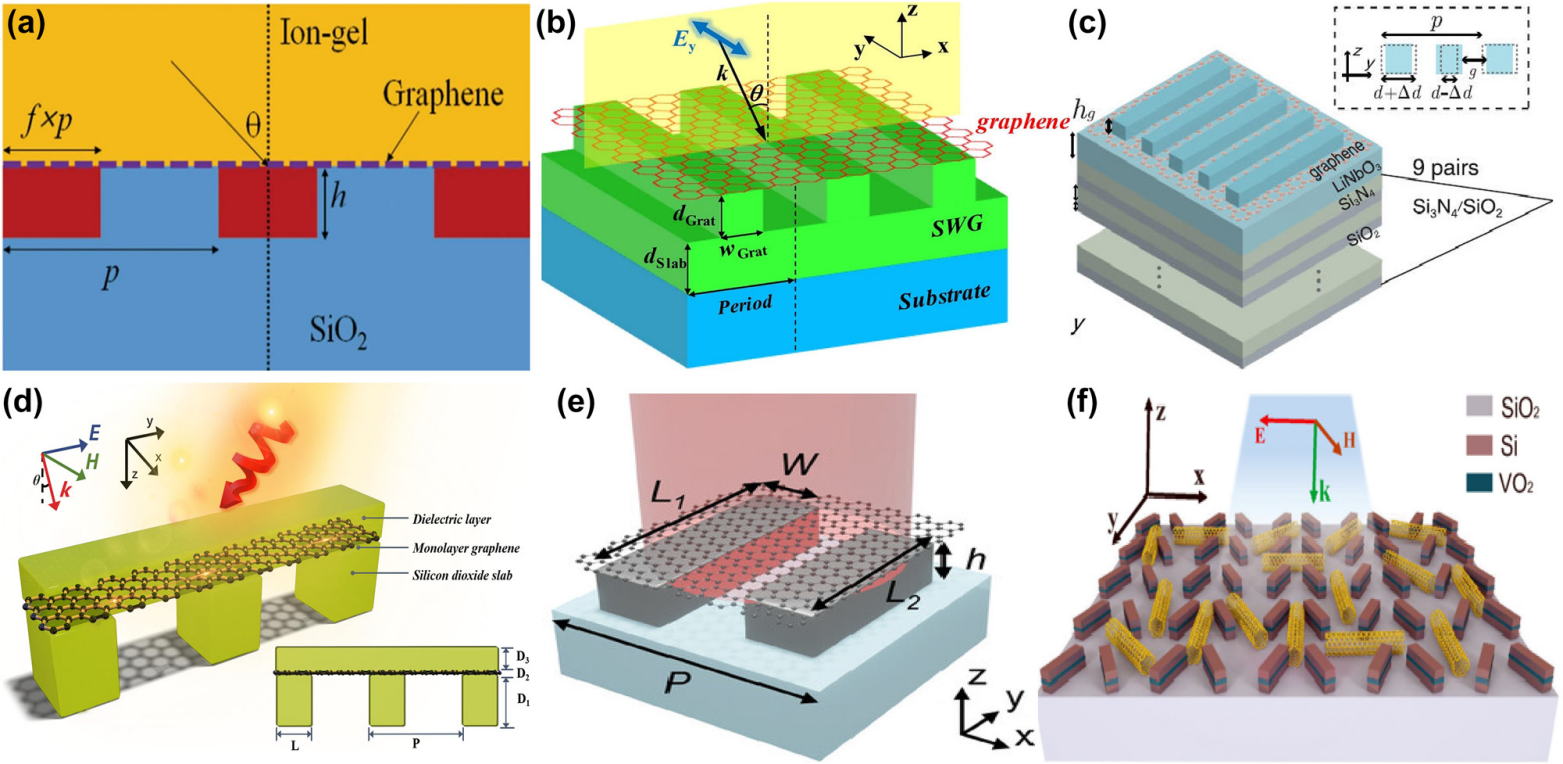
The uniform graphene supported tunable near-IR BIC resonance with stripes resonators. (a) Schematic of the proposed perfect absorber consisting of monolayer graphene placed on a slab-waveguide grating [202]. Figures adopted with permission from Ref. [202] under License CC BY 4.0. (b) The sketch of the proposed optical modulators composed of ion gel and single-layer graphene [203]. Figures adopted with permission from Ref. [203] under License CC BY 4.0. (c) Schematic of the quasi-BIC structure integrated with graphene. The quasi-BIC structure is constructed by 1D symmetry-broken dimerized LiNbO_3_ lattice structure deposited atop a multilayer dielectric Bragg reflector with nine pairs of Si_3_N_4_/SiO_2_ [204]. Adopted with permission from Ref. [204]. Copyright 2023, APS. (d) 3D Schematic illustration of the graphene grating sensor, comprising a 1D silicon dioxide slab, monolayer graphene, and dielectric layer [205]. Figures adopted with permission from Ref. [205]. Copyright 2025, Elsevier. (e) Schematic of the unit cell of the hybrid graphene-silicon metasurface, graphene is placed on top of an array of silicon nano-bars fabricated on a silica substrate [206]. Figures adopted with permission from Ref. [206]. Copyright 2025 Wiley‐VCH GmbH. (f) 3D schematic of the coupling system: Si-VO_2_ metasurface overlaid by a layer of random (6,5) SWCNTs [207]. Figures adopted with permission from Ref. [207]. Copyright 2024, APS.

By depositing a uniform graphene on a Si slab-waveguide grating stacked on a SiO_2_ substrate, as given in Figure 10(a), S. Lee et al. at Ajou University, proposed a tunable perfect absorber in the near-IR spectral region [202], which supports the quasi-BIC and guided-mode resonances, and the leakage rate of the former can be controlled by the incident wave angle. With suitable structural parameter and incident angle, a strong absorption peak owing to the quasi-BIC resonance is observed if the incident angle varies in the range of 2–6°, especially the perfect absorption is achieved at the wavelength of 1.5472 μm on the condition that the incident angle is 3.72°, the *Q–factor* is larger than 700. Furthermore, as Fermi level increases, the critical wavelength decreases slightly, and the incident angle decreases. For instance, at the Fermi levels of 0.0, 0.4 eV, and 0.5 eV, the critical angles are 3.72°, 2.63°, and 0.57°, respectively. They also proposed a tunable BIC absorber by depositing a uniform single layer graphene and double-layer graphene on the periodic Si bricks embedded in the dielectric surroundings of SiO_2_ [203], which can be found in Figure 10(b). At an incident angle of 12°, when the Fermi level increases from 0.4 eV to 0.7 eV, the transmission dip becomes stronger, the *Q–factor* is about 2 × 10^5^, the resonant dip manifests a blue shift slightly, the modulation depth reaches more than 99.72 %, and the insertion loss is 0.0034.

Based on a symmetry-broken LiNbO_3_ lattice structure deposited on a multilayer dielectric Bragg reflector, J. Wang et al. proposed a graphene supported tunable giant bandwidth perfect absorbers [204]. As given in Figure 10(c), consisted of nine pairs of Si_3_N_4_/SiO_2_, a dielectric Bragg reflector is utilized as a fully reflective mirror to achieve the perfect absorption. The uniform graphene membrane is inserted into between dielectric grating and the unetched LiNbO_3_ film. The width perturbation δ_d_ is introduced to break the in-plane inversion symmetry, the asymmetric parameter is defined as *α* = δ_d_/d, the widths of the two stripes are d+δ_d_ and d-δ_d_, respectively. As the asymmetric parameter increases, the reflection dip becomes broader and shallower, the resonant frequency indicates a blue shift. The bandwidths of the absorption curves can be tailored by the graphene layer number or the distance of graphene layer away from the unetched LiNbO_3_. In the near-IR spectral region, the graphene membrane is regarded as a lossy dielectric layer, thus the non-radiation dissipation increases with the layer number of graphene, the bandwidth of the absorption curve increases more than 11 times. Additionally, the non-radiation loss decreases significantly with the increase of the distance of graphene membrane away from the LiNbO_3_. For example, if the distances are 0, 220, 350, and 500 nm, the asymmetric parameters to match the critical coupling condition are 0.175, 0.08, 0.035, and 0.012, respectively. In addition, in 2025 by inserting a uniform graphene layer between a dielectric layer and 1D photonic crystal gratings, Y. T. Yi et al. proposed a tunable ultra-high *Q–factor* BIC resonance dominated by the coherent perfect reflection principle [205], which can be found in Figure 10(d). Multiple MD quasi-BICs simultaneously are excited, six asymmetric Fano dips of 99.69 %, 99.90 %, 99.86 %, 99.99 %, 99.60 %, and 99.79 % were observed simultaneously. The sharp transmission dip indicates a large *Q–factor* of 159726, and the FOM reaches more than 10894, about one or two orders of magnitude larger than the reported results. By depositing a uniform graphene layer on the asymmetric Si dimer, as given in Figure 10(e), Z. Q. Cai et al. proposed an electrical tunable quasi-BIC resonance [206]. As the asymmetric parameter *α* increases, the *Q–factor* decreases, *e.g.* if the *α* values are 0.2, 0.3, and 0.4, the *Q–factor*s are 90.5, 53.0, and 32.5, respectively. Based on the coupled mode theory, under the critical coupling condition, the absorption reaches the maximum value of 50 %. Thus, for the large value of asymmetric parameters and dissipations, more layers of graphene are required to match the critical condition. With the ion gel method, when the bias voltage changes in the range of −2–2 V, the graphene Fermi level varies in the range of 0.1–0.5 eV, the transmission curve indicates a blue shift and become deeper, the experimental transmittance decreases from 76.8 % to 54.6 %, and the according amplitude MoD is 28.9 %.

Additionally, the carbon tube can also be utilized to modulate the quasi-BIC resonance of dielectric metasurface [207], which can be found in Figure 10(f). With a pair of tilted Si-VO_2_ hybrid stripes, P. Xie et al. proposed a highly compact all-dielectric metasurface, together with the SWCNT to active modulating the exciton-quasi BIC coupling. The dielectric properties of the phase change materials VO_2_ varies significantly with temperature and affects the SWCNT based exciton-polaritons effectively. Due to the phase change of VO_2_, the temperature-dependent permittivity is quite different. At the temperatures of 30 °C and 80 °C, VO_2_ acts as a low lossy dielectric layer and high dissipation metal layer, respectively. As the asymmetric parameter increases from zero to 30°, *i.e.* the tilted angle of the dimer, the *Q–factor* decreases from 13.8 × 10^3^ and 6.8 × 10^3^ when the temperatures of VO_2_ layer are at 30° and 80°, respectively. From the analysis of the multipole expansion method, the quasi-BIC resonance mainly results from the magnetic dipole and the electric quadrupole. As the concentration of SWCNT increases, the oscillator strength is enlarged. The Rabi splitting energy increases linearly with the sqrt root of the SWCNT concentration, and the slope at 30 °C is larger than that of 80 °C. The strong coupling can be achieved with a minimum concentration of 0.11 wt% and 0.41 wt% for the case of 30 °C and 80 °C, respectively.

**Figure 11:**
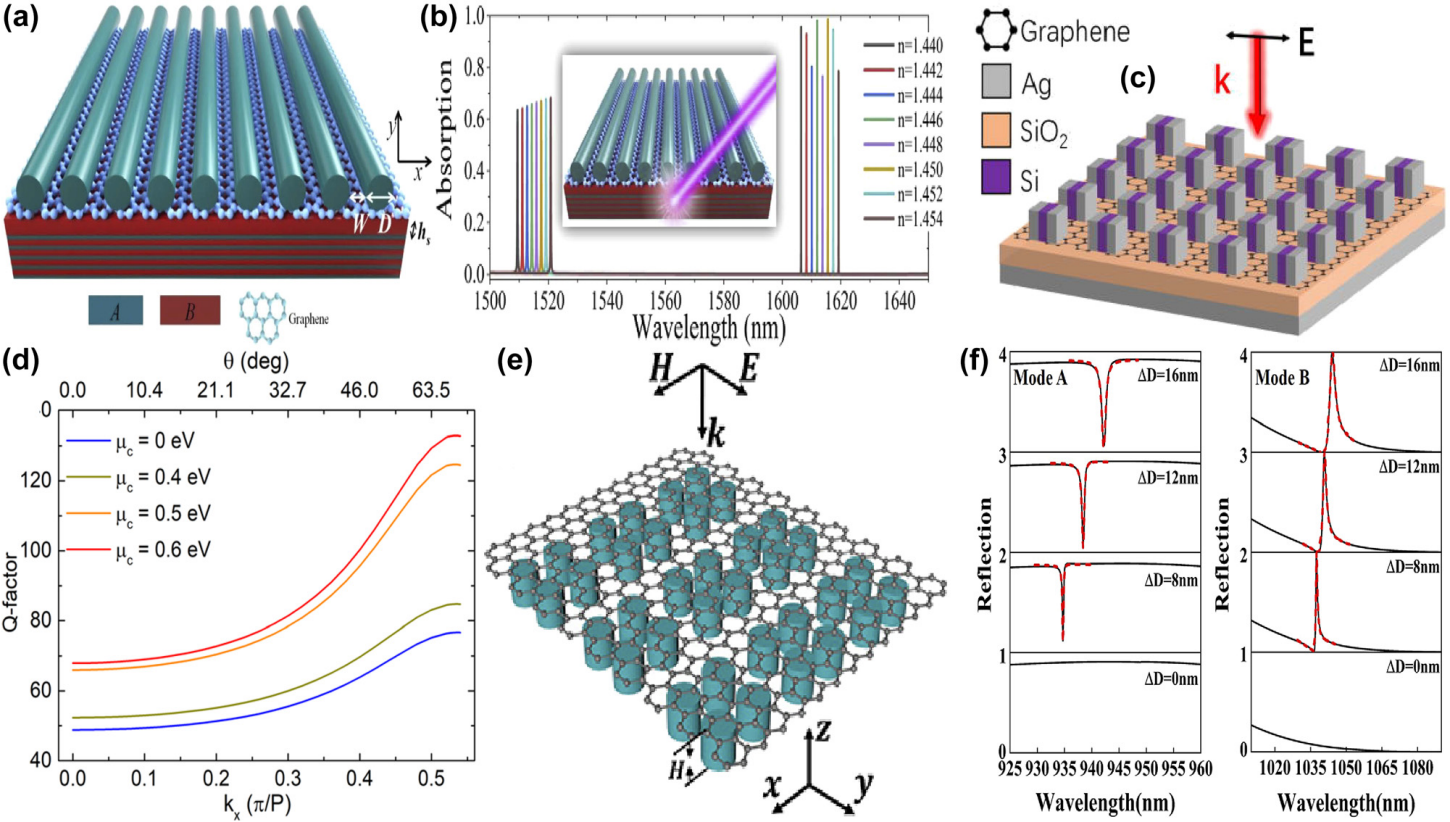
The uniform graphene supported tunable absorbers with BIC resonance in the near-IR spectral region. (a) Schematic of an ultra-high *Q–factor* graphene supported perfect absorber with BIC resonance [208]. (b) The absorption responses for the absorber under different n_a_ [208]. Figures adopted with permission from Ref. [208] under License CC BY 4.0. (c) Schematic of the graphene-assisted metasurface, consisting of a bottom Ag layer, a middle SiO_2_ spacer, a monolayer graphene and a top nano-block array [209]. (d) The *Q–factor* of the reflection spectra [209]. Figures adopted with permission from Ref. [209]. Copyright 2022 AIP. (e) Schematic of the proposed graphene–Si hybrid system [210]. (f) Evolutions of reflection spectra for the all-dielectric structure under different asymmetric conditions (black solid line) and Fano fitting curves (red dashed line) [210]. Figures adopted with permission from Ref. [210]. Copyright 2023 OSA.

With a continuous graphene monolayer inserting into a dielectric grating and distributed Bragg reflector, as given in Figure 11(a), in 2020 H. Z. Zhong et al. proposed a perfect absorber with high *Q–factor* [208], where the dielectric grating consists of periodic nanowires, and the Bragg reflector composed of five pairs of different dielectric layers. Resulting from the ultralow external leakage loss rate of quasi-BIC resonators and intrinsic absorption loss in the resonant mode, the *Q–factor* of absorption curve reaches more than 1.06 × 10^5^, one or two orders of magnitude larger than that of the existing absorbers. In addition, by introducing a Kerr nonlinear medium, the spectral relative intensity is modulated from 0 to 100 % with an ultra-low pump light of 5 kW·cm^−2^, on the condition that a slight tuning of the refractive index (Δn = 5 × 10^−4^), the absorption contrast ratio is 31 dB. The proposed graphene switcher manifests an obvious spectral wavelength shift of 915 nm/RIU, and the FOM is 50000. Additionally, the graphene layer affects the absorption curve significantly. At low Fermi level, the graphene acts as a dielectric lossy layer. However, on the condition that ℏω≈2μ_c_, the ENZ phenomenon happens, and the graphene layer acts as a lossless material. The proposed graphene tunable absorber is considered as a total reflector thanks to the disappearance of lossy materials. Thus, the amplitude MoD reaches about 100 % if the Fermi level changes in the range of 0.35–0.60 eV. In 2022, Z. Wang et al. proposed a tunable flat band quasi-BIC by inserting a monolayer graphene into nano-brick arrays and SiO_2_ layer [209], which consists of a middle Si layer and two side Ag layers, and the bottom Ag layer acts as a back reflector, as given in Figure 11(c). When the incident wave angle increases from zero to 40°, an ultralow average angular dispersion −0.06 nm/degree is achieved. Since the magnetic polarization keep along the Si block, thus in a wide incident angle range of 0–60°, the proposed graphene-metal-dielectrics-metal structure excites a quasi-BIC resonance, different from the existed structure achieved the quasi-BIC resonance only at a small incident angle. When the incident angle changes in the range of 0–40°, the quasi-BIC resonance maintains a small resonant wavelength offset (<3 nm) and a large absorption amplitude (65 %). The Fermi level affects the *Q–factor* significantly. For the TM mode, as given in Figure 11(d), the *Q–factor* is 52.5 if the Fermi level is zero, and the absorption amplitude is 81 %. As Fermi level increases, the dissipation decreases, and the *Q–factor* reaches to 56.5 at the Fermi level of 0.4 eV. When the Fermi level increases to 0.6 eV, the inter-band transition is blocked, the *Q–factor* of reflection curve reaches 75.9, and the absorption amplitude grows to 95 %. In addition, based on a uniform graphene layer depositing on a periodically arranged amorphous Si cylinder tetramer, W. Xu et al. proposed a dual band tunable absorber, which provides much more choices for the applications of sensors, detectors and absorbers [210], as given in Figure 11(e). By changing the diameters of the diagonal cylinders of the tetramer to introduce asymmetry, dual quasi-BIC resonances are excited, *i.e.* a reflection dip at short wavelength less than 1,000 nm, mode A, and a large reflection peak at long wavelength larger than 1,000 nm, mode B, as given in Figure 11(f). For this graphene supported tetramer structures, the mechanism of mode A results from the magnetic quadrupoles and toroidal dipoles, while for mode B, the EQ and MD moments plays important roles. As the asymmetric parameter increases, the resonant frequencies of mode A and mode B indicate blue shifts and become sharper. The depending of the *Q–factor* for both modes follows an exponential dependence with an exponent of −1.75, *i.e. Q* ∝ *α* ^−1.75^.

**Figure 12:**
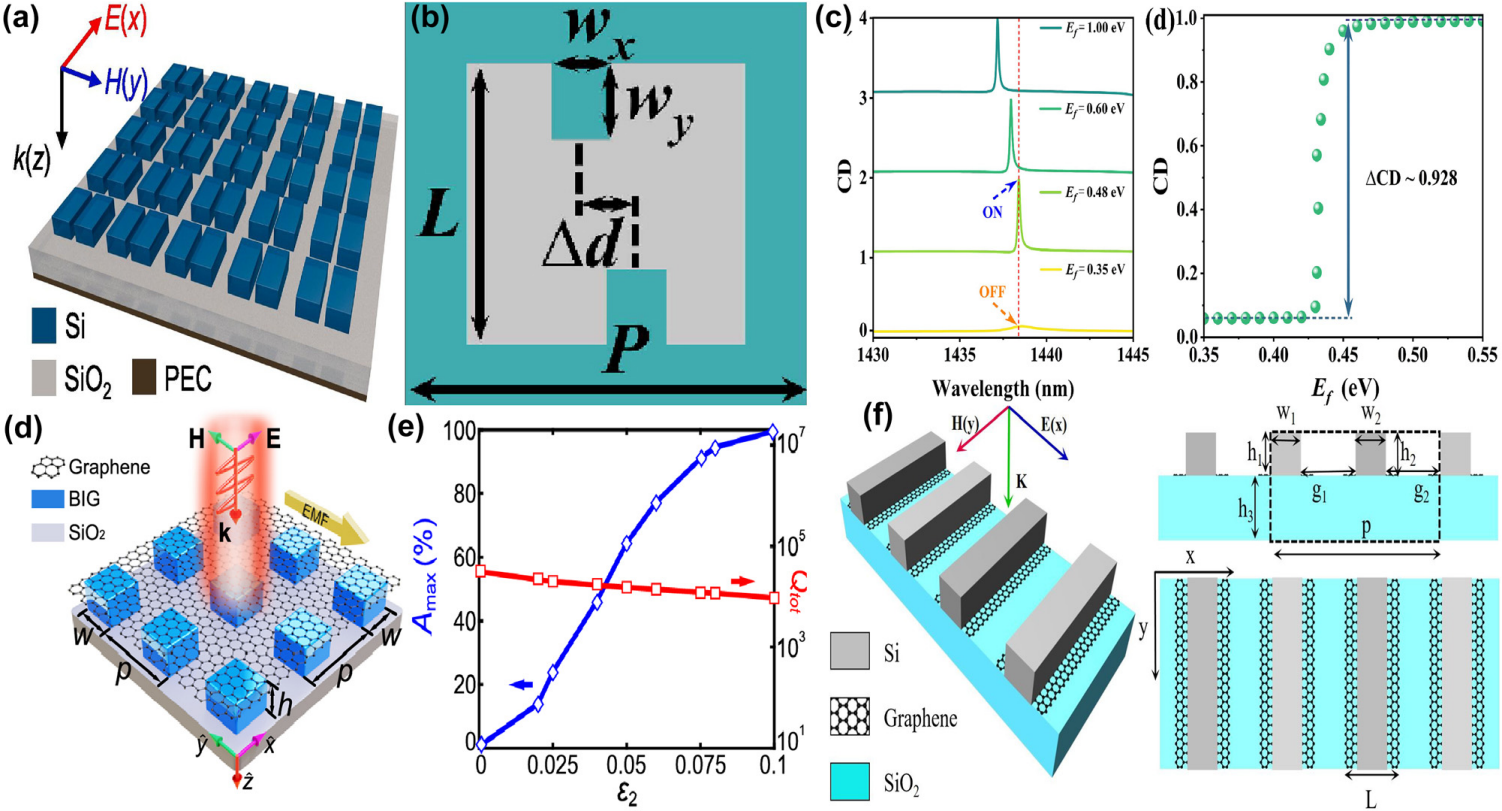
The graphene supported BIC resonance metasurface absorbers with high *Q*-factor. (a) Sketch of the symmetry-protected BIC in a dielectric Si metasurface [211]. Figure adopted with permission from Ref. [211]. Copyright 2024, Elsevier. (b) Schematic diagram of a unit cell of Si metasurface supported chiral BIC, consisting of an array of double-notched cubes deposited on a SiO_2_ substrate [212]. (c) CD spectral lines and peaks at resonance wavelengths of the double-notched cubes structure at different Fermi energies [212]. Figures adopted with permission from Ref. [212] under License CC BY 4.0. (d) The sketch of the graphene supported magneto-optical metasurface with BIG nano-cube array [213]. (e) The maximum value of absorption and *Q–factor* versus the imaginary part of permittivity of BIG nanocube array [213]. Figures adopted with permission from Ref. [213]. Copyright 2023 OSA. (f) Sketch of the proposed graphene-Si grating hybrid structure proposed BIC resonances [214]. Figure adopted with permission from Ref. [214]. Copyright 2024, Elsevier.

By depositing a uniform graphene layer on a periodic narrow-gap Si dimers arrays given in Figure 12(a), X. Y. Zong et al. proposed a tunable near-perfect absorber [211], stemming from the incident wave critical coupling to the mirror-coupled toroidal dipole BIC. By manipulating the distance between Si dimer, the thickness of the spacer layer or geometrical scaling factor, the quasi-BIC can be achieved for the control of the coupling condition. For this graphene-Si dimer hybrid structures, the radiative losses are mainly associated with gap size of dimer and spacer thickness. If the Fermi level is low, *e.g.* 0.1 eV, the graphene manifests a high non-radiative loss, the critical coupling condition is achieved if the gap distance of Si dimer is small. Owing to the large dissipation, the *Q–factor* of absorption curve is relatively lower, with a value of about 350. However, if the Fermi level is 0.5 eV, the graphene permittivity is very small, which corresponds with a very small non-radiative loss. A large gap distance of dimer with a small radiative loss is required to match the critical coupling condition. Thus, the spectral curve of absorption is very sharp with a large *Q–factor* of more than 4800.

As given in Figure 12(b), based on a Si double notched cubes, Z. E. Huang et al. proposed a controllable chiral metasurface with high *Q–factor* [212], which can be efficiently modulated by the extrinsic or intrinsic chiral responses. For the normal incident wave, the intrinsic chiral quasi-BIC is excited by adjusting the structural parameter to break the inverse symmetry, *e.g.* increasing the edge length *g* along the *x* direction. As the value of asymmetric parameter increases from 0 to 70 nm, the CD spectrum evolves from a non-radiative BIC to a radiative chiral quasi-BIC mode with a larger linewidth, and the according *Q–factor* decreases from 6.9 × 10^6^ to 4467. Simultaneously, the value of CD maintains more than 0.96. Additionally, the sharp CD spectra can be tuned efficiently by changing the Fermi level. When the Fermi level at a relatively low value of 0.35 eV, the graphene layer behaves as a lossy dielectric layer and shut-off the CD response. As the Fermi level increases, the contribution of inter-band transition decreases, the dissipation reduces as well, a sharp CD spectrum is observed. Especially on the condition that the *E*_f_ = ℏω/2 is matched, the permittivity of graphene approximates zero with the famous ENZ phenomenon, a large CD value is achieved. As given in Figure 12(c), when the Fermi level changes from 0.35 eV to 0.48 eV, the peak value of CD spectral line is varied from 0.058 to 0.986 with a large MoD about 92.8 %. If the Fermi level increases further, the graphene acts a better plasmonic layer, the peak position of CD spectra manifests an obvious blue shift with a broad spectra curve. Additionally, as given in Figure 12(d), in 2024 by depositing bismuth iron garnet (BIG) nano-cubic arrays on the SiO_2_ substrate, H. S. Zhang et al. at Shenzhen University, China, proposed a graphene supported tunable BIC resonance absorbers with the magneto-optical metasurface in the near-IR spectral region [213]. With breaking the symmetry of the BIG permittivity tensors, the stable quasi-BIC resonant mode is excited, the inherent transverse ED resonance serves as the background mode with high reflection to reducing the radiation dissipation. Furthermore, by introducing the graphene layer as a dissipation source, the absorption reaches more than 99.6 % at the wavelength of 1512.3 nm, surpassing the 50 % absorption limit, and the *Q–factor* is more than 9440, which can be found in Figure 12(e). Additionally, as given in Figure 12(f), by inserting graphene stripes between the Si grating and SiO_2_ substrate, Z. M. Liu *et al. *proposed a dynamical high *Q–factor* quasi-BIC resonance [194]. The results manifests that on the condition that the asymmetric parameter (*α* = (*g*_1_−*g*_2_)/(*g*_1_+*g*_2_)) = 0.025), the *Q–factor* exceeds 39900, and the ultra-sensitive sensors can be obtained with a FOM larger than 19000, Additionally, as Fermi level increases, the transmission dip become stronger and sharper. If the asymmetric parameter is defined as *η*=(*E*_f1_−*E*_f2_)/(*E*_f1_+*E*_f2_), at the resonant frequency of 329.2 THz, the *Q–factor* of reflection peak is about 32000 if the value of η is 0.1.

**Figure 13:**
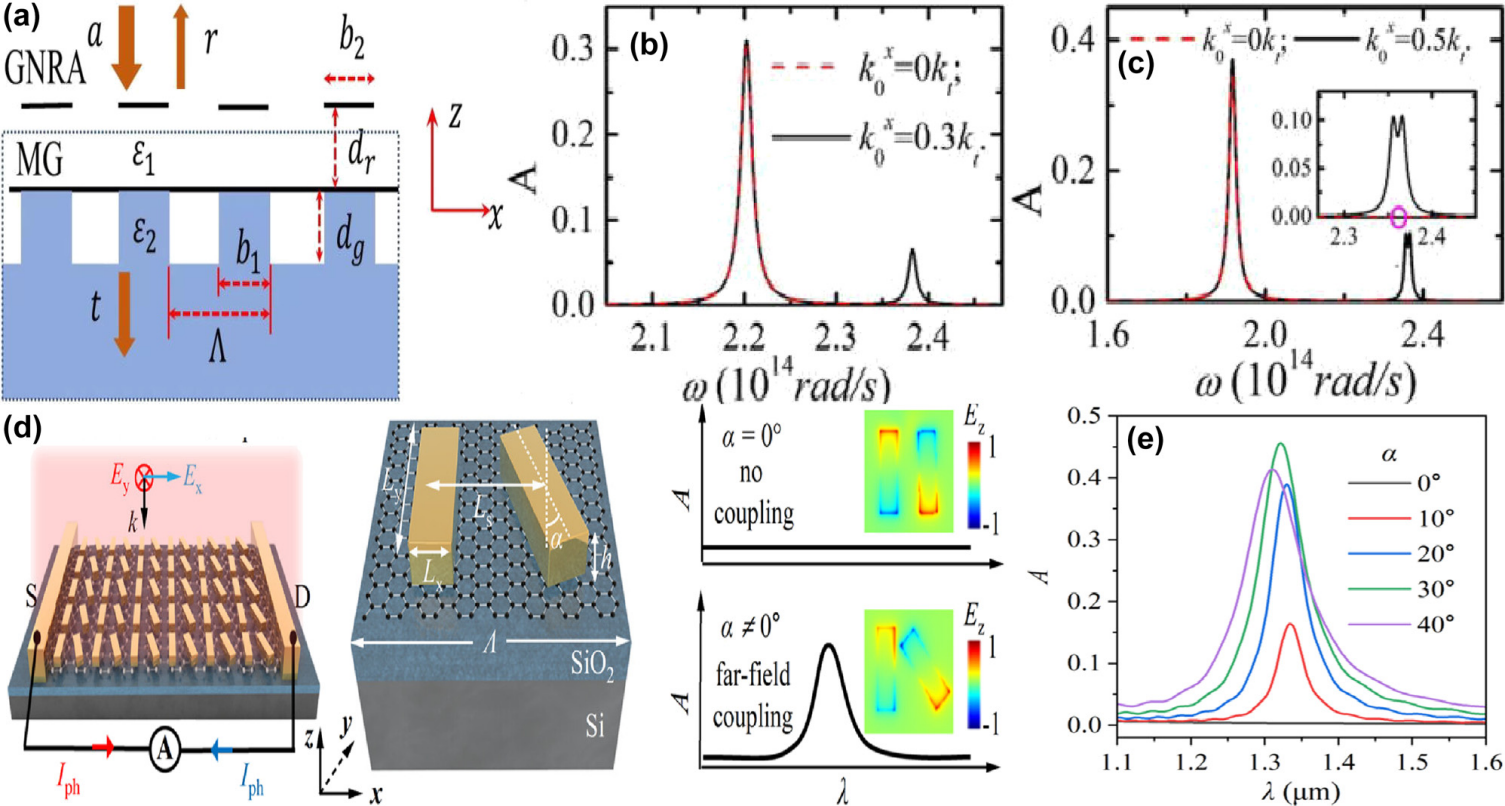
The graphene supported tuanble eBIC metasurface absorbers and photodetector with stripe resonators. (a) The sketch of graphene-dielectric gratings hybrid nanostructure comprising a graphene nanoribbon array, a monolayer graphene, and a binary grating. Figures adopted with permission from Ref. [215]. Copyright 2025 AIP. (b) The absorption spectra of the proposed graphene hybrid waveguide grating for the oblique (black line) and vertical (red line) incident directions. Figures adopted with permission from Ref. [215]. Copyright 2025 AIP. (c) The merging (red circle) of the F–W BIC and S–P BIC resonances. b_2_ = 40 nm, d_r_ = 17.15 nm, and graphene Fermi level was 0.255 eV [215]. Figures adopted with permission from Ref. [215]. Copyright 2025 AIP. (d) Schematic of the plasmonic quasi-BIC metasurface, consisting of asymmetric metallic nanorod pairs on a single-layer graphene, and the absorption curves, electric fields for the symmetric and asymmetric nanorod pairs [216]. Figures adopted with permission from Ref. [216]. Copyright 2025 American Chemical Society. (e) The absorption spectra for the rotated Au nanorod pairs when the rotation angles increased from 0° to 40° [216]. Figures adopted with permission from Ref. [216]. Copyright 2025 American Chemical Society.

As given in Figure 13(a), by depositing a monolayer graphene on binary dielectric gratings, C. M. Huang et al. proposed a tunable flatband F–W BIC resonance [215]. Under oblique incidence, a high lossy leaky mode and sharp S–P BIC mode are observed, which can be found in Figure 13(b). With a transverse magnetic (TM) polarized plane wave impinging on the graphene nanoribbon arrays, a resonant dipolar mode is excited, and interacts strongly with the leaky mode of the dielectric gratings, resulting into the quasi-FW BIC mode as well. Since the proposed quasi-FW BIC and the S–P BIC modes can be independently controlled, thus they can be merged into a robust and high *Q*-factor resonance, less susceptible to the imperfection of device structure. As given in Figure 13(c), a splitting in the absorption curve occurred, arising from the interaction between the imperfectly asymmetric left and right propagating plasmonic waves on the monolayer graphene. Furthermore, this merged BIC resonance can also be adjusted continently via graphene Fermi level in the range of 0.15–0.25 eV or the distance of graphene layer between the dielectric gratings. In 2025, by integrating a periodic array of gold nanorod pair with a uniform graphene layer deposited on a SiO_2_/Si substrate, as shown in Figure 13(d), K. Wu et al. demonstrated a spectral selective and polarization sensitive bipolar photodetectors with a plasmonic quasi-BIC resonance facilitated bulk photo-voltaic effect. By rotating the right nanorod with an angle of **α**, a quasi-BIC resonance is excited. As given in Figure 13(e), when the value of **α** increases to 30°, the absorption peak increases with a maximum value of 0.45, and then decreases with the rotation angle. Due to the strong electric field distribution near the Au nanorod-graphene interface, a strong local Seebeck coefficient gradient in graphene is induced and drives the directional movement of hot electrons to generate a photocurrent, which results into a directional flow of hot carriers and a zero-bias bipolar photocurrent dependent on the polarization of the incident light. Furthermore, the proposed graphene-Au hybrid structure photodetector demonstrates polarization-dependent bipolar photocurrents with spectral selectivity, when the polarization angle varies from 0 to 360°, the photocurrent changed between positive and negative values with a negative polarization ratio of −1. This work gives a good paradigm for the development of miniaturized on-chip devices [216].

#### 2.3.2 Graphene split ring supported Near-IR BIC metamaterials

**Figure 14:**
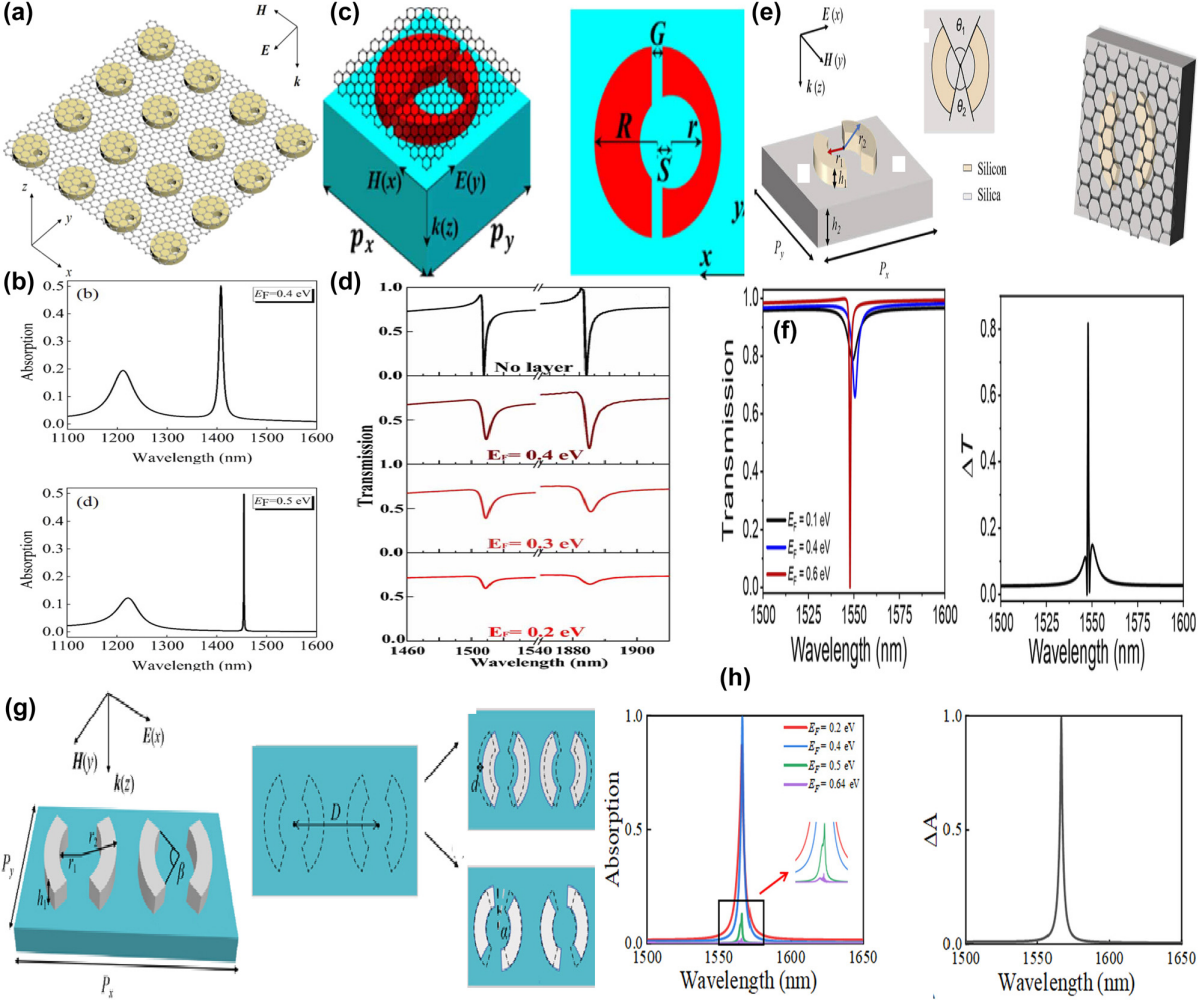
The uniform graphene supported tunable BIC resonance with split ring resonators in the near-IR spectral region. (a) Schematic of the proposed critical coupling system composed of graphene on the silicon nanodisks MMs [120]. (b)The narrowband absorption spectra at critical coupling for monolayer graphene with different Fermi levels of E_F_ = 0.4 and 0.5 eV [120]. Figures adopted with permission from Ref. [120] under License CC BY 4.0. (c) 3D schematic diagram for the proposed hybrid graphene-dielectric metasurface [217]. (d) Transmission spectra of the metasurface without and with the graphene over layer at different Fermi levels [217]. Figures adopted with permission from Ref. [217]. Copyright 2020, IOP. (e) Schematics of the dielectric and the graphene-dielectric metasurface [218]. (f) Transmission spectra of the graphene-dielectric metasurface (*α* = 4°), and the transmission difference between graphene with E_F_ = 0.1 eV and E_F_ = 0.6 eV (α = 4°) [218]. Figures adopted with permission from Ref. [218] under License CC BY 4.0. (g) The sketch of the proposed Si split rings for the double degenerated quasi-BIC resonance [219]. Figures adopted with permission from Ref. [219] under License CC BY 4.0. (h) The absorption curves of the graphene-Si split ring metasurface at different Fermi levels and the absolute absorption difference at graphene Fermi levels of 0.4 eV and 0.64 eV, the value of shift distance of left split ring and rotation angle of the right split ring are 48 nm and 19°, respectively [219]. Figures adopted with permission from Ref. [219] under License CC BY 4.0.

Based on a hybrid structure of graphene with Si split ring nanodisks metasurface, in 2020, S. Y. Xiao’s group at Nanchang University, China, investigated the tunable absorption phenomenon [120], as given in Figure 14(a). By drilling a hole in the Si nanodisks at different locations along the *x* direction, the quasi-BIC resonance is excited owing to the magnetic dipole moment. In the near-IR spectral region, when the Fermi level is low, the graphene membrane can be regarded as a lossy dielectric layer and works as the source of dissipative loss *δ*. On the condition that the radiation rate of quasi-BIC resonance *γ* roughly equals the dissipative loss, the critical coupling condition is satisfied, and the maximum absorption 0.5 can be achieved. As Fermi level increases, the imaginary part of graphene layer permittivity becomes smaller, *i.e.* the dissipative loss reduces, which results into a sharper absorption curve with smaller radiative loss. For example, when the Fermi levels are 0.4 eV and 0.5 eV, the dissipative losses are *δ* = *γ* = 2.254 THz and *δ* = *γ* = 0.201 THz, as given in Figure 14(b), and the according absorption bandwidths are 9.02 nm and 0.9 nm, respectively. Broad absorption curves are also obtained with the graphene multilayers to enhance the dissipative losses. For instance, when the radii of Si nanodisks are 90 nm, 110 nm, 120 nm, 145 nm, and the layer number of graphene are 1, 2, 3, and 7, the maximum absorption resonant wavelengths are 1,400.54 nm, 1,365.73 nm, 1,344.84 nm, and 1,285.83 nm, the according radiation rates and dissipative losses are 2.712, 7.44, 9.078, and 21.375 THz, respectively. Correspondingly, the absorption bandwidth increases from 0.9 nm to 94.53 nm. By depositing an array of Si split rings on a silica substrate, M. Wang et al. proposed a tunable symmetry-protected dual quasi-BICs resonant structure [217], as given in Figure 14(c), a hole is drilled in the Si ring and shifted by a distance along the *x* direction to excite the toroidal dipole and magnetic dipole BIC resonances. When the shift distance is 5 nm, the TD-BIC and MD-BIC resonances are both excited at low and high frequencies, respectively. As the shift distance increases, the TD-QBIC (MD-QBIC) indicates a red-shift (blue shift), the resonant strength becomes stronger, while the spectral curve becomes wider with a low *Q–factor*. For the hybrid structures of a graphene-asymmetric Si split ring array, when the Fermi level is low, *e.g.* 0.2 eV, inter-band transition plays an important role, and the graphene membrane acts as a lossy dielectric material, the TD-QBIC and MD-BIC resonant strength become very weak and almost disappear. As given in Figure 14(d), in the near-IR spectral region as Fermi level increases and the graphene layer permittivity decreases, the contribution of inter-band transition reduces, and the resonant strength becomes stronger. When the Fermi level is about 0.4 eV, the inter-band transition is almost blocked, and the graphene permittivity approximates zero. Thus, the resonant strength of double quasi-BIC resonances become stronger. Considering the critical transition frequency of ℏω≈2μ_c_, *e.g.* 0.4 eV for this structure, the transition frequency is relatively lower, and the MD-QBIC resonance is more sensitive to the variance of graphene Fermi level, which indicates better tunable properties. Additionally, when the polarization changes in the range of 0–90°, the amplitude MD reaches about 100 %, and resonant wavelength almost remains unchanged.

In 2021, by utilizing the hybrid structures of graphene-Si split ring arrays given in Figure 14(e), G. D. Liu et al. proposed a tunable quasi-BIC resonance [218], *θ*_1_ and *θ*_2_ are the angles of the upper and bottom sections of the split rings. The asymmetric parameter *α* is defined by *α* = sin(*θ*_1_-*θ*_2_)/2.0. When the asymmetric parameter is 3°, an obvious quasi-BIC resonance is observed, and the *Q–factor* reaches more than 10^4^. Due to the strong interaction, the resonant curves are closely associated with the gap distance between the graphene with Si split ring. If the gap distance is not very large, *e.g.* less than 200 nm, its effects can be neglected, the absorption is about 50 %. The transmission curve can also be modulated conveniently with graphene Fermi level. If the Fermi level is 0.1 eV, the loss is very large, and the transmission quasi-BIC indicates a broad and shallow dip with a very low *Q–factor*. As Fermi level increases, the graphene permittivity decreases, the transmission dip becomes sharper and deeper. Especially, if the Fermi level is 0.6 eV, in the ENZ region the transmission curves shows a very deep and sharp dip with a large *Q–factor*. When the Fermi level changes in the range of 0.1–0.6 eV, the amplitude MoD is about 81 %, which can be found in Figure 14(f). Recently, as given in Figure 14(h), they also proposed a tunable high *Q*-factor absorber with double Si split rings deposited on a SiO_2_ substrate [219], which achieves a perfect absorption by the simultaneous excitation of orthogonal MD modes via double degenerated quasi-BIC resonances, eliminating the requirement of asymmetric illumination or reflective backing. By moving the centers of the two split-ring in the left toward the center *y* direction d or rotating the center axis of split-ring along the *z*-direction with a rotation angle *α*, double quasi-BIC resonances are excited at the wavelengths of 1,554.4 nm and 1,558.6 nm, respectively. With the optimized structural parameters (*α* = 19°, *d* = 40 nm), the double quasi-BIC modes gradually approach and eventually coincide at the wavelength 1,562 nm. Thus, a near-unity absorption is demonstrated and can be modulated effectively with the help of a uniform graphene layer. Due to the significant variance of graphene permittivity at different Fermi level, a mode splitting of absorption spectra is observed. As the Fermi level increases from 0.2 to 0.4 eV, the absorption increases to near unity when the graphene membrane acts as an ENZ layer, while if the Fermi increases further to 0.64 eV, the graphene layer manifests a high dissipation plasmonic layer, the absorption reduces to near zero, which can be found in Figure 14(h).

**Figure 15:**
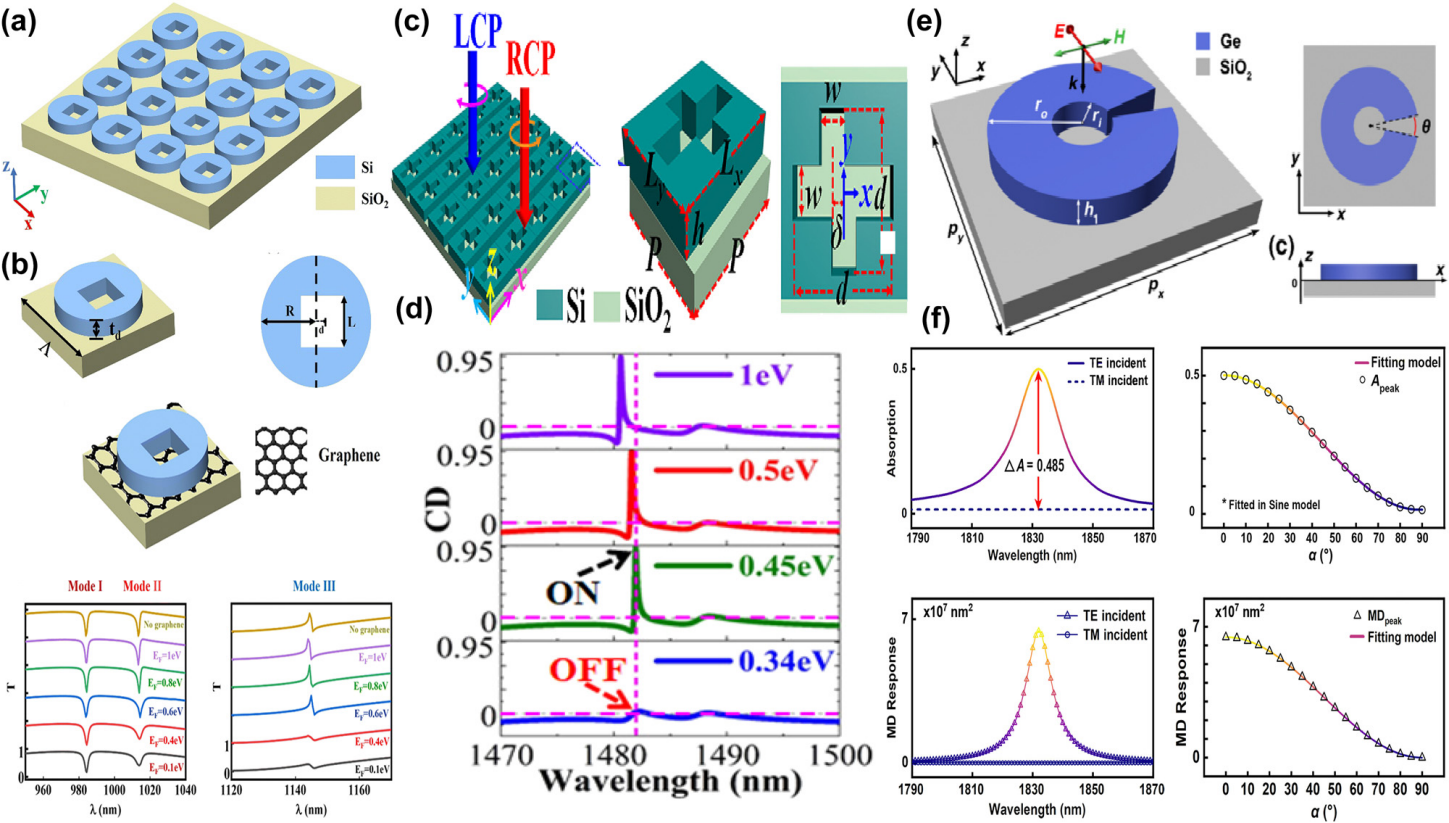
The uniform graphene supported tunable near-IR BIC resonance with Si split ring resonators. (a) Design concept of the proposed asymmetric Si metasurface with rectangular hole array [220]. (b) The optical modulation of the Si–graphene composite metasurface, the displacement *d* = 8 nm, *R* = 250 nm, *L* = 170 nm, and t_d_ = 230 nm [220]. Figures adopted with permission from Ref. [220]. Copyright 2024, Royal Society of Chemistry. (c) Schematics of the Si hollow asymmetric cross hole supported quasi-BIC metasurface [221]. (d) The effects of graphene Fermi levels on the circular dichroism [221]. Figures adopted with permission from Ref. [221]. Copyright 2024, OSA. (e) The schematics of the proposed graphene-Ge metasurface [222]. (f) The absorption spectra of the critical coupling system under *α* = 0° and 90°, the declined curve of the peak absorbing intensity, and the modulation depth of the metasurface when *θ* = 7.1° [222]. Figures adopted with permission from Ref. [222]. Copyright 2024, APS.

With a hollow Si nano-disk array, as given in Figure 15(a), J. K. Liu et al. proposed a tunable quasi-BIC resonance [220]. By moving the rectangular hole along the *x* direction to introduce the asymmetric parameter, triple Fano resonances are achieved. As the displacement increases, the resonant peak wavelength for mode I and mode III increases, the spectral width increases with low *Q–factor*, which is reduced from 2700 to 130, and 4160 to 320, respectively. The analysis of the multipolar decomposition shows that mode I results from the TD mode, while mode III results from the MQ and EQ moments. For TD resonant mode I, the influence of graphene layer is not significant. For the ED mode II, if the Fermi level is low, it acts as a lossy dielectric layer.

As Fermi level increases, the graphene membrane loss reduces, and the resonant dip becomes sharper and stronger. When the Fermi level is larger than 0.5 eV, the permittivity of graphene approximates zero, *i.e.* entering into the ENZ region, thus the influences of graphene layer can be neglected. As for the case of mode III, if Fermi level is larger than 0.5 eV, the dissipation of graphene layer decreases slightly, and its effect is not significant. However, if the graphene Fermi level is smaller than 0.5 eV, the loss increases obviously as the Fermi level decreases, as given in Figure 15(b). When the Fermi level is 0.1 eV, the transmission peak of mode III almost disappears owing to the large dissipation of graphene membrane. The proposed graphene–Si hybrid structure also indicates good sensitivity, for mode I, II, and III, the sensitivities are 136 nm/RIU, 162 nm/RIU, and 109 nm/RIU, and the according FOM are 144.68, 170.523, and 155.71/RIU, respectively. Based on the Si nano-cubes etched with asymmetric cross-shaped air pillars on the SiO_2_ substrate, Y. Cheng et al. proposed a tunable approach to achieve near-unity CD [221], as given in Figure 15(c). Using ultra-narrow Au peak or deep valley (*δ* = 25 nm or −25 nm, *δ* is the offset between the centers of the translated part and cross-shaped air pillars), giant CD chirality is demonstrated. Due to the excitation of low loss toroidal resonance, the *Q–factor* reaches more than 20000, and the maximum CD is 0.94. If the Fermi level is low, *e.g.* smaller than 0.34 eV, the graphene layer manifests a lossy dielectric layer, and reduces the value of CD significantly down to 0.03. When the Fermi level is 0.45 eV, graphene layer enters the ENZ region, its influences can be neglected. As given in Figure 15(d), the value of CD is as large as 0.93. By changing the bias voltage, the CD intensity can be converted from 0.03 (“OFF”) to 0.93 (“ON”). The analysis of electric field distribution also indicates that the localized field enhancement reaches 12 times and 7 times for the RCP and LCP incident waves when the Fermi level varies in the range of 0.34–1.0 eV. In 2024, by integrating a uniform graphene layer with a C-shaped Ge split ring depositing on a quartz substrate, as given in Figure 15(e), Z. L. Li et al. demonstrated polarization-modulating switchable absorber [222], the split angle θ is introduced to excite a S–P BIC resonance, the asymmetry parameter is defined as s = sin(*θ*/2.0). The excited quasi-BIC resonance significantly boosts the incident wave-graphene interaction and enhances the absorption. The critical-coupling condition is achieved by optimizing the Fermi level of graphene and the asymmetry parameter of the metasurface. As the Fermi level increases, the dissipation of graphene membrane reduces, the split angle to match the critical condition decreases, and the absorption peak indicates a red-shift with a smaller FWHM. Since the quasi-BIC resonance mainly stemmed from the domination of low dissipation of MD response, the *Q–factor* of absorption peak reaches more than 200. Additionally, the intensity of those critical-coupling absorption is efficiently modulated by the polarization of the incident waves with a high value of 97.1 %, the maximal difference of the absorbing intensity is about 0.485 for the TE and TM modes, which can be found in Figure 15(f).

**Figure 16:**
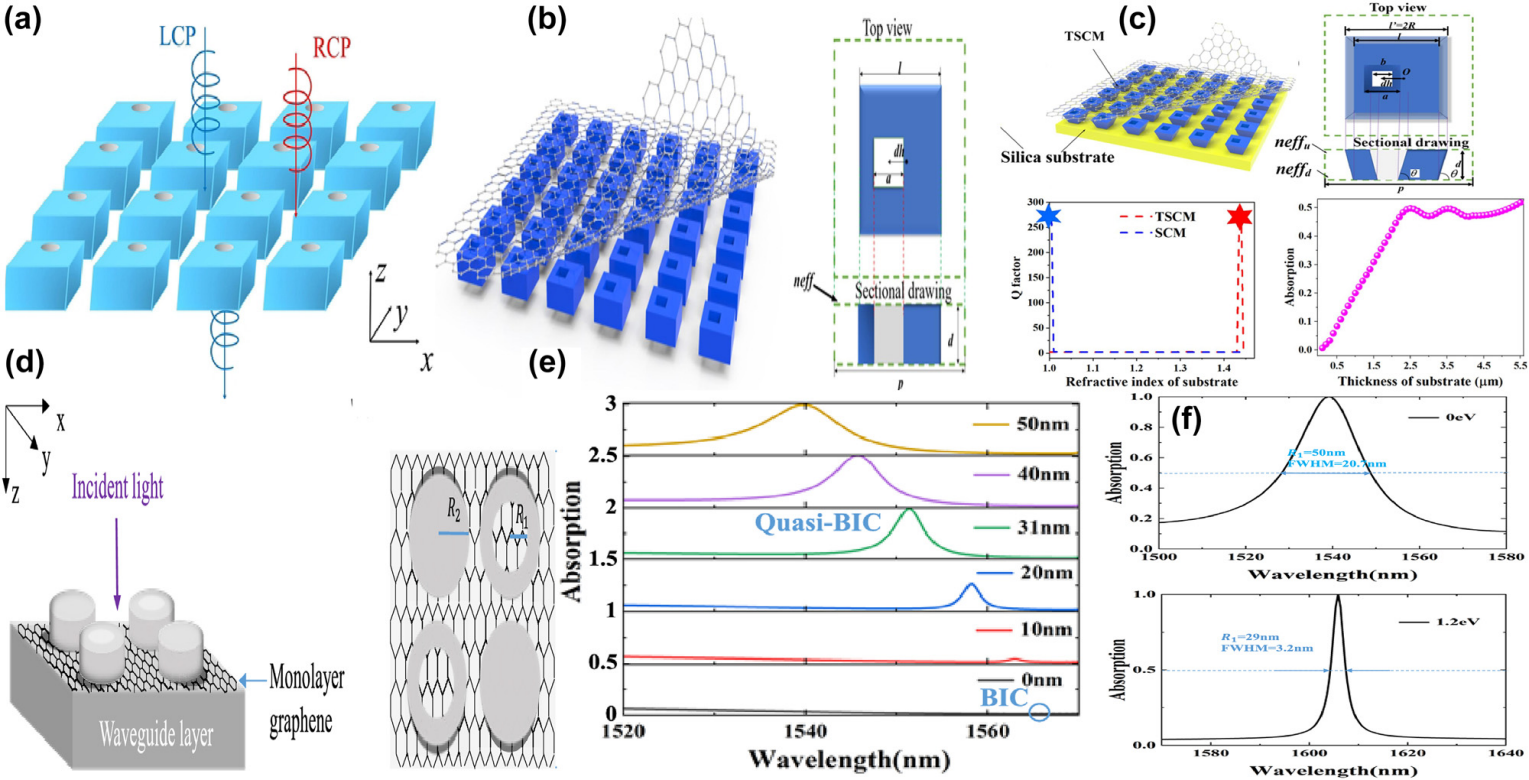
The uniform graphene supported tuanble near-IR BIC absorbers with asymmetric split ring resonators. (a) Schematic of the supported chiral quasi-BIC structure of the silicon metasurface, which consists of a tilted cuboid structure with holes, and the incident circularly polarized light was injected perpendicularly along the negative *z* direction [223]. Figures adopted with permission from Ref. [223] under License CC BY 4.0. (b) The square column is attached with graphene and top view and profile of the structure [224]. (c) Tapered square column metasurfaces were attached with graphene and silica substrate, its *Q–factor* varying with the refractive index of the substrate (the blue line was the square column metasurfaces structure, the red line is the tapered structure), and the absorption rate of silica substrate thickness scanning [224]. Figures adopted with permission from Ref. [224]. Copyright 2024, Elsevier. (d) The schematic illustration of the composite Ge metasurface structure, with *R*_1_ denoting the inner radius of the annulus, *R*_2_ indicating the outer radius of the annulus [225]. (e) Absorption spectra versus wavelength at different inner radii, the Fermi level of graphene was 0.6 eV [225]. (f) Absorption spectra meeting the critical coupling conditions for E_F_ = 0 eV and E_F_ = 1.2 eV. Figures adopted with permission from Ref. [225] under License CC BY 4.0.

By integrating a uniform graphene layer with a tilted cuboid hole onto Si layer, as given in Figure 16(a), S. Y. Lv* et al.* proposed a tunable selective MMs absorber in the near-IR spectral region driven by the intrinsically chiral quasi-BIC resonance [223], which can be achieved and precisely controlled by breaking the in-plane and out-of-plane symmetry of the structure. The extrinsic chirality by the obliquely incident circularly polarized waves is demonstrated without introducing the out-of-plane asymmetry of the structure. The circular dichrosim is nearly unit and the ultra-high *Q–factor* with the value of 3722 is achieved. With a graphene-Si hybrid metasurface, selective absorption of intensity-controlled right/left-handed circularly polarized light (RCP/LCP) is achieved by modulating the Fermi level and out-of-plane tilted angle. For the practical quasi-BIC resonance, if the substrate is added, the out-plane symmetry is destroyed and the original high absorption from graphene layer reduces significantly. To solve this problem, as given in Figure 16(b), in 2024 H. Xu* et al.* proposed the tapered resonator structure to avoid the mode leakage into the substrate and maintained the high absorption rate of graphene metasurface [224]. For instance, for the silicon-based periodic square column metasurfaces embedded with a monolayer graphene, the absorption curve shows a peak near 1.5 μm and the MD moment dominates. However, when a silica substrate is taken into accounted, the absorption peak disappears and the TD moment plays an important role. With the taped resonators, the absorption peak is observed and the MD moment dominates again, and the *Q–factor* reaches about more than 250, which can be found in Figure 16(c). This proposed means is universal for many different kinds of resonators. However, it should be noted that for the tapered SRR, due to the breaking of resonator, the MD moment can’t be excited, while and EQ moment itself don’t radiate along the *z* direction and satisfies the S–P BIC resonance. By inserting a uniform graphene between periodic asymmetric Ge metasurface and SiO_2_ waveguide-metal layers, as given in Figure 16(d), Y. Liu et al. proposed a bandwidth-tunable perfect absorber by introducing a quasi-BIC resonance [225]. Consisting of two discs and annuli with the same outer radius *R*_2_, the proposed periodic resonators are arranged in a square lattice, the inner radius *R*_1_ along the diagonal line is introduced as the asymmetric parameter. When the radius of the inner radius increases from 10 nm to 50 nm, an obvious absorption peak is observed and become broader with smaller *Q–factor*, as given in Figure 16(e), the absorption peak indicates a blue shift, which means that the radiative loss rate at the quasi-BIC resonance can be modified by altering the structural parameters. At large inner radius, *e.g.* 50 nm, the radiation loss is relatively higher, the lower Fermi level of graphene layer, is required to match the critical coupling condition, the FWHM of the absorption peak is about 20.7 nm. As the inner radius decreases, the radiation loss reduces, graphene layer with larger Fermi level should be utilized to match the critical condition. For instance, if the radius is 29 nm, the graphene Fermi level is about 1.2 eV to match the critical condition, and the FWHM of absorption peak is only about 3.2 nm, which can be found in Figure 16(f).

### 2.4 The nonlinear harmonic effects of graphene BIC metamaterials

Compared to the plasmonic counterparts, high-index dielectric nanostructures have low material losses and provide both electric and magnetic field enhancement in bulk volumes, enhancing the highly efficient nonlinear frequency conversion. BIC resonance improves the nonlinear effects obviously and provides an excellent platform for exploring frequency conversion, high-harmonic generation and the manipulation of quantum states. Besides using nanoparticles, the nonlinear optical processes in resonant metasurfaces can also be substantially boosted by manipulating the linear optical response in the quasi-BIC region through structure optimization. The field enhancement from quasi-BIC resonance is utilized to achieve high-efficiency harmonic generation in dielectric metasurfaces, including high-harmonic generation with non-perturbative features such as saturation in power scaling.

The nonlinear optical responses of graphene layer are very strong, but due to the thin thickness nature of 2D materials, the according optical responses are seriously limited by the short interaction length with incident waves. Based on the Mie-resonances, all-dielectric Si metasurface displays field enhancement over a large area and improves the large efficient nonlinear processes over a large area. By introducing the BIC resonance into the dielectric metasurface system, the strong optical nonlinear resonance and the conversion efficiency of nonlinear responses can be improved, such as second-harmonic, third-harmonic, and four wave mixing generation efficiency. In the following part, we will give a description in these aspects.

**Figure 17:**
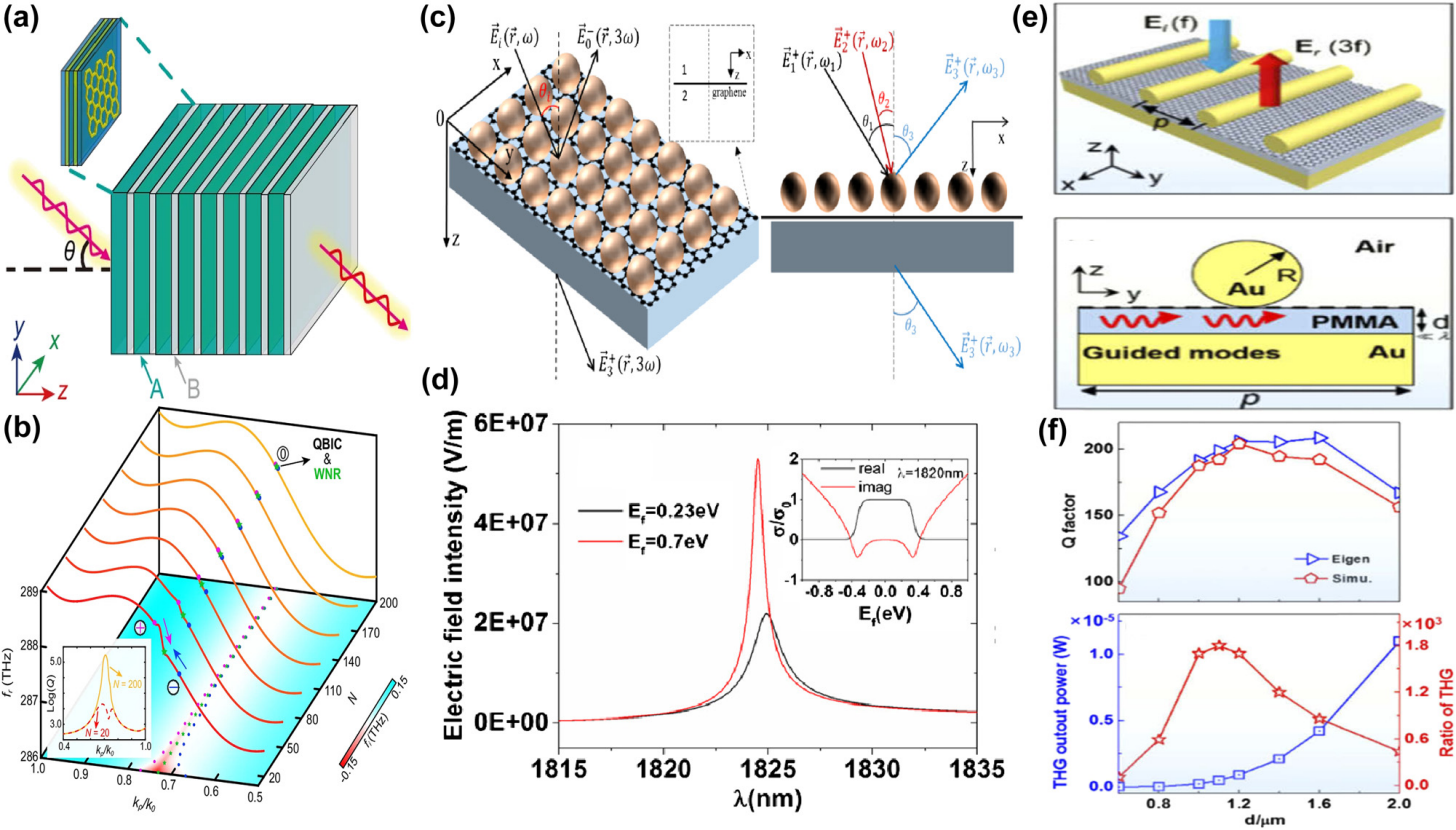
The enhancement of nonlinear harmonic effects with graphene BIC resonance metamaterials. (a) The scheme of a 1D hyper-crystal: (AB)N, where the electric HMM layer A is mimicked by subwavelength dielectric/graphene/metal stacks [226]. (b) Reflection-zero dispersion for bilateral drumhead surface state of the photonic crystals in the complex frequency space for the TM wave [226]. Figures adopted with permission from Ref. [226] under License CC BY 4.0. (c) Diagram of the sphere-graphene-slab structure and the TH generation process [227]. (d) The absorption as a function of the wavelength when the Fermi energy is taken as *E*_f_ = 0.23 eV (black line) and *E*_f_ = 0.70 eV (red line), inset shows the conductivity of the graphene [227]. Figures adopted with permission from Ref. [227] under License CC BY 4.0. (e) Schematic diagrams of the designed metasurface was a periodic array of gold cylinder, laid on a graphene/dielectric/metal substrate along the *y* direction [228]. (f) Influence of dielectric layer thickness on the *Q–factor*s and the THG output power. Adopted with permission from Ref. [228]. Figures adopted with permission from Ref. [228] under License CC BY 4.0.

As given in Figure 17(a), S. Y. Hu et al. proposed a 1D photonic crystals structure to excite BIC resonance by utilizing hyperbolic MMs controllable photonic Weyl nodal line semimetals in the near-IR spectral region both dynamically and topologically [226], in which A is the hyperbolic MMs with substructure dielectric/graphene/metal stacks (CGD), B and C are BeO (ε_BeO_ = 1.708) and GaAs (ε_GaAs_ = 3.48) with slight dispersion near the working frequency, and the indium tin oxide (ITO) is adopted as the metal layer. Composed of two uncoupled Weyl nodal rings with different polarizations, the suggested 1D hyper-crystals with four degrees of freedom supported the phase transitions of translation and rotation were realized by flexibly modulating the Fermi level of graphene and the thicknesses of component layers, respectively. Due to the resonators without inversion symmetry, a pair of reflection-phase singularities carrying opposite topological charges emerge near each nodal line. With reducing radiation leakages and absorption losses, these singularities gather together and form a scattering-matrix singularity, *i.e.* quasi-BIC ring at the nodal line ultimately. The peaks of the *Q–factor* appear near the position of singularities, as shown in Figure 17(b), implying that most energy is localized in the composite structure. This work reports the first realization of controllable photonics Weyl nodal line semimetals and establishes a bridge between BICs and WSM via hyper-crystal. Based on a three-layered structure, *i.e.* a dielectric thallium iodide spheres arranged in a square lattice, a uniform graphene layer and a dielectric slab, in 2017 Wang and Zhang investigated the third-order nonlinear effects based on the BIC resonance [227], as given in Figure 17(c). When the near-IR spectral wave is incident on the graphene-particle arrays structure with an oblique angle of 5°, the BIC resonance is excited and enhances the nonlinear effects significantly owing to the improved field localization. As given in Figure 17(d), at low Fermi level of *E*_f_ = 0.23 eV, the graphene layer manifests a lossy dielectric layer with large dissipation; while for the Fermi level of 0.7 eV, the graphene layer inhibits smaller dissipation, indicating stronger localization of the electric fields. Correspondingly, the FWHM at larger Fermi level is much smaller, *e.g.* the relative line widths are 0.0356 % and 0.0175 % when the Fermi levels are 0.23 eV and 0.70 eV, respectively. The THG efficiency is enhanced by about 5 and 7 orders of magnitude for the Fermi level at 0.23 eV and 0.7 eV. In 2020, by depositing a periodic array of Au cylindrical wire on a graphene/dielectric layer/metal substrate, as given in Figure 17(e), Liu et al. proposed an enhanced third harmonic generation (THG) in the THz region [228], which is attributed to the hybrid guided mode from the interaction of the metal cylinder array with the dielectrics, propagating in the dielectric layer below. The incident THz waves firstly interacts with the nonlinear medium and excites a weak THG, and then it is significantly boosted on the condition that it is coincided with the resonance. As the thickness of the dielectric spacer layer increases, the *Q–factor* shows a peak with a maximum value of about 200, as given in Figure 17(f). The THG power increases with the metal wire radius, and the maximum value is more than 1.0 × 10^−5^, the ratio of THG (the output power to THG with metal cylinder array to that without metal wire array) reaches a maximum of 1800 if the layer thickness is about 1.1 μm.

**Figure 18:**
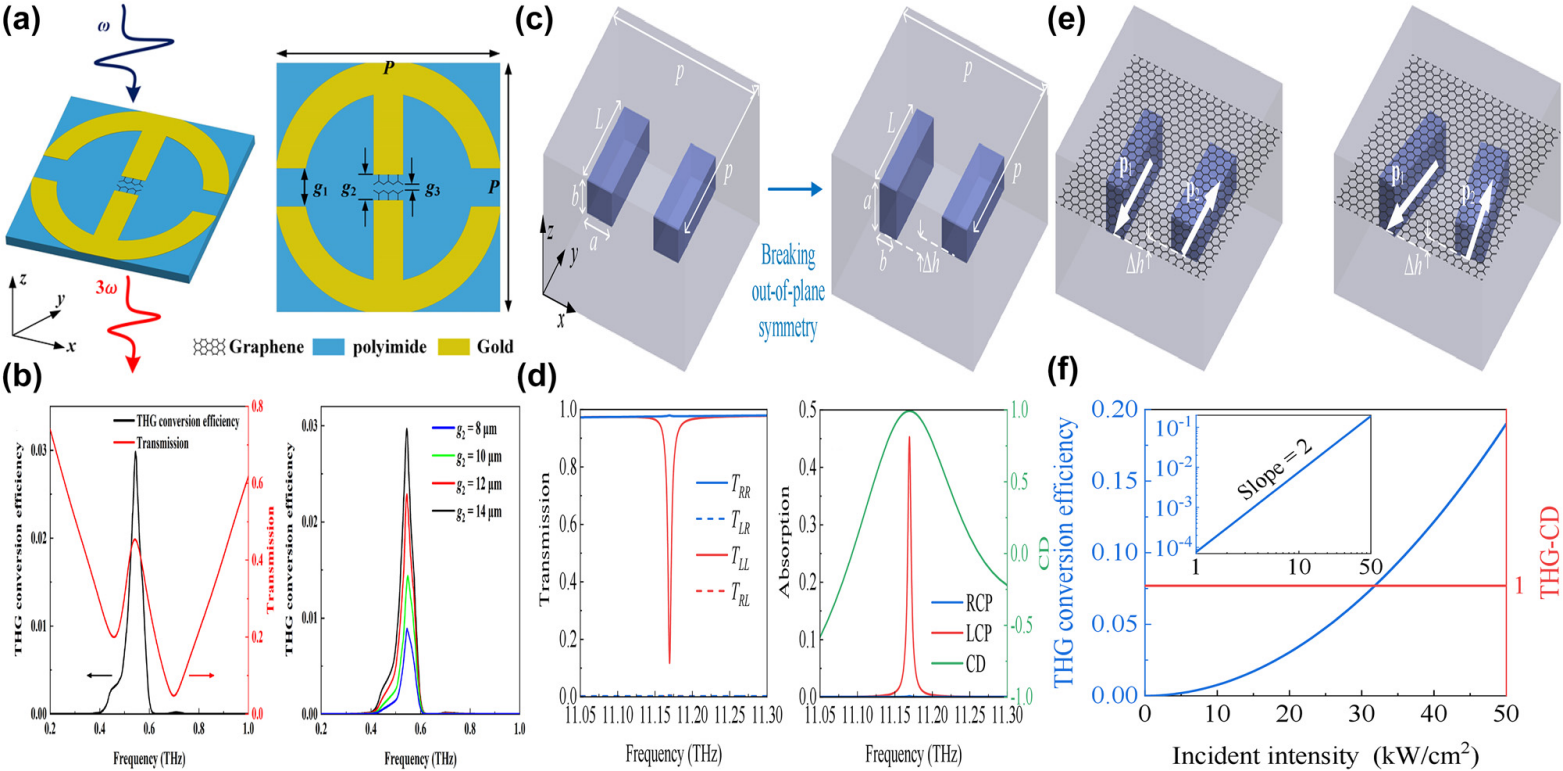
The graphene supported tunable THz nonlinear metasurface with BIC resonance. (a) Schematic of the unit cell of the proposed graphene-metal hybrid THz nonlinear metasurface [229]. (b) THG conversion efficiency and quasi-BIC transmission for the graphene–metal metasurface at Fermi level E_f_ = 0.2 eV and the THG conversion efficiency as a function of fundamental frequency for different gap *g*_2_ [229]. Figures adopted with permission from Ref. [229]. Copyright 2023 AIP. (c) Schematic of the metasurface with symmetry and without symmetry by turning one bar 90° around the *y*-axis [230]. (d) Transmission spectra under RCP and LCP incidences and the according absorption spectra of RCP and LCP and CD spectra [230]. (e) Graphene metasurface with turning one bar out of plane and with turning one bar out of plane and rotating the bars in of plane [230]. (f) LCP THG conversion efficiency and THG-CD dependence of the incident intensity at 11.17 THz. The inset is corresponding log–log plot for LCP THG conversion efficiency [230]. Figures adopted with permission from Ref. [230] under License CC BY 4.0.

As given in Figure 18(a), B. K. Wang *et al*. proposed an enhanced THz THG by utilizing graphene-metal hybrid metasurface with quasi-BIC resonance, which consists of an Au circular split ring, monolayer graphene rectangular stripe with a gap, and polyimide thin substrate [229]. Based on the strong localized effect of the parameter-BIC mode, the conversion efficiency of the nonlinear process can be powerfully enhanced. Particularly, when a graphene rectangular ribbon with high Fermi level of 1.0 eV is inserted into metal stripes, the graphene-metal hybrid metasurface behaves like a split ring with a gap of 2 μm. By utilizing the strong confinement at the quasi-BIC resonant frequency and the large third-order nonlinearity of graphene layer, the THG conversion efficiently is enhanced effectively. The result manifests that the THG conversion efficiency is proportional to the square of the incident intensity, and reaches a storable value about 3 %, which is much larger than that of the existing work, usually less than 1 %, as given in Figure 18(b). As Fermi level increases, the local field enhances, while the nonlinear effects of graphene decrease significantly.

In 2024, they also proposed a strong chirality metasurface based on the quasi-BIC resonance of a hybrid graphene-dielectric Si dimer [230], which can be found in Figure 18(c). By rotating the dimers in plane with a diverging angle *θ* and then moving one bar out of plane, the electric dipoles of the bars are no longer antiparallel. Considering the height difference of those two bars, the hybrid resonator manifests 3D chirality. When the perturbations of the dipole displacement and diverging angle are balanced, the graphene-Si hybrid resonator is efficiently coupled to LCP wave via the quasi-BIC resonance while its response to RCP is very weak, resulting into a strong chirality. Near the quasi-BIC resonant frequency, the Si-graphene hybrid metasurface exhibits an enhancement of nonlinear chirality, strong absorption for the LCP waves and little response to the RCP waves, and the according values are 0.46 and 0.002, thus the value of CD is 0.99, nearly maximum chirality, as given in Figure 18(d). Additionally, due to the robust third-order nonlinear susceptibility of graphene layer, the proposed hybrid dimer manifests strong THG phenomenon, which is found in Figure 18(e). Near the quasi-BIC resonant frequency of 11.17 THz, and the pumping intensity of 50 kW/cm^2^, both LCP and RCP indicate district peaks, while the magnitude of LCP is seven orders of magnitude stronger than that of the RCP. As the incident wave intensity increases, as shown in Figure 18(f), the THG conversion efficiency increases and reaches more than 19 % when the intensity is 50 kW/cm^2^, and the value of THG-CD is unity.

## 3 3D Dirac semimetal and other novel emerge materials supported BIC metamaterials

Similar to graphene layer, 3D DSM layer inhibits strong light confinement, low dissipation, and excellent tunable conductivity. Furthermore, 3D DSM has also several advantages, such as the higher mobility, surmounting the restriction of thickness and an additional structural DoF in the construction of functional devices. 3D DSM provides a good platform to designing novel and flexible functional devices.

Under the framework of Kubo formalism in random phase approximation, the longitudinal complex dynamic conductivity of the 3D DSM can be expressed as [231], [232], [233], [234], [235]:

(3) ReσDSΩ=e2ℏgkF24πΩGΩ/2

(4) ImσDSΩ=e2ℏgkF24π24Ω1+π23TEF2+8Ω∫0εcGε−GΩ/2Ω2−4ε2εdε

in which *G*(*E*) = *n*(−*E*)−*n*(*E*), *n*(*E*) is the Fermi distribution function, *E*_*F*_ indicates the Fermi level, *k*_*F*_ denotes the Fermi wave-vector, **Ω** = ℏ**ω**/*E*_*F*_, *k*_F_ = *E*_*F*_/ℏ*v*_*F*_ represents the Fermi momentum, *v*_*F*_ is Fermi velocity, *E*_*c*_ remarks the cutoff energy beyond which the Dirac spectrum is no longer linear, *g* is the degeneracy factor.

The permittivity of 3D Dirac semimetals can be obtained using the following formula,

(5) $$\varepsilon_{\text{DSM}}=\varepsilon_{\infty }+i\sigma_{\text{DS}}/\omega \varepsilon_{0}$$

where *ε*_b_ is the effective background dielectric (*ε*_*b*_ = 1, *g* = 40, for AlCuFe quasi-crystals), *ε*_0_ is the permittivity of vacuum.

### 3.1 3D Dirac semimetal supported BIC metamaterials

**Figure 19:**
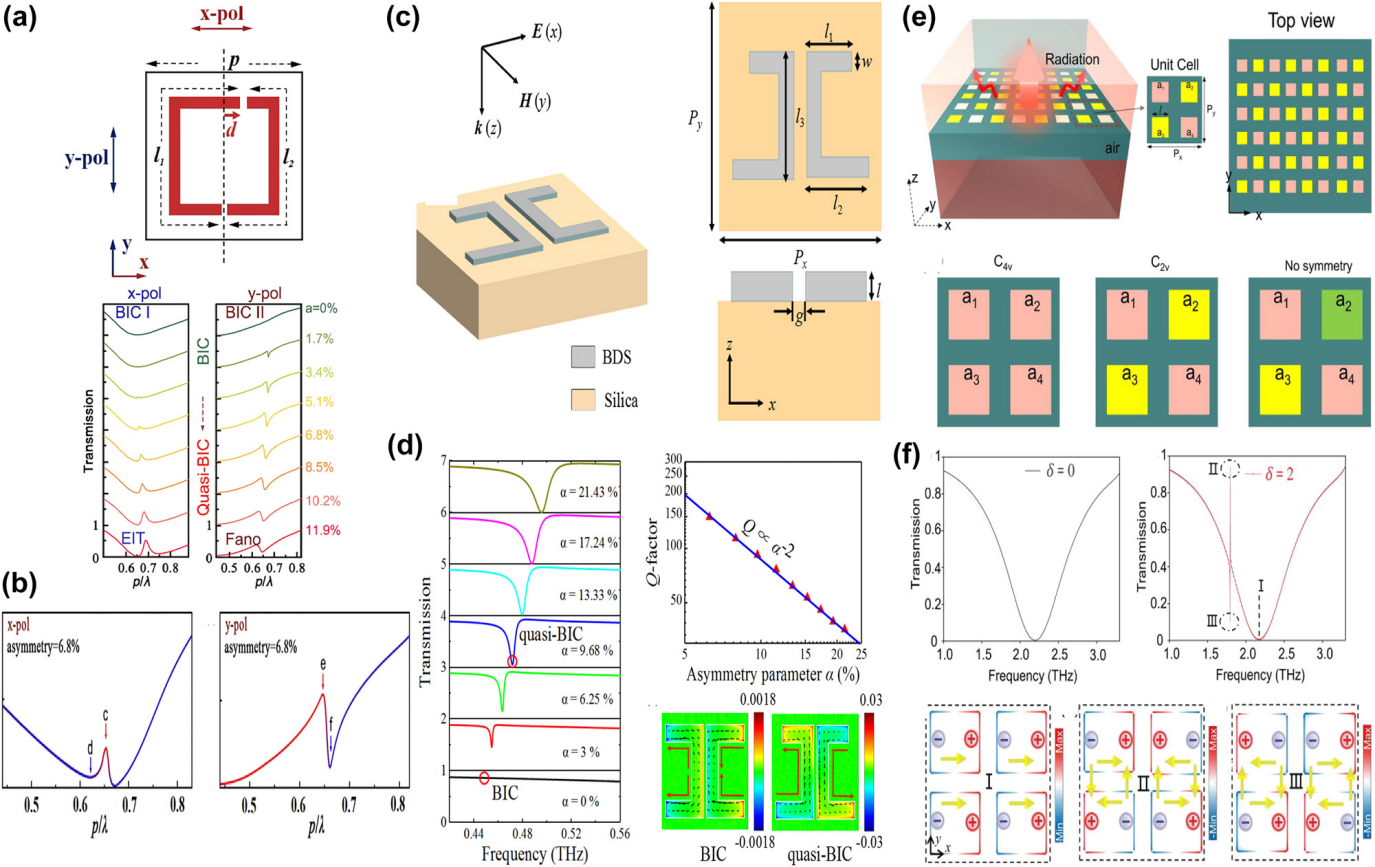
The 3D DSM supported tuanble BIC metasurface in the THz region. (a) The sketch of the proposed 3D DSM split-ring resonators with symmetry breaking, the influences of the asymmetric parameter on the transmission curves for the *x*-polarization and *y*-polarization [236]. (b) The transmission curves and the distribution of current density and the *z*-component of the electric fields for the *x-* and *y*-polarization [236]. Figures adopted with permission from Ref. [236]. Copyright 2022, IOP publishing. (c) The schematic sketch of the proposed 3D DSM MMs structures [237]. (d) The influences of the asymmetric parameter on the transmission curves, *Q–factor*s, and the surface current magnitude and vector distribution [237]. Figures adopted with permission from Ref. [237]. Copyright 2022, IOP publishing. (e) The proposed 3D DSM C_4v_ structural resonators, the Fermi levels for the a_1_, a_2_, a_3_, and a_4_ can be manipulated with bias voltage [238]. (f) The resonant curves of the proposed 3D DSM structure for the isotropic and anisotropic MMs resonators (The Fermi levels of a_1_ and a_4_ are 50 meV, a_2_ and a_3_ are the 48 meV), the electric field distribution for the points I, II, and III [238]. Figures adopted with permission from Ref. [238]. Copyright 2022 AIP.

Recently, 3D DSM metamaterials supported BIC resonance arise researchers’ attention and develop very quickly. For instance, by utilizing 3D DSM asymmetric split ring resonators, Ma et al. suggested a tunable DSM MMs [236], which manifests BIC I mode and BIC II mode when the incident wave is along the *x* and *y* directions, respectively. The asymmetric parameter is defined as *α* = (*l*_1_−*l*_2_)/(*l*_1_+*l*_2_) × 100 %. As given in Figure 19(a) and (b), if *α* is 6.8 %, for the *x*-polarization, EIT peak corresponds with an MQ moment, which can be regarded as a hybrid of two electric quadrupolar moments. In this case, BIC I mode can be regarded as a bound state in the continuum of electric dipolar radiation, which results from the coupling of the MQ and ED moments. However, if the polarization is along the *y* direction, the BIC II mode is excited, and the resonant peak and dip corresponds with two opposite MQ moments, respectively. Additionally, by varying the Fermi level of 3D DSM layer in the range of 0.15–0.20 eV, the resonant wavelengths of BIC I and BIC II can be modulated in the range of 34.85–31.82 μm and 35.54–32.32 μm, respectively. Correspondingly, the MoD of resonant wavelength are 8.69 % and 9.06 %, respectively.

Based on pairs of ring resonators with C_2_ symmetry, Huang et al. proposed 3D DSM metamaterials to achieve THz modulators, as given in Figure 19(c) [237]. The maximum transmission difference is more than 82 % by varying the Fermi level, while the resonant frequency keeps almost unchanged. As given in Figure 19(d), for the BIC mode, because of the structural symmetry protection, only a weak dipolar resonance is excited. However, as the asymmetric parameter increases, large amounts of opposite charges accumulate at each ring resonator and excites a circulation current distribution along each ring resonator, which acts like a magnetic quadrupole mode and results into a sharp quasi-BIC resonance. Based on the periodically C_4v_ structural symmetrically arranged DSM identical squares, as given Figure 19(e), B. Zhou et al. proposed a tunable BIC resonance in the THz region [238]. Different from other published articles, the asymmetric parameter is defined as *δ* = E_f1_−E_f2_. As given in Figure 19(f), if the square DSM patters have the same Fermi level, only the broad electric dipolar moment is excited. However, if the Fermi levels of the square resonators along the diagonal direction are 0.05 eV and 0.048 eV, the electric quadrupolar and octupolar moments with large *Q–factor* of about 100 is achieved.

**Figure 20:**
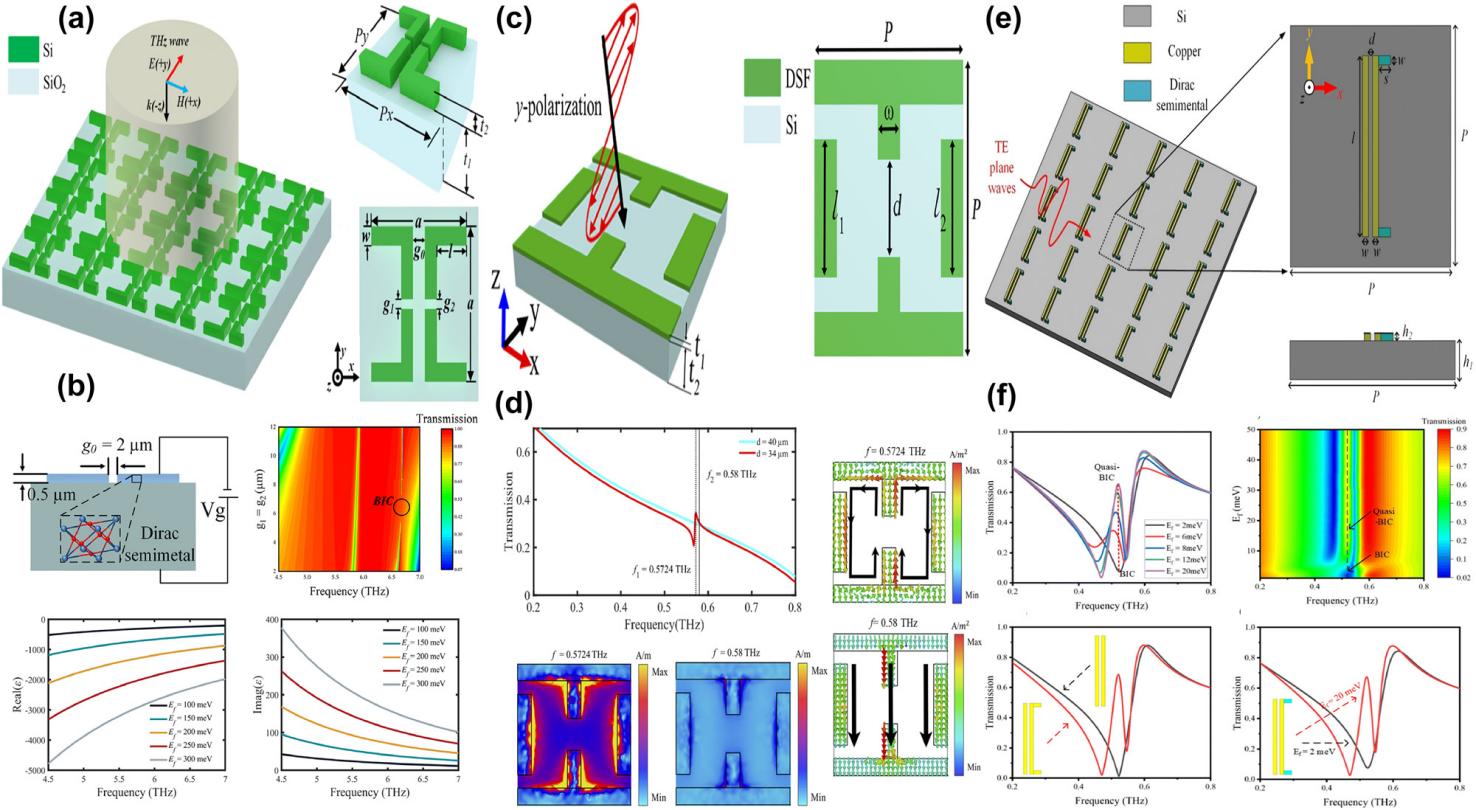
The 3D DSM stripe supported tuanble THz BIC metasurface with high *Q*-factor. (a) The sketch of the THz symmetric, all dielectric metasurface structure [239]. (b) The 3D DSM supported BIC MMs structure and the influence of the Fermi level on the transmission resonant curves [239]. Figures adopted with permission from Ref. [239] under License CC BY 4.0. (c) The schematic diagram for the proposed DSM “T-I” shaped resonator pair [240]. (d) The transmission, magnetic field and surface distribution for the quasi-BIC and BIC modes [240]. Figures adopted with permission from Ref. [240] under License CC BY 4.0. (e) The sketch of the proposed dimer DSM metasurface [241]. (f) The influences of Fermi levels on the transmission curves, electric fields, and surface current [241]. Figures adopted with permission from Ref. [241] under License CC BY 4.0.

Based on an open-slit, U-shaped all-dielectric MM resonators, as given in Figure 20(a), Liu et al. proposed a novel device with three BICs modes in the THz region, and the types of BIC degradation can be controlled by changing the structural parameters [239]. The asymmetry is defined as *α* = *d*/*l*, where *d* is the shift distance, and *l* is the length of the inner arm. The maximum sensitivities of accidental quasi-BIC and FOM reach more than 1717 GHz/RIU and 16670. Additionally, if the DSM layer is inserted into the MMs, a tunable sensor is achieved, as shown in Figure 20(b). When the Fermi level of DSM layer changes in the scope of 0.1–0.3 eV, the frequency shift range varies in the scope of 48–132 GHz. It should be noted that the sensor sensitivities at different Fermi levels are quite different, thus suitable Fermi levels can be chosen according to the target sensitivity.

By utilizing the 3D DSM supported a pair of “T-I” shaped resonators, they also proposed a tunable transmission MMs structure, which sustains two types of BIC modes, as given in Figure 20(c) and 20(d). The S–P BIC resonance is achieved by changing the structural symmetry, and the accidental BIC resonance is realized by adjusting the structural parameters. Those two kinds of BIC have a common resonant mode, while two LC resonances are coupled with them. The *Q–factor* for the BIC mode reaches about 175, and maintains a large value about 60 when the Fermi level is in the range of 0.35–0.98 eV. When the Fermi level increases from 0.05 eV to 0.98 eV, the transmission modulation depth is about 80 % [240]. As given in Figure 20(e), based on a two-layer structure with symmetric Cu bars with 3D DSM protruding in the upper layer, J. X. Gao* et al.* also proposed a BIC MMs in the THz region [241], by breaking the symmetry of resonators, a high *Q–factor* of more than 140 is achieved. When the Fermi level varies in the scope of 0.0–0.02 eV, the maximum transmission is larger than 0.61, and the according MoD is about 85 %. The proposed DSM MMs devices are utilized to achieve the dynamic near-field display, as given in Figure 20(f). When the Fermi level is low, *e.g.* 2 meV, the DSM MMs structure is in the “off” state. However, if the Fermi level is large enough, *e.g.* 20 meV, the proposed DSM structure is considered as a metal MMs with broken structural symmetry, the devices is in the “on” state. Using the well-designed 20 × 20 array, each resonator can be individually controlled by an external gate voltage, and the characters are displayed conveniently by adjusting the bias voltage.

**Figure 21:**
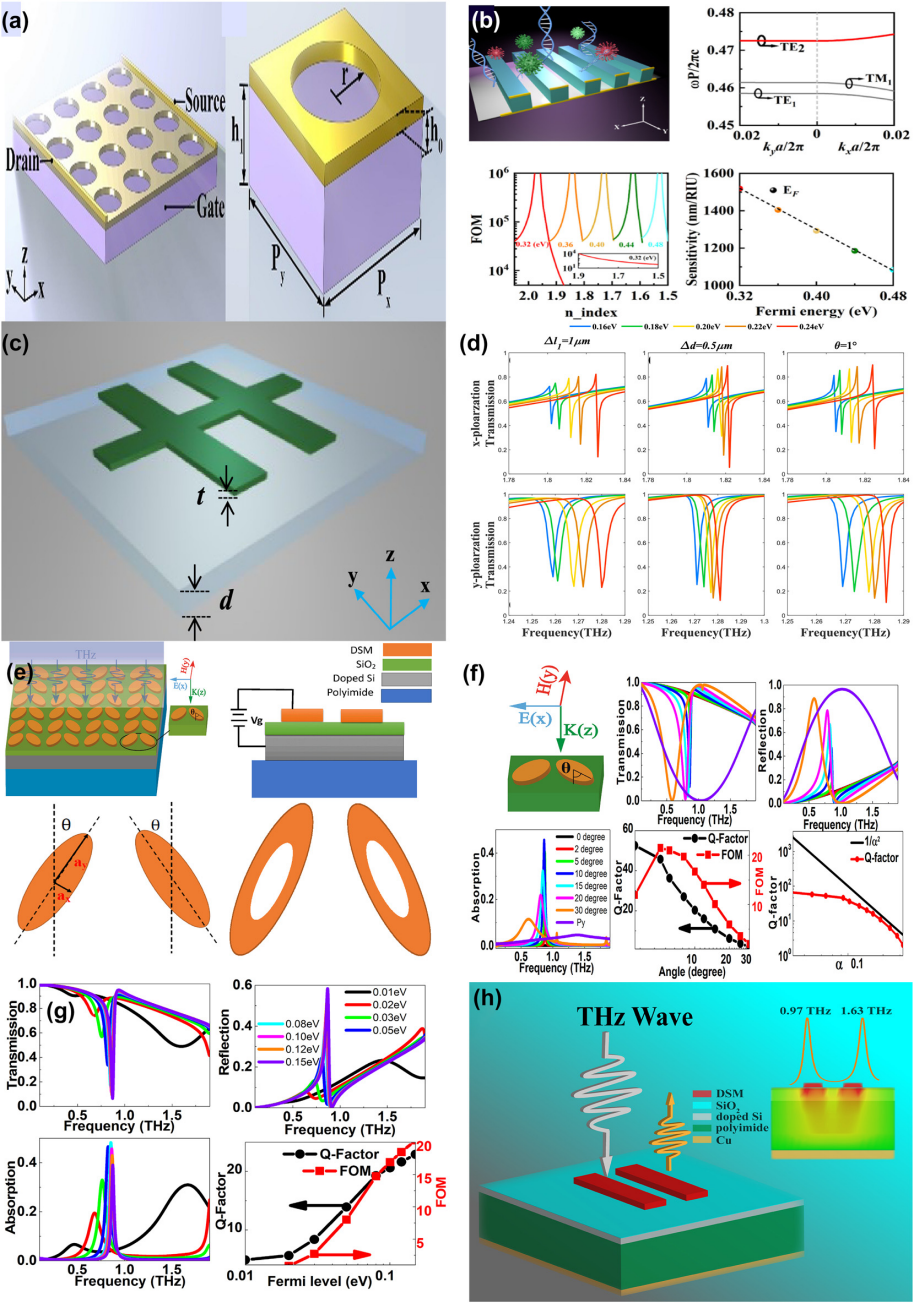
The tuanble high *Q*-factor THz BIC metasurface with subwavelength hole arrays and stripes. (a) The sketch of the 3D DSM subwavelength hole arrays [242]. Adopted with permission from Ref. [242]. Copyright 2023, Elsevier. (b) Schematic of a multi-biosensing system based on 1D periodic grating, which was composed of a Dirac semimetal, calculated TE and TM bands along the *k*_x_ and *k*_y_ axes, sensor FOM variation with the background environment refractive index and the corresponding sensor sensitivity for the different Fermi energies [242]. Figures adopted with permission from Ref. [242]. Copyright 2025, Elsevier. (c) The sketch of the proposed 3D DSM double elliptical MMs structures [243]. Figures adopted with permission from Ref. [243] under Licensed CC BY 4.0. (d) The influences of structural parameters and Fermi level on the transmission curves. Figures adopted with permission from Ref. [243] under Licensed CC BY 4.0. (e) The sketch of the proposed 3D DSM double elliptical MMs. Figures adopted with permission from Ref. [244] under Licensed CC BY 4.0. (f)-(g) The influences of the asymmetric parameter and Fermi levels on the DSM double elliptical MMs [244]. Figures adopted with permission from Ref. [244] under Licensed CC BY 4.0. (h) The sketch of the 3D DSM double stripes MMs absorbers [245]. Figures adopted with permission from Ref. [245]. Copyright 2024 Wiley‐VCH GmbH.

As given in Figure 21(a), based on the periodic hole arrays, B. Hou et al. investigated different types of BICs mode [242]. The results show that the sensor sensitivity is about 158 GHz/RIU, and the FOM of BIC modes increases from 202 to 663 when the Fermi level is adjusted from 0.20 to 1.0 eV. In 2025, as given in Figure 21(b), by utilizing 1D periodic grating, in 2025 they also proposed a high *Q–factor* sensor based on the topological quasi-BIC resonance of DSM [242], which shows that a diverging *Q–factor* (10^6^–10^9^) and extremely high FOM (more than 10^6^) over a wide range of dielectric environments. For instance, if the Fermi level is 0.32 eV, the *Q–factor* shows a peak with a large value of 10^9^, when the refractive index of environments changes in the range of 1.95–2.05. When the Fermi level of DSM layer increases from 0.32 eV to 0.48 eV, the sensitivity is changed in the range of 1,100–1,500 nm/RIU. The physical mechanism of this topological quasi-BIC resonance is based on the reciprocating motion of two pairs of BICs in the *k*_x_ and *k*_y_ high-symmetry lines of the momentum space, and these two pairs of BICs can be merged and generated by varying the Fermi energy of the Dirac semimetal.

Based on the 3D DSM trimer stripes resonators, as given in Figure 21(c), Q. G. Wang *et al*. proposed a polarization sensitive S–P BIC resonance with a large *Q–*factor of more than 7600 [243]. The results manifest that the quasi-BIC resonance mainly stems from the ED moment and is excited by shift the DSM bars along the *x*, *y* directions or rotating one of the horizontal bar with an angle of θ. Furthermore, when the Fermi level changes in the scope of 0.16–0.24 eV, the above-mentioned three methods, the BIC resonance frequencies can be modulated in the range of 1.802–1.826 THz, 1.811–1.822 THz, and 1.805–1.825 THz for the *x*-polarization, or 1.259–1.280 THz, 1.270–1.281 THz, and 1.269–1.284 THz for the *y*-polarization, which can be found in Figure 21(d). The sensing sensitivities for the *x*- and *y*-polarizations are 0.406 THz/RIU and 0.267 THz/RIU, while the values of FOM are 418.8 RIU^−1^ and 232.6 RIU^−1^, respectively. Additionally, based on the double elliptical resonators, X. Y. He et al. proposed a 3D DSM BIC resonant structure in the THz region, as given in Figure 21(e), the sharp resonant dip is excited by changing the rotated angle of the double elliptical resonators [244]. As the tilted angle increases, the resonant strength becomes stronger, and the resonant frequency dip manifests an obvious red shift. If the tilted angle changes in the range of 2°–30°, the amplitude and frequency MoD are 99.56 % and 33.36 %, respectively. At small tilted angle, the overall radiative loss is suppressed significantly, and the *Q*–factor is larger than 60. As the tilted angle increases, the *Q*–factor reduces. However, the resonant strength becomes stronger with the increase of the tilted angle. Thus, the FOM shows a peak at certain angle, about 5°–8°. Figure 21(f) shows the effects of Fermi levels on the resonant curves. As Fermi level increases, the resonant strength grows stronger and the resonant dip indicates a blue shift. At small Fermi level, the transmission resonant is not very obvious. With the increase of Fermi level, DSM layer shows better plasmonic properties, the reflection contribution dominates, the loss reduces. Additionally, with the modified configuration of elliptical resonator, another transmission resonant dip is excited and indicates a red shift with the increase of the permittivity of the dielectric filling material, which can be found in Figure 21(g). The resonant strength becomes weaker with the increase of the permittivity of dielectric filling material, and the reflection dip also indicates a red shift. Additionally, based on the double and triple 3D DSM stripes, S. L. Liu et al. proposed a tunable multi-frequency MMs absorber, as given in Figure 21(g) [245], which manifests near net absorption at the resonant frequencies of 0.97 and 1.63 THz by utilizing the double DSM stripes. When the length of the right stripe increases from 30 μm to 55 μm, a high *Q*–factor is larger than 40. When the Fermi level varies in the scope of 0.01–0.15 eV, the amplitude and frequency MoD are 77 % and 27 %, respectively. Additionally, with the triple DSM stripes, the proposed DSM MMs structure sustains the resonant peaks at the frequencies of 0.23 THz, 0.345 THz, and 0.46 THz with the absorption peak values of 0.997, 0.940, and 0.779.

### 3.2 Novel emerging materials supported BIC metamaterials

Apart from above-mentioned graphene and 3D Dirac semimetals, nowadays there emerged many other kinds of novel materials, such as transition metal dichalcogenides (TMD) [246], [247], [248], [249], [250], [251], [252], [253], [254], [255], [256], [257], [258], [259], [260], [261], [262], [263], borophene [264], [265], [266], [267], [268], and Weyl semimetal [269], [270], [271], [272], [273], [274]. These novel materials inhibit many interesting phenomena and boost the development of BIC metasurfaces. For instance, characterized by affordable price, easy availability, and a direct band gap, atomically thin monolayer TMD exhibits considerable binding energies, strong luminescence and excitonic response at room temperature, which is emerged as a promising class of novel materials for optoelectronics and nonlinear optics. Additionally, due to the inherent non-centrosymmetry properties, TMD monolayer possesses large nonlinear optical properties, such as second-harmonic generation, which is recognized method to determine the crystalline structure, the presence of defects, and the number of atomic layers.

**Figure 22:**
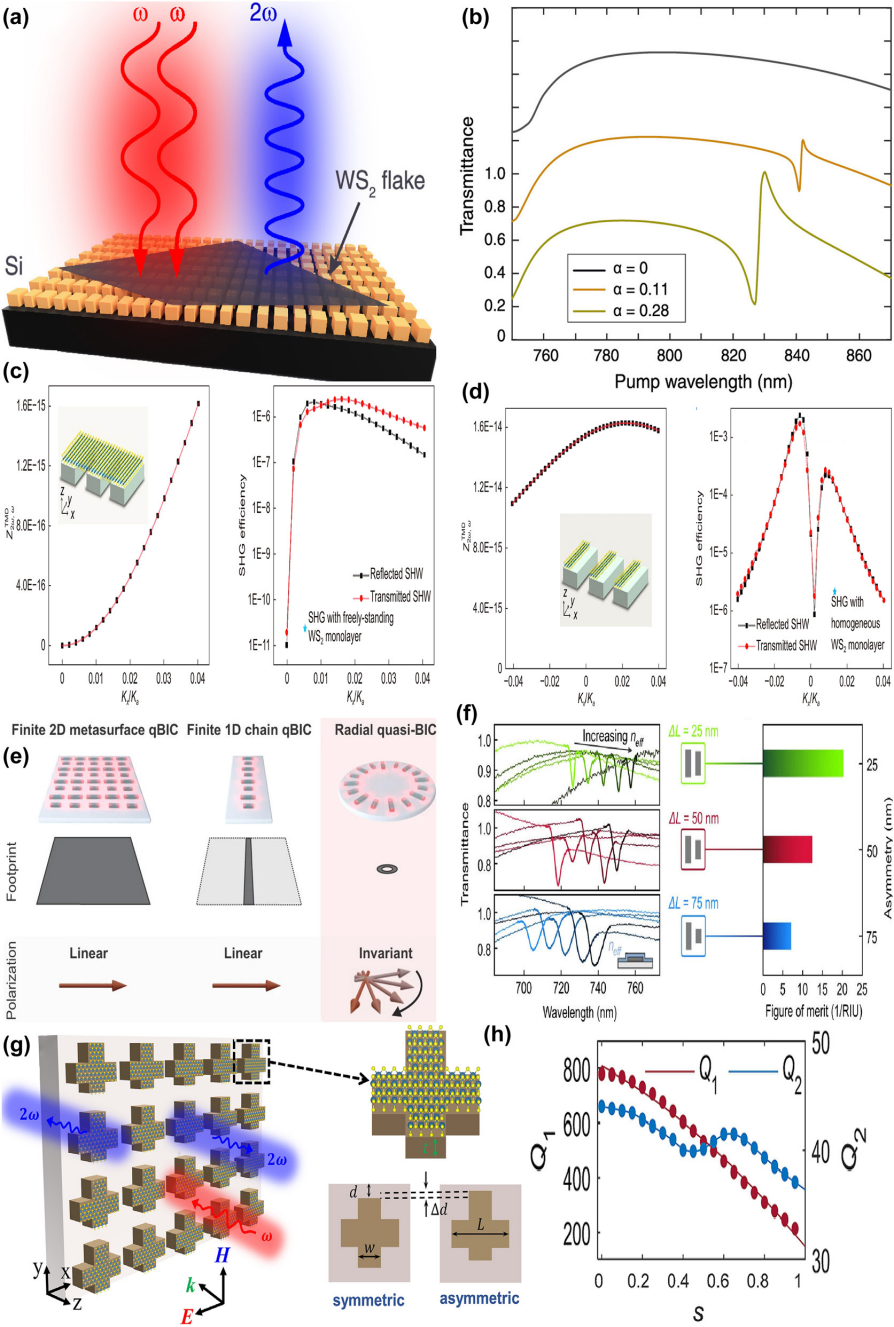
The WS_2_ monolayer supported enhanced nonlinear effect metasurface with BIC resonance. (a) Hybrid photonic structure for enhanced harmonic generation, schematic of SHG from a WS_2_ monolayer placed on top of a Si metasurface composed of a square array of bar pairs [246]. (b) Simulated evolution of the MMs transmittance with respect to the meta-atom asymmetry [246]. Figures adopted with permission from Ref. [246]. Copyright 2020 American Chemical Society. (c) Spatial overlap coefficient and SHG efficiency with a homogeneous WS_2_ on top of the photonic grating slab [247]. (d) Spatial overlap coefficient and SHG efficiency with a patterned WS_2_ on top of the photonic grating slab [247]. Figures adopted with permission from Ref. [247] under Licensed CC BY 4.0. (e) The comparisons of the footprint and polarizations for the 2D metasurfaces, 1D chains, and radial BIC concept [248]. Optical transmittance spectra of three different asymmetries ΔL with R = 1.5 µm covered with different thicknesses of conformal SiO_2_ thin films and the corresponding figure of merit for bulk refractive index sensing [248]. Figures adopted with permission from Ref. [248] under Licensed CC BY 4.0. (g) Schematics of a nonlinear metasurface with monolayer MoS_2_ on top of its cruciform-shaped silicon unit cell [249]. (h) Quality-factors of the quasi-BICs versus asymmetric parameter [249]. Figures adopted with permission from Ref. [249] under Licensed CC BY 4.0.

However, the second-harmonic generation (SHG) signal from a TMD monolayer is usually weak, and the overall frequency conversion efficiency is limited by the atomic length of the interaction with light, leading their practical applications into a bottleneck. To solve this problem, metasurface based quasi-BIC resonance with high *Q*–factor can be utilized to boost the nonlinear properties of a TMD monolayer device. Recently, there is some novel research in this aspect. For instance, by integrating a uniform WS_2_ with an asymmetric dimer Si all-dielectric brick, as given in Figure 22(a), in 2020 N. Bernhardt *et al. *proposed a quasi-BIC resonance supported enhancement of SHG [246]. The asymmetric parameter *α* is defined as the ratio of the width difference to the width of the larger bar. As the value of *α* increases, the transmission resonant dip becomes broad and deeper, which can be found in Figure 22(b). The dissipation of this WS_2_–Si metasurface hybrid structure mainly includes the radiative loss rate *γ*_rad_ and the *γ*_par_ originated from the material and fabrication imperfections. On the condition that the critical coupling condition (*γ*_rad_ = *γ*_par_) was met, the field enhancement factor (|*u*|^2^ = |A|^2^/E_0_^2^, the resonant amplitude of quasi-BIC resonance, *E*_0_ is the amplitude of the pump electric field) increases linearly with the *Q*–factor. Since SHG intensity is proportional to the field enhancement factor |*u*|^4^, when the asymmetric parameter is 0.28, the reflection spectrum shows an obvious dip with a *Q*–factor about 35, which is sufficiently large to achieve the SHG enhancement. For the proposed WS_2_–Si hybrid metasurface, at designed operating wavelength of 832 nm, the SHG enhancement is about 1140 times relative to the results of WS_2_ on top of a flat Si film. By integrating a homogeneous MoS_2_ with a dielectric GaP grating slab, in 2022 P. L. Hong et al., proposed a dual BICs resonance, *i.e.* a TE-type BIC resonance with fundamental wave at the wavelength of 1560 nm and TM-type BIC resonance with second harmonic wave at the wavelength of 780 nm [247], as given in Figure 22(c). GaP is a highly transparent in a broad spectral window from near-infrared to the visible spectral region. With a tilted angle to excite the dual quasi-BIC resonances, the maximum value of SHG efficiency about 2 × 10^−6^ is reached, more than 4 × 10^4^ times of that for the freely standing WS_2_. To improve the SHG efficiency, the WS_2_ patterns are partially covered by the GaP dielectric gratings to reinforce the overlap coefficient. Due to the mirror symmetry breaking caused by the patterned WS_2_-GaP hybrid structure, the SHG efficiency indicates obvious asymmetric behavior. For a negative incident angle, the peak value of SHG efficiency reaches more than 2 × 10^−3^, which is more than 4 × 10^7^ times of that of free standing WS_2_ monolayer, while for a positive incident angle, the enhancement is about 2 × 10^−4^. The further improvement of the SHG efficiency is limited by the intrinsic absorption of WS_2_ monolayer, and Figure 22(d) shows that the limited *Q–factor* for TE-type and TM-type BIC resonance reaches about 24000 and 8000, respectively. To solve the problem of the fabrication of intense arrays of polarization sensitive resonators, L. Kuehner et al. proposed radial quasi-BIC resonance distributed EM modes by tailoring the asymmetric dielectric rod pair resonators in a ring shape [248], as given in Figure 22(e). This proposed radial BIC metasurface provides polarization-invariant and tunable high-*Q* resonance with strongly enhances near fields in an ultracompact footprint as low as 2 μm^2^, 50 times small footprint compared to extended 2D metasurfaces. Composed of 12 resonators, the proposed radial ring quasi-BIC structure sustains polarization-invariant high *Q*–factor resonance with high surface sensitivity in a compact footprint and efficiently coupled with the incident light to form radial quasi-BIC resonance. The symmetry-broken ring structures are fabricated from amorphous Si with a thickness of 120 nm, and the length of a base rod is 335 nm with the width of 115 nm. The asymmetric parameter is introduced by reducing one rod of the dimer, and the optimum value of Δ*L* = 25 nm is utilized to include the effect of the nanofabrication accuracy. Pronounced transmission resonance shifts for different asymmetric parameters are detected, which can be found in Figure 22(f). This radial quasi-BIC metasurface also indicates good spectral polarization invariance, the *Q*–factor is larger than 200 even the rotation angle is 15°. In addition, by integrating with the TMD MoS_2_ layer, the proposed radial BIC structure is utilized to enhance light–matter interaction between metasurface and 2D excitons, the asymmetry parameter ΔL of 50 nm is adopted to balance the high near-field enhancement and sufficient resonant modulation. With the full spatial overlap between the micron-sized MoSe_2_ layer and the ultracompact ring geometry, a fivefold SHG intensity enhancement is demonstrated, arising from the near-field coupling of the MoSe_2_ monolayer with the radial BIC mode. By covering a monolayer MoS_2_ on a metasurface consisting of a square array of cross-shaped Si blocks, J. T. Wang *et al. *demonstrated a giant SHG phenomenon in the near-IR spectral region [249], as given in Figure 22(g). Due to significant localization and enhancement of optical near-field of quasi-BIC resonance, the nonlinear optical interaction of MoS_2_ is enhanced significantly. By shifting one arm of the cross resonator to introduce the asymmetric parameter (s = δ_d_/d), double quasi-BIC resonant peaks are observed. For this Si–MoS_2_ hybrid structure, the quasi-BIC resonance with high *Q–factor* is designed at the fundamental frequency (FF, 350 THz) and second harmonic frequency (SHF, 700 THz), which enhances the SHG magnitude three orders of magnitude than that of a suspend MoS_2_ layer. Additionally, as the asymmetric parameter increases from 0 to 1, the value of *Q–factor* at FF and SHF were changed in the range of 200–800 and 400–620, respectively, *i.e.* the quasi-BIC resonance at FF is more sensitive to the asymmetric parameter, as given in Figure 22(h).

**Figure 23:**
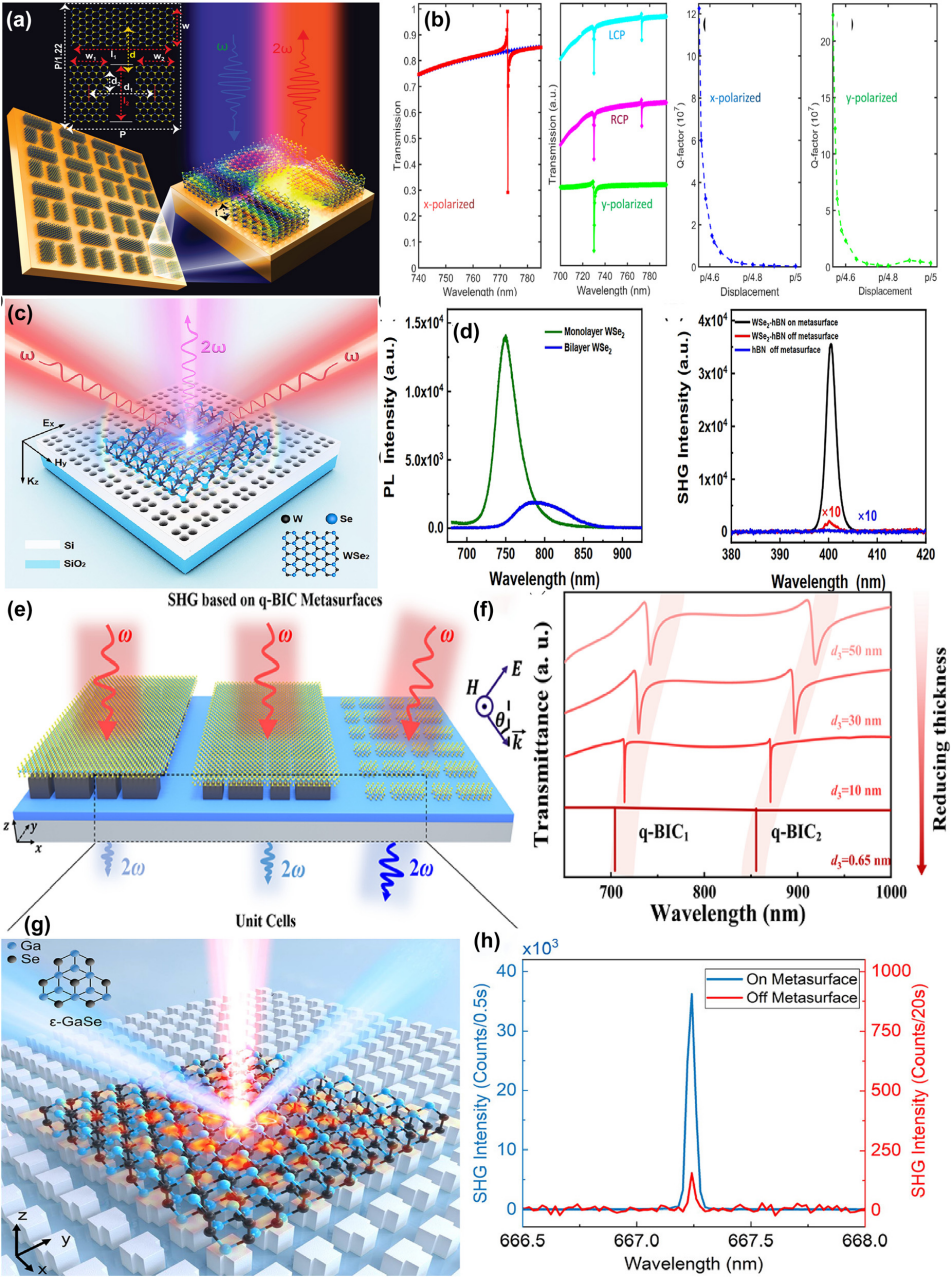
The WSe_2_ and MoS_2_ monolayer supported enhanced nonlinear effects metasurface with BIC resonance. (a) Schematic diagram of Π-shaped TMD metasurface [250]. (b) Transmission spectra and *Q–factor* of suspended structure at under *x*-polarized electric field, *y*-polarized, RCP and LCP [250]. Figures adopted with permission from Ref. [250] under Licensed CC BY 4.0. (c) Schematic of SHG from a WSe_2_ monolayer placed on top of the multiple-hole Si-metasurface fabricated on a quartz substrate [251]. (d) PL spectra of the hybrid metasurface-WSe_2_ structure and the measured spectra of the SHG from WSe_2_ monolayer, on the metasurface (black), on the bare quartz substrate (red), and the SHG from the bare hBN off metasurface (blue) [251]. Figures adopted with permission from Ref. [251] under Licensed CC BY 4.0. (e) Schematic diagrams for the evolution from bulk to monolayer quasi-BIC metasurfaces [252]. (f) Transmittance as a function of MoS_2_ thickness, where *α* = 0.9 [252]. Figure adopted with permission from Ref. [252] under Licensed CC BY 4.0. (g) Illustration of SHG from a GaSe flake laid on a Si metasurface consisting of a periodic square lattice of T-shaped pillars [252]. (h) Measured spectra of the SHG from GaSe flake [253]. Figures adopted with permission from Ref. [253]. Copyright 2021 American Chemical Society.

By depositing Π-shaped multilayer transition metals dichalcogenides metasurface on a dielectric substrate, N. Muhammad et al. investigated the quasi-BIC resonances at different thicknesses of meta-atoms in the near-IR spectral region [250], as given in Figure 23(a). The results show that the metasurface maintains the BIC in both homogeneous and non-homogeneous mediums with high *Q*–factor. As the long bar of Π-shaped resonator move closer to the two short ones, the coupling strength increases, and a quasi-BIC resonant dip appears near the wavelength of 772 nm for the *x*-polarized incident wave. However, for the *y*-polarized incident light, the according resonant strength of quasi-BIC mode reduces. Furthermore, for the left-circularly and right-circularly polarized incident fields, another leaky mode appears at relatively short wavelength of 730 nm, which can be found in Figure 23(b). The hetero-symmetric structure is highly tunable and upholds the BIC at different thickness of meta-atoms. Additionally, near the quasi-BIC resonance, the SHG conversion efficiency is about 1.47 × 10^−4^, much larger than that of reported literature. By integrating a WSe_2_ layer with a multiple-hole Si metasurface, as given in Figure 23(c), P. W. Ren et al. proposed a novel means to enhance the second harmonic generation by utilizing quasi-BIC resonance with a flat band [251], which results from a vast enhancement in the state density and the local electric field in the optical mode. The all-dielectric Si metasurface is constructed by periodically arranging subwavelength resonators, consisting of four circular holes with the radius of 50 nm and a thickness of 250 nm. The asymmetric parameter *α* is defined as the shift distance of the four circular holes move diagonally toward the center of resonator. When the value of *α* was 5 nm, an obvious transmission dip is observed at the wavelength of 810 nm. As the value of *α* increases, the resonant strength becomes stronger, and the resonant dip manifests a blue shift. The nonlinear medium of monolayer WSe_2_ is transferred to the Si metasurface with an ultra-thin h-BN layer acts as the protected layer. As the four holes moves toward the center of the unit cell and the value of parameter *α* increases, the ED and TD moments are effectively excited by external pumping. Though limited by the absorption loss of silicon material and radiative loss, the *Q*–factor of quasi-BIC resonance reaches more than 100 in the near-IR spectral region. Additionally, a dramatic SHG enhancement more than 170 times is achieved, and the SHG intensities increases with the pumping power before saturation of 2 mW, as given in Figure 23(d). Based on a pair of geometrically asymmetric MoS_2_ ribbons as resonators to excite two quasi-BIC resonances with large *Q*–factor, Y. N. Xie et al. achieved a large enhancement factor of SHG in both visible and ultraviolet spectral region [252], as given in Figure 23(e). The rectangular lengths in the *x*-direction for the ribbons are *l* and *l*+Δ*l*, and the asymmetric parameter is defined as *α* = Δ*l*/*l*. The dual quasi-BIC resonances are attributed to the low-order and high-order guided mode resonances at an oblique incident wave. As given in Figure 23(f), as the thickness of MoS_2_ ribbon decreases, the transmission dips for the dual quasi-BIC resonances manifest blue shifts, the resonant dips become sharp with larger *Q–factor*. For instance, the *Q*–factors for quasi-BIC I and quasi-BIC II reach more than 1.0 × 10^6^ and 1.4 × 10^6^, respectively. The resonant curves with ultrahigh *Q–factor*s are usually associated with the significant enhancement of the localized electric fields and are beneficial to the improvement of nonlinear polarization. As the incident wave angle increases, those dual quasi-BIC resonant dip get close to each other and merged into one when the incident wave is 10.5°. In this case, the quasi-BIC I resonance is transformed into the distinct F–W BIC mode. The monolayer MoS_2_ asymmetric ribbon metasurface with strong coupling quasi-BIC resonance exhibits a significant enhancement factor of about 2.8 × 10^10^, 10^4^ times larger than that of Si metasurface. Accordingly, the SHG conversion efficiency is drastically enhanced, about 5.8 % at the wavelength of 383.3 nm.

2D gallium selenide (GaSe) also shows an exceptional performance of high-efficiency second harmonic generation with a giant second order susceptibility χ^(2)^ of 1,700 pm/V under telecom-band excitation, which can be effectively enhanced by increasing the layer numbers. By laying GaSe flakes on the Si metasurface consisting of a periodic T-shaped resonator arranged in a square lattice, as given in Figure 23(g), Z. J. Liu et al. proposed a giant enhancement of SHG owing to the coupling to quasi-BIC resonance [253]. However, due to the presence of centro-symmetry, the even-order nonlinear susceptibilities of Si metasurface usually vanished. Integrating 2D materials into the Si metasurface offers an efficient way to excite the even-order nonlinearity. By arranging Si resonators with symmetric defects and breaking the rotational symmetry protection, quasi-BIC resonances is excited with a large *Q–factor* of more than 8911. Since the ε-type stacking and peculiarity of absence of centro-symmetry, the intensity of SHG is linearly scaled with the layer numbers. Thus, the SHG efficiency is improved significantly with 24 layers of 2D GaSe flake deposited on the Si metasurface. With the high *Q*–factor of the quasi-BIC resonances and large χ^(2)^ of stack layers of GaSe, as given in Figure 15(h), a giant (9400-fold) enhancement of SHG from the reflection spectra of the GaSe-Si metasurface is achieved, about three orders of the magnitude higher than previous published results.

**Figure 24:**
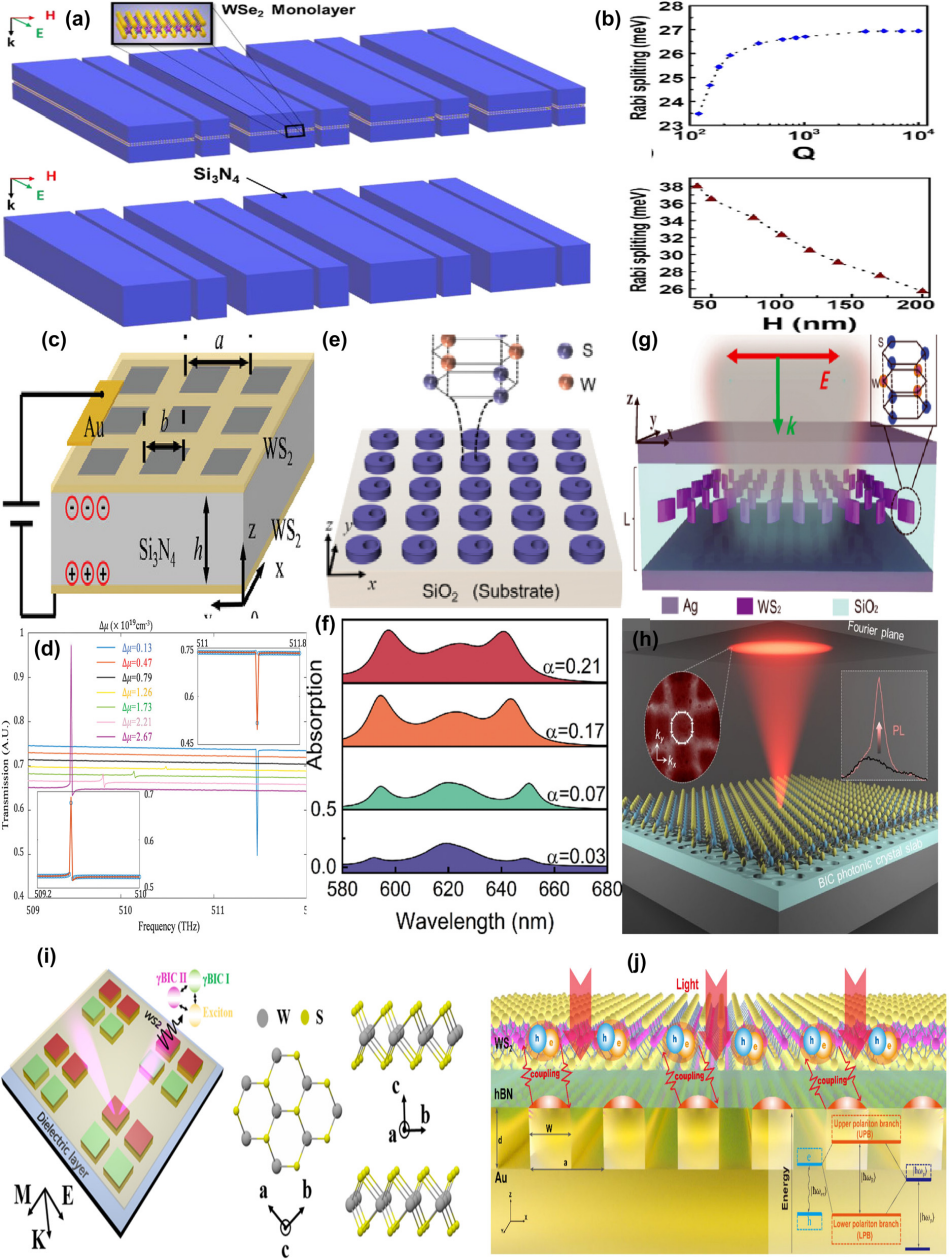
The TMD-MM hybrid structure supported BIC resonance with large Rabi-splitting energies and *Q*-factors. (a) Schematic 3D drawing of Si_3_N_4_ NW array and WSe_2_ inside SiN nanowire array with small slit [254]. (b) The Rabi-splitting as a function of the *Q*–factor and the height of the nanowires, respectively [254]. Figures adopted with permission from Ref. [254]. Copyright 2021 Wiley‐VCH GmbH. (c) Schematic of the proposed electrically tunable BIC in 2D material integrated photonic crystal slab, the lattice constant a = 365 nm and thickness of Si_3_N_4_ = 320 nm [255]. (d) Calculated transmission spectra depending on carrier density, the insets were the enlarged transmission spectra when Δμ = 0.13 × 10^19^ and Δμ = 2.67 × 10^19^, respectively. The circles were the results of the numerical simulations [255]. Figures adopted with permission from Ref. [255]. Copyright 2022 Wiley‐VCH GmbH. (e) 3D schematic diagrams of the coupling system, an elliptical bulk WS_2_ array embedded in an F–P cavity. The thickness of the silver film is 20 nm [256]. (f) Absorption spectra of the bulk metasurface for different asymmetry parameters under the energy detuning of zero [256]. Figures adopted with permission from Ref. [256]. Copyright 2023, APS. (g) Sketch of the system, nanodisk metasurfaces of bulk WS_2_ were illuminated by a normally incident plane wave [257]. Figures adopted with permission from Ref. [257]. Copyright 2023, APS. (h) Schematic of photoluminescence emission of the monolayer WS_2_ on the BIC photonic crystal slab [258]. Figures adopted with permission from Ref. [258]. Copyright 2025 Wiley‐VCH GmbH. (i) 3D schematic of a permittivity-asymmetric tetramer metasurface with and mirror geometric symmetry that support multiple quasi-BICs [259]. Figures adopted with permission from Ref. [259]. Copyright 2025, APS. (j) The schematic of the strong coupling between a plasmonic BIC and TMD excitons based on a 1D grating platform supporting for the formation of exciton polaritons in a monolayer WS_2_ at room-temperature [260]. Figures adopted with permission from Ref. [260]. Copyright 2025 American Chemical Society.

Additionally, TMD-BIC MM hybrid structure is also utilized to obtain the large Rabi-splitting energies via strong coupling, enhance the photoluminescence (PL) intensity, achieve the high *Q*–factor of Fano resonance. For example, in 2021 by integrating TMD monolayer with the asymmetric Si_3_N_4_ slit, I. A. M. Ai-Ani *et al. *proposed the strong couplings associated with the quasi-BIC resonance [254]. As given in Figure 24(a), by changing the slit width and gap width, the TE_21_ mode dominates with MD moment and the TE_31_ mode dominates with the electric TD moment are excited, respectively. The *Q–factor* of the former decreases with the slit width, while that of the latter shows a peak when the gap width is about 120 nm. The strong coupling can be realized between the exciton in a TMD monolayer and quasi-BIC mode by varying the incidence angle, period of the grating, the width of the slit, and the position of the slit for S–P BIC resonance. The Rabi-splitting is strongly associated with the location of TMD monolayer, the *Q*–factor of resonator, and the thickness, which can be found in Figure 24(b). The maximum Rabi-splitting is achieved when TMD monolayer positioned at the maximum of the electric field. The coupling strength is improved with thinner nanowires. With suitable structural parameters and TMD materials, the different Rabi-splitting energies are achieved to 38 (1L-WSe_2_), 65 (1L-WS_2_), 40 (1L-MoSe_2_), and 60 meV (1L-MoS_2_) at room temperature. Owing to the effective area of the TMD monolayer is larger than that in the 3D dielectric metasurface, the 1D dielectric meta-grating provides stronger coupling to the TMD monolayers. As given in Figure 24(c), C. Zhao et al. proposed an electrically tunable BIC resonant by utilizing a novel 2D materials with integrated PC slab [255], which consists of a Si_3_N_4_ slab covered by few layer WS_2_ films at the top and bottom, the whole structure is etched with a square lattice of holes. With the bias voltage, the Fermi level and carrier concentration of WS_2_ film is adjusted and achieved the manipulation of accidental BIC resonances around Γ point. Furthermore, by reducing the in-plane symmetry from C_4Z_ to C_2Z_, the merging BIC resonance can be shifted to different locations in momentum space. By distorting the square hole, an asymmetric merging state is achieved and can be controlled by both the carrier density and distorting magnitude. As the carrier concentration Δμ increases, the accidental BIC mode approaches the symmetry protected BIC. For the oblique incident wave, the transmission curve exhibits Fano peaks when the carrier concentration difference increases from 0.13 × 10^19^ to 2.67 × 10^19^ cm^−3^ and indicates an obvious redshift. However, the Fano peak disappears at 0.79 × 10^19^ cm^−3^, and corresponds with the accidental BIC resonance in Γ-X direction, which can be found in Figure 24(d).

In 2023, M. B. Qin et al. proposed a strong coupling between excitons and quasi-BIC resonances in bulk TMD WS_2_ in the visible spectral region [256], as given in Figure 24(e). A hole at a fixed distance D = R/2 away from the center of the disk, and the asymmetry parameter is defined as the ratio of the hole area to the nano-disk area. The transmission curve shows an obvious dip at the wavelength of 620 nm and mainly results from the magnetic dipolar quasi-BIC mode, which self-hybridizes with the excitons and resulted into strong light–matter interaction enhancement without an external cavity. As the asymmetric parameter increases, the resonant curves become broad and the transmission dip indicates an obvious blue shift, the height and period of resonators shows the opposite trend. This strong coupling is confirmed by the larger Rabi splitting energy of 159 meV, and an obvious anti-cross behavior in the absorption curves. At small value of asymmetry parameter *α*, the number of photons is smaller than excitons and the surplus exciton plays an important role. As the asymmetric parameter increases, the number of photons increases and contributed more to the coupling, resulting into the absorption enhancing obviously, which can be found in Figure 24(f). At the same year, P. Xie et al. demonstrated the enhancement of self-hybridization of excitons and BIC resonance with the assistance of F–P cavity [257]. As given in Figure 24(g), the double tilted elliptical bulk WS_2_ with the thickness of 25 nm are embedded into a silver F–P microcavity. An obvious S–P quasi-BIC resonance is excited by changing the tilted angle in the range of 0–40°, which mainly results from the EQ and MD moments. The electric field at the resonant peak is greatly enhanced by a factor of 640, which plays a key role in the strong mode coupling. The results show that the self-hybridized BIC-excitons coupling strength can be dramatically enhanced with the assistance of a F-P cavity. A giant Rabi splitting up to 240 meV is achieved, about twice as high as the results in the metasurface system. This strong field enhancement is explained by the excellent spatial overlap of the F–P cavity and quasi-BIC mode, which makes use of the full coupling of the intra- and interlayer excitons in the WS_2_ layer. By integrating a monolayer of tungsten disulfide with a dielectric photonic crystal slab, J. Lee et al. proposed a spatial control of photoluminescence polarization in the near-IR spectral region [258]. As shown in Figure 24(h), consisting of a periodic circular nano-hole etched into silicon nitride and the radiative *Q–factors* are 195 and 415, respectively. The energy confinement factor is approximately 4.25 times larger when the 2D material is positioned at the center of the PhC slab compared to that when it is on the slab surface. As given in Figure 24(i), based on a permittivity-asymmetric tetramer metasurface with same structural parameters, S. Y. Wang et al. proposed a WS_2_ induced quasi-BIC-exciton in the visible spectral region [259], which supports three high *Q–factor* resonant modes. Three quasi-BIC reflection peaks are observed at the wavelengths of 587 nm, 616 nm, and 629 nm, which mainly result from the magnetic TD, EQ, and electric TD and magnetic moments. The gap between nanotubes affect the resonant curves significantly. As the gap decreases, the *Q–factor* of quasi-BIC resonance I decreases, while the *Q–factor* of BIC II and BIC III resonance increase. For the WS_2_-nanotubes hybrid metasurface, as the gap decreases, the reflection peak indicates obvious redshift owing to the large permittivity of WS_2_ about 18. A WS_2_ monolayer deposited on the C_4v_ symmetric metasurface results into strong coupling between the multiple quasi-BIC resonance and the excitons, which is adjusted by the height and gap of the tetramer. By depositing on a silicon dioxide substrate, the PhC slab is designed to support the BIC near the exciton resonance of WS_2_. Due to the S–P BIC resonance spectrally overlap with the excitonic resonance of the monolayer of tungsten disulfide, the polarization singularity with spatially winding linear polarization in the PL emission from monolayer WS_2_. Additionally, the coupling between the photonic band and monolayer WS_2_ induces the directional emission and enhances the PL intensity. The changing the nano-cube height, strong coupling occurred when the resonant energies of BIC mode approximates the excitons energy of WS_2_, indicating three Rabi splitting. Furthermore, as the gap between nano-cubes increases, the coupling strengths of BIC modes and WS_2_ excitons continuously enhances, which is attributed to the reduction of the effective mode volume of the hybrid states.

As given in Figure 23(j), C. Luo et al. proposed a plasmonic BIC resonance to achieve strong coupling with the excitons in a monolayer WS_2_ in the visible spectral region by utilizing a 1D grating platform [260], which composes a parallel Au stripes on a Si substrate. With the momentum-space imaging and PL technique, the authors experimentally confirm that the strong coupling and the Rabi splitting energy is about 93 meV. With suitable stripe width and period of 1D grating, the BIC resonant frequency is matched to the exciton energy of WS_2_. The *Q–factor* indicated a peak of around 500 at the Γ point in the momentum space when the *γ* (*γ* = *γ*_p_/*ω*_p_, *γ*_p_ was the metal loss, and *ω*_p_ is the bulk equipartition excitation frequency) is 0.003. Additionally, with a hBN isolation layer, the PL intensity improves significantly, which can be explained below. As a high-quality dielectric medium, the hBN layer enhances the local electric field around the WS_2_ layer and the light-exciton interaction. Besides the reduction of interface scattering and nonradiative recombination, the hBN layer is also helpful to keep the crystalline quality of WS_2_ layer and promote the efficiency of the phonon-scattering processes, making the Raman resonant peak more distinct.

**Figure 25:**
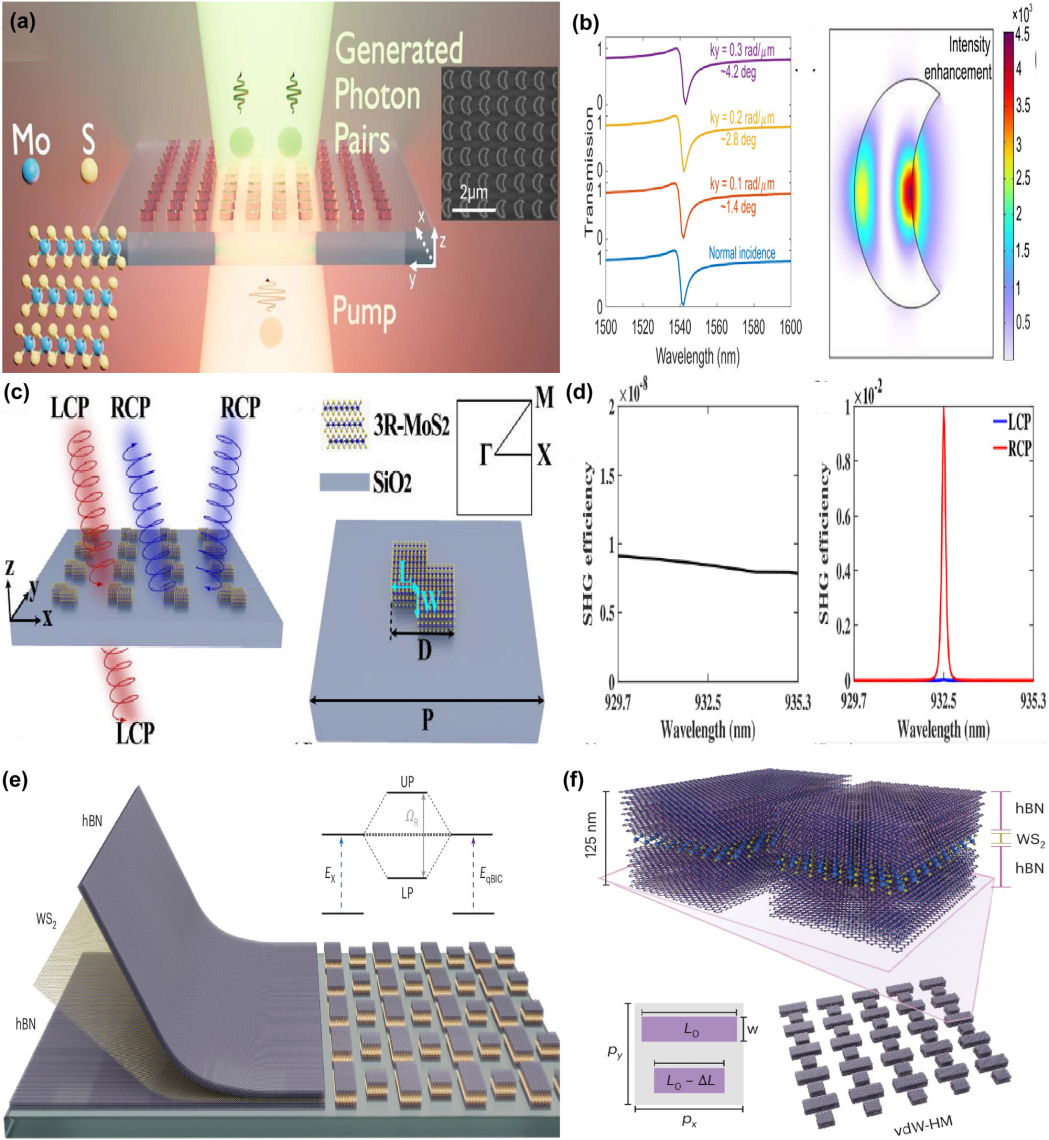
The 3R-MoS_2 _and WS_2_ monolayer supported enhanced nonlinear effects metasurface with BIC resonance. (a) The sketch of photon-pair generation from a 3R–MoS_2_ metasurface. [261]. Figures adopted with permission from Ref. [261]. Copyright 2024 American Chemical Society. (b) The influences of the different incident angles on the transmission, and the scattered electric field intensity enhancement at the resonant wavelength of 1,540 nm [261]. Figures adopted with permission from Ref. [261]. Copyright 2024 American Chemical Society. (c) The schematic of the proposed Z-shaped 3R–MoS_2_ metasurface under illumination of circularly polarized light [262]. Figures adopted with permission from Ref. [262]. Copyright 2025 AIP. (d) The SHG efficiency in the unpatterned 3R–MoS_2_ film and Z-shaped metasurfaces under LCP and RCP illumination [262]. Figures adopted with permission from Ref. [262]. Copyright 2025 AIP. (e) The illustration of ultrathin optical cavities in van der Waals heterostructure metasurfaces, composed of a WS_2_ semiconductor monolayer encapsulated between two thin hBN layers [263]. Figures adopted with permission from Ref. [263] under Licensed CC BY 4.0. (f) The schematic of the WS_2_-hBN hybrid metasurface unit cell, the total height of hBN/WS_2_/hBN layers was 125 nm, w is the nanorod width, *L*_0_ is the base rod length and Δ*L* is the asymmetry factor [263]. Figures adopted with permission from Ref. [263] under Licensed CC BY 4.0.

With the merits of high refractive index, low optical losses, and large second-order susceptibility, 3R-phase MoS_2_ emerges as a compelling choice to design BIC metasurface. For instance, as given in Figure 25(a) [261], by utilizing etched circular 3R–MoS_2_ disk, a quasi-BIC resonance is excited at the wavelength of 1,540 nm with a *Q*-factor about 120, mainly resulting from the contribution of MD mode. The period length and thickness of MoS_2_ resonator are 790 nm and 222 nm, respectively. This asymmetric round disk indicates a high *Q*-factor within a broad emission angle of 3°, and the electric fields intensity enhancement of up to 4.5 × 10^3^ times for y-polarized field components, which can be found in Figure 25(b). Consequently, the proposed asymmetric vdW resonators indicates a 20 folds enhancement of spontaneous parametric down-conversion rates compared to unpatterned thin films and an ultrahigh brightness enhancement about 400 times. Furthermore, a significant enhancement of SHG intensity is observed as the excitation wavelength, exhibiting an approximately 90-folds enhancement compared to the off-resonance position. In addition, in 2025 by arranging the Z-shaped MoS_2_ resonator in a square lattice, as given in Figure 25(c), J. R. Wang et al. proposed a highly efficient and chirality-sensitive SHG by coupling them to BICs resonances [262]. Under oblique incidence, the *Q*-factor of quasi-BIC resonance reaches more than 104 at the wavelength of 932.5 nm, and the SHG efficiency is about 1 %, over six orders of magnitude larger than those for the MoS_2_ multilayers. Furthermore, the proposed chiral MMs structures manifest a near-unity circular dichroism, the magnitude difference in SHG efficiency between RCR (0.01) and LCP (2.95 × 10^−5^), as given in Figure 25(d). Furthermore, the periodic helicity inversion of elliptical polarization states results into an azimuthal-angle-dependent chirality inversion and achieved an efficient switching of SHG output between LCP and RCP excitation. By encapsulating a WS_2_ layer with two thin hNB layers, as given in Figure 25(e), L. Sortino et al. experimentally demonstrated an ultrathin dielectric cavity in van der Waals heterostructure metasurfaces in the visible spectral region [263], which eliminates the requirement for bulky external cavity systems. With asymmetric WS_2_ dimer resonator to break the symmetry, as given in Figure 25(e), an S–P BIC resonance is excited with a *Q*-factor larger than 100 and indicates a Fano-like line shape. Furthermore, the quasi-BIC resonance can be shifted across broad spectral ranges by using a lateral scaling factor S, which can be found in Figure 25(f). Thus, the cavity–emitter detuning parameter *δ* = E_qBIC_−E_X_, can be manipulated conveniently, where E_qBIC_ and E_X_ are the qBIC mode and WS_2_ exciton energy, respectively. There exists strong coupling between WS_2_ excitons and hBN polaritons at room temperatures with optimized structural parameters, which are confirmed by both the absorption and emission curves. Furthermore, this hBN/WS_2_/hBN rod pairs metasurface exhibits strong nonlinearities, a saturation of the strong-coupling regime at ultralow fluences of <1 nJ cm^−2^ was observed, three orders of magnitude lower than in previous 2D cavity systems. The proposed WS_2_-vdW heterostructure paves a novel way for low-threshold polariton lasers and high-speed, low-energy optical integrated photonic devices.

In addition, borophene is also an important typical 2D material and arouses the researcher’s attentions. It has a stronger bond-forming ability among 2D material family, a high carrier density (≈10^19^ m^−2^) and huge anisotropic optical properties.

**Figure 26:**
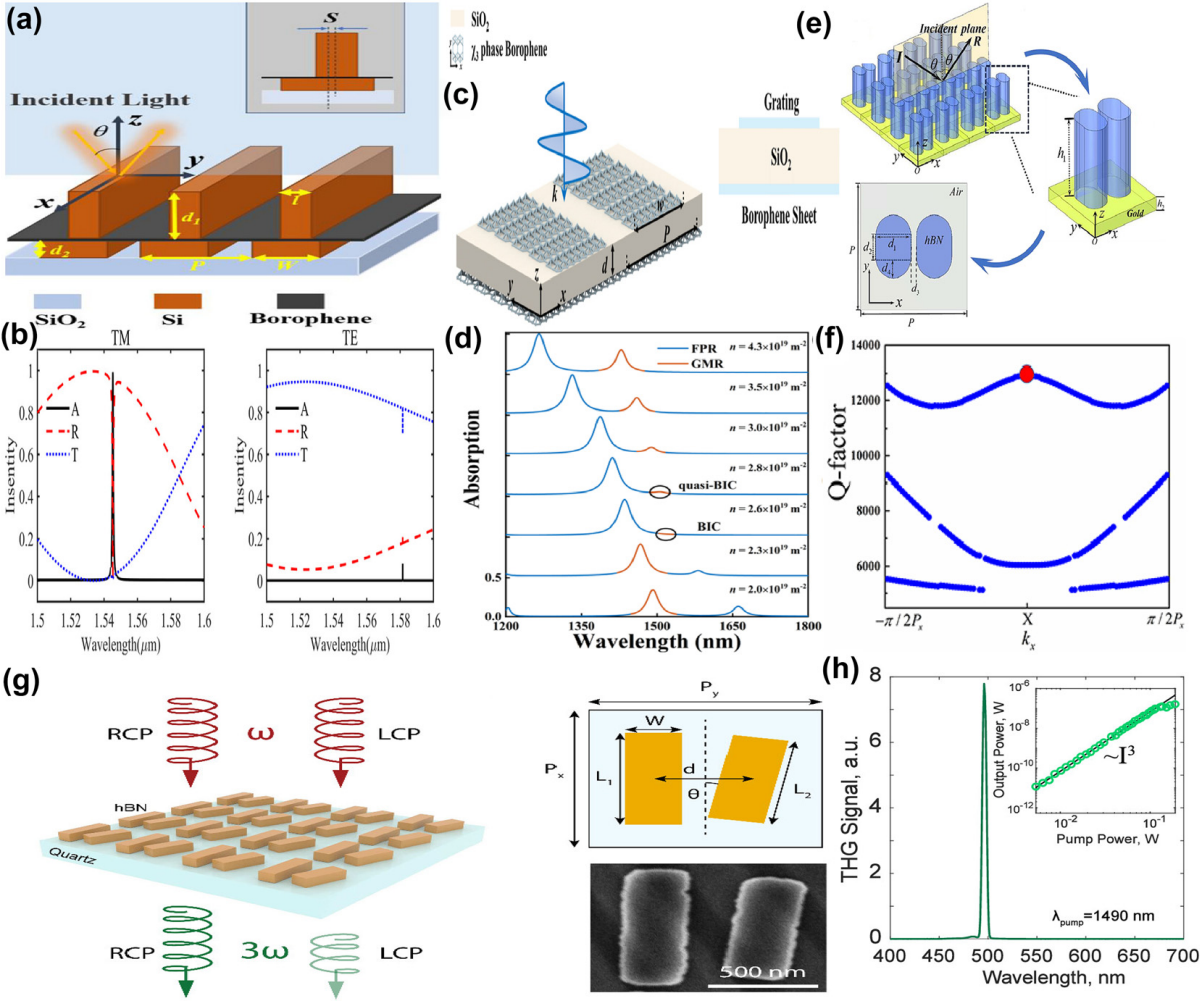
The borophene and hBN membrane supported nonlinear metasurface with BIC resonance. (a) Schematic of the proposed borophene-Si grating supported perfect narrow band absorbers [264]. (b) Absorption, refection, and transmission spectra calculated by rigorous coupled-wave analysis at S = 19 nm for TM and TE polarized lights [264]. Figures adopted with permission from Ref. [264]. Copyright 2023 Wiley‐VCH GmbH. (c) Structure diagrams of the Borophene supported absorption system, and its cross-sectional view in one period [265]. (d) The dynamic modulation process of the F–W BIC (w = 66 nm) by designing the carrier density of the borophene grating [265]. Figures adopted with permission from Ref. [265] under Licensed CC BY 4.0. (e) The 3D structure and unit cell top view of the hBN metasurfaces. The rectangle had a length of d_1_, a width of d_2_, and a spacing of d_3_, the height of the hBN pillar is h_1_, and the thickness of the gold substrate is h_2_ [266]. (f) The *Q*–factor of electric field out of the plane mode varying with frequency wave vector [266]. Figures adopted with permission from Ref. [266] under Licensed CC BY 4.0. (g) The sketch of the asymmetric nanobars hBN supported nonlinear chiral metasurface [267]. Figures adopted with permission from Ref. [267]. Copyright 2024 American Chemical Society. (h) The spectrum of the third harmonic from the metasurface pumped at a wavelength of 1,490 nm, and the inset showed the output power−pump power curve of the third harmonics [267]. Figures adopted with permission from Ref. [267]. Copyright 2024 American Chemical Society.

By inserting a single layer borophene into two Si gratings, the thickness and width of the upper and bottom Si gratings were different. In 2023 J. Z. Liu and Y. W. Liu proposed a perfect narrow-band absorber in the near-IR spectral region [264], as given in Figure 26(a). With a dislocation distance *S* between the upper and bottom Si gratings, the absorption efficiency is enhanced and a sharp absorption peak is observed owning to the excitation of quasi-BIC resonance. As the asymmetric parameter increases, the radiative rate varies, on the condition that the dissipation loss of the borophene is met to the radiative rate, the critical coupling condition is satisfied, the maximum absorption, about 100 %, can be achieved. The *Q–*factor of the sharp absorption curve is about 2570. As for the effects of the structural parameters, the absorption curve is more sensitive to the thickness of the upper Si grating layer. Due to the anisotropic properties of the borophene, the proposed hybrid absorber is very sensitive to the polarization of the incident wave. For the TM incident wave, the absorption curve indicates a sharp peak at the wavelength of 1.5451 μm with a value about 99.18 %, the HWHM is about 0.62 nm, while for the TE mode, the absorption peak is about 8.15 % and indicates a red shift, which can be found in Figure 26(b).

As given in Figure 26(c), G. D. Liu’s group proposed a F–W BIC resonance to achieve perfect absorption with dual peaks in the communication bands based on the multilayer structure of borophene gratings-SiO_2_ layer-uniform borophene layer [265], which supports F–P resonance and guide mode simultaneously. The results manifest that by adjusting the borophene gratings and carrier concentration, the tunable F–W BIC resonance is observed. As the carrier density decreases, these two modes both indicate redshifts, on the condition that the carrier concentration is 2.6 × 10^19^m^−2^, the F–W BIC resonance is formed owing to the destructive interference between them, which can be found in Figure 26(d). These two modes exchanges energy before and after strong coupling, indicating that the F–W BIC is dynamically modulated by designing the carrier density of the borophene grating. In addition, with the weak angular dispersion of the intrinsic flat-band characteristics, for the TM polarized wave, this proposed borophene metasurface displays high absorption even at an incidence angle of up to 70°. However, for the TE mode, the effective excitation of borophene surface plasmon is hindered and the absorption is almost zero. Thus, the absorption switching between complete absorption and complete transparency is achieved by changing the phase difference between two coherent beams. With a tensor permittivity, hBN demonstrates strong anisotropic properties and is utilized to fabricate hyperbolic metamaterial, *e.g.* by depositing hBN pillar with cross-sections of semi-ellipses and rectangle on a gold layer, M. Z. Sun et al. proposed a symmetry-protected BIC resonance in the near-IR spectral region, as given in Figure 26(e) [266]. By introducing the asymmetric parameters δ_1_ and δ_2_, the S–P BIC resonance is excited and exhibits a high *Q–factor* about 13000 at the frequency of 44.3964 THz, which is found in Figure 26(f). Additionally, the multipolar theory of reflection spectra indicates the toroidal dipole mode plays a dominated role by breaking the symmetry. By depositing asymmetric hBN bars on the quartz substrate, P. Tonkaev et al. demonstrated a nonlinear chiral metasurface in the near-IR spectral region [267]. To excite the quasi-BIC resonance, the right bar of the vdW meta-atoms is shorter and rotates an angle relative to the axis of the bar, as given in Figure 26(g). The spectrum of the pumped THG at a wavelength of 1490 nm has a maximum at a wavelength of 497 nm, which indicates a maximum THG efficiency of 10^−6^ and a peak intensity of 10 GW/cm^2^. The THG signal strongly depends on the pump wavelength. At the quasi-BIC resonance, the THG signal is enhanced by about 3 orders of magnitude compared to the THG signal from the thin hBN film, accompanied by strong nonlinear circular dichroism at the resonances, as given in Figure 26(h).

Finally, consisting of an even number of nondegenerate band-touching nodes, Weyl semimetal [104], [271], [272], [273], [274], [275], [276], [277], [278], [279], [280], [281], [282], [283], [284], *e.g.* EuCd_2_As_2_, Co_3_Sn_2_S_2_, TaP, Mn_3_Sn, PtTe_2_, HgTe, NbP, is also an important kind of topological semimetals. Those Weyl semimetals show opposite chirality and are separated by a vector 2b in momentum space, which act as effective magnetic fields. With those peculiarity, Weyl semimetals also indicate potential candidates for the development of tunable BIC metasurface, *e.g.* in the design of polarizer and anisotropic modulators.

## 4 Conclusion and prospective

The recent progress of the graphene, 3D DSM and other novel topological semimetal (MoS_2_, borophene, GaSe) supported tunable BIC resonance based the metasurface structures, have been given and discussed, including the effects of Fermi levels, operation frequency regions, and different shapes of resonators. As an efficient tunable medium, graphene sustains strong BIC resonant curves in the near-IR and THz wave regions with different operation mechanisms. In the near-IR spectral region, graphene layer inhibits a lossy dielectric layer, and indicates the ENZ phenomenon on the condition that ℏω≈2μ_c_, ***a uniform graphene membrane is often utilized to enhance the BIC resonance***. However, for the THz waves, when intra-band transition dominates, the graphene layer acts as a thin metal layer and prevents the incident wave, ***graphene patterns are more preferable to develop tunable BIC resonance***, even if a uniform doped graphene layer is utilized, the tunable range of Fermi level is still in a small range, typically less than 0.10 eV. The fatal problem of graphene device is its thin thickness in the range of nanometer, which hinders the experimental fabrication and limits its further practical applications. Fortunately, 3D DSM surmounts the restriction of thickness and provides much more design of freedoms in the construction of tunable functional devices. Another point should be noted is that thanks to the large differences between the Fermi velocities along the in-plane and perpendicular directions, 3D DSM inhibits strong anisotropic properties and is very suitable for the design of polarizer and optical dichroism devices.

Nowadays there is emergence of other kinds of novel materials to develop tunable BIC metasurface, *e.g.* MoS_2_, GaSe, and Weyl semimetals. For instance, inhibiting the merits of cheap price, large exciton binding energies, and strong luminescence at room temperature, the TMD-MMs hybrid BIC structures are widely applied to enhance the nonlinear effects, together with the strong mode confinement of BIC resonance. The SHG intensity enhancement reaches two or three orders larger than the results without BIC resonances, and the maximum values of SHG conversion efficiencies are in the range of 1.47 × 10^−4^–2 × 10^−3^. Furthermore, due to the strong coupling between BIC resonances and excitons of TMD, the Rabi splitting energies reaches about 159 meV, and enhanced to 240 meV with the help of a metal F–P cavity. However, the limited operation waveband is a fatal existed problem, owing to the excitons of TMD locating in the visible or near-IR spectral region. Additionally, as a cousin of DSM, Weyl semimetal is also an important kind of topological material, inhibiting many exotic properties, such as chiral anomaly, magnetic monopoles in the crystal momentum space, the pronounced topologically stable chiral edge states, and good tunable properties thanks to its Fermi level susceptible to temperature, bias voltage. For instance, the permittivity tensors of WSM are asymmetric, the off-diagonal matrix terms are comparable to that of diagonal components. Thus, the magneto-optical parameter of WSM is three orders of magnitude larger than that of conventional materials. It can be concluded that cooperating with the design platform of BIC metasurface, TMD, GaSe, and WSM are very suitable for the design of anomalous Hall effects and chiral magneto-optical devices, such as efficient thermal emitters, polarizer and nonlinear optical devices.

From above discussions, it can be found that graphene supported BIC MMs resonance have been intensely investigated in a wide spectral region, *i.e.* the stage I as given in Figure 2(b), its operation mechanisms are clear and the fabrication techniques also relatively matured. In contrast, though shedding potential prospects to develop high performance devices, the explorations of the BIC devices associated with 3D Dirac semimetal, nodal-line semimetals, and Weyl semimetals, are relatively rarely owing to the limitation of experimental techniques, as the stage II and stage III shown in Figure 2(b). For instance, the production of high-quality 3D DSM or WSM films with large area remains a great challenge, together with the fabrication of tunable devices. Those topological layers are usually susceptible to the surface defects, strain vacancy, uneven doping and different kinds of substrates. Fortunately, with the assistance of molecular beam epitaxy, MOCVD, and magnetron sputtering, the fabrication of 3D DSM and WSM devices develop very quickly and meet the requirement of practical applications in the near future. In all, this work provides a useful reference guideline for researchers entering the domain of DSM devices and building tunable functional devices in the fields of wireless communications, security detection, and design of functional devices, *e.g.* filters, modulators, switch, and polarizers.
